# Synthesis of Novel Saccharin Derivatives

**DOI:** 10.3390/molecules22040516

**Published:** 2017-03-23

**Authors:** Gregory M. Rankin, Sally-Ann Poulsen

**Affiliations:** Griffith Institute for Drug Discovery, Griffith University, Don Young Road, Nathan, Queensland 4111, Australia; rankin.greg13@gmail.com

**Keywords:** sulfonamide, metal binding group, zinc binding group, click chemistry, Cu(I)-catalyzed azide alkyne cycloaddition, saccharin, triazole, glycoconjugate

## Abstract

The synthesis of saccharin (1,2-benzisothiazol-3-one-1,1-dioxide) derivatives substituted on the benzene ring has seen limited development despite the longevity of this compound’s use as an artificial sweetener. This type of saccharin derivative would however present attractive properties for the development of new bioactive, drug-like small molecule compounds. Here we report the derivatisation of the benzene ring of saccharin using Cu(I)-catalyzed azide alkyne cycloaddition (CuAAC) to synthesise a diverse library of novel saccharin-1,2,3-triazole conjugates. All library compounds retain the capability for interactions with biomolecules via the unmodified sulfonamide and lactam groups of the parent saccharin core heterocycle. The compounds also encompass alternate orientations of the 1,2,3-triazole heterocycle, thus further adding diversity to the potential hydrogen bonding interactions of these compounds with biomolecules of therapeutic interest. Our findings demonstrate that specifically functionalized derivatives of saccharin may be prepared from either saccharin azide or saccharin alkyne building blocks in high yield using CuAAC.

## 1. Introduction

**Figure 1:**
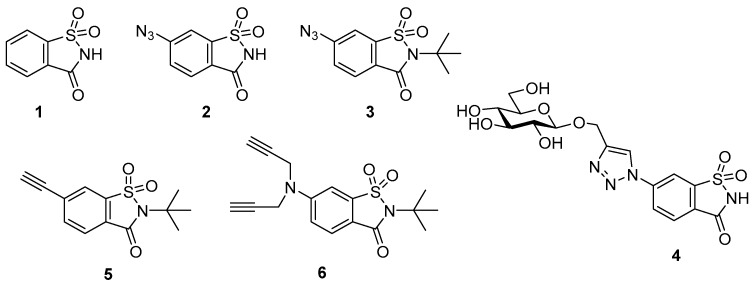
Saccharin **1**, 6-azidosaccharin **2**, *N*-*t*-butyl protected 6-azidosaccharin **3**, derivatised saccharin glycoconjugate **4** [10], and new saccharin alkyne building blocks **5** and **6**.

Several different synthetic routes have been applied to construct the heterocyclic compound 1,2-benzisothiazol-3-one-1,1-dioxide, commonly known as saccharin (**1**, Figure 1), a synthetic calorie-free sugar substitute [1,2]. Alkylation methods to prepare *N*- and *O*-substituted (carbonyl oxygen) derivatives of **1** are well established and these have provided researchers with the straightforward synthesis of novel and potentially bioactive molecules [3,4,5,6]. In addition to *N*- and *O*-alkylation, compounds derivatised on the benzene moiety of **1** are particularly desirable, as these compounds retain both the cyclic sulfonamide and lactam groups which can participate in strong noncovalent interactions with biomolecular targets such as enzymes. The synthesis of the latter derivatives is reliant on the application of synthetic acumen to introduce a latent handle on the benzene moiety of **1** for subsequent derivatisation; this modification’s methodology is however much less developed than the straightforward alkylation of **1**.

**Figure 2:**
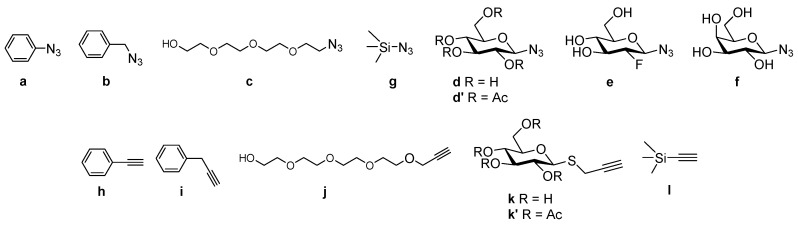
Partner azides (**a**–**g**) and alkynes (**h**–**l**) for preparing derivatives of **1** using Cu(I)-catalyzed azide alkyne cycloaddition (CuAAC).

Compound **1** is a weak acid, the measured p*K*_a_ of the cyclic sulfonamide NH hydrogen is 1.3 [7], and the anion of **1** readily forms complexes with metal(II) cations [8]. Klebe and Supuran first demonstrated that the metal binding characteristics of **1** could be utilised to target metalloenzyme inhibition, specifically the zinc metalloenzyme carbonic anhydrase (CA) [9]. We further elaborated this finding through the design and synthesis of a small library of compounds based on **1** that retain the cyclic sulfonamide core (allowing for zinc binding) with the addition of a ‘tail’ group to the benzene ring of **1** [10]. The ‘tail’ approach to develop CA inhibitors is well established, with the physicochemical properties of the ‘tail’ group as a key driver to CA inhibitor isozyme selectivity across the CA family, of which there are 12 catalytically active isozymes in humans [11]. Cu(I)-catalyzed azide alkyne cycloaddition (CuAAC or click chemistry) is the reaction between an azide (R-N_3_) and an alkyne (R-C≡CH) to form a 1,2,3-triazole [12]. We demonstrated that CuAAC is a robust and versatile approach to link a selected tail group to a CA zinc binding pharmacophore [13,14,15,16,17,18]. To achieve the synthesis of compounds derivatised on the benzene ring of **1** via CuAAC, we prepared two novel azides, 6-azidosaccharin **2** and *t*-butyl protected 6-azidosaccharin **3** (Figure 1) [10]. One compound from this initial study, the 1,2,3-triazole glycoconjugate **4**, exhibited remarkable selectivity for CA IX, a CA isozyme that underpins the survival of hypoxic tumour cells (Figure 1) [10]. In the protein X-ray crystal structure of **4** in complex with a CA IX mimic protein, as anticipated, **4** was found coordinated to the active site zinc via the cyclic sulfonamide anion, while the tail moiety of **4** contributed to further interactions with the outer rim of the CA IX-mimic active site residues [19]. Here we report further development of the derivatisation of the benzene ring of scaffold **1**. Specifically, we have substantially expanded the scope of CuAAC with the design and synthesis of the novel alkyne **5** and bis-alkyne **6**, complementary building blocks to the *t*-butyl protected 6-azidosaccharin **3** (Figure 1). The building blocks **3**, **5**, and **6** enable access to triazoles with a reversed arrangement of substituents, i.e., with the saccharin fragment as either the 4-substituent or the 1-substituent of the 1,2,3-triazole formed by CuAAC. This is important as the 1,4-disubstituted 1,2,3-triazole is a bioisostere of a *Z*-amide bond, hence the positioning of the H-bond donor and H-bond acceptor is also reversed with the use of complementary saccharin building blocks [20]. Derivatisation of **5** with a diverse panel of azides (**a**–**g**), derivatization of **6** with glycosyl azide (**d**), and the further derivatization of **3** with alkyne partners (**h**–**l**) is described. Partner azides (**a**–**g**) and alkynes (**h**–**l**) were selected to encompass variable physicochemical properties (Figure 2). All compounds retain the sulfonamide and lactam moieties of **1**.

## 2. Results and Discussion

In our previous study using 6-azidosaccharins, we established that it was preferable to undertake CuAAC reactions with *N*-*t*-butyl protected 6-azidosaccharin **3**, followed by removal of the *t*-butyl protecting group, over the more direct synthetic approach of using 6-azidosaccharin **2** [10]. Specifically, the ease of synthesis and isolated yield substantially improved when using **3** owing to the simplified reaction workup and product purification. We attributed these advantages to the blockade of metal complex formation between **3** and Cu^2+^ (from the CuSO_4_ used for CuAAC) by the *N*-*t*-butyl protecting group. Building on this experience we selected *N-t*-butyl-6-ethynyl-1,2-benzisothiazole-3-one-1,1-dioxide (*N*-*t*-butyl-protected 6-ethynylsaccharin **5**) as the target building block for CuAAC in the present study (Figure 1). Additionally, *N*-*t*-butyl-6-*N,N*-bis(prop-2-yn-1-yl)amino-1,2-benzisothiazole-3-one-1,1-dioxide **6** was selected as the bis-alkyne rather than the unprotected form without the *t*-butyl protecting group (Figure 1).

**Scheme 1:**
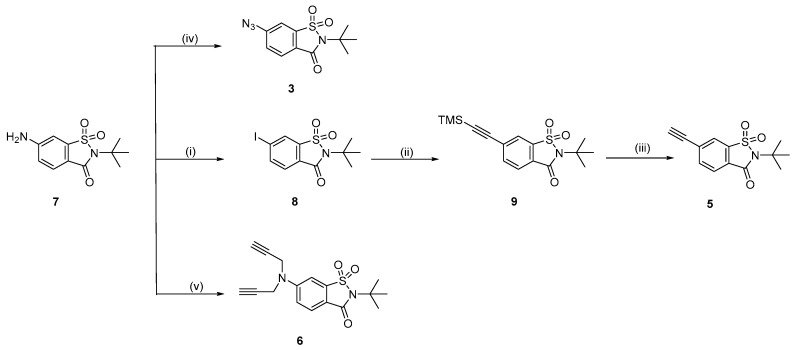
Synthesis of *N*-*t*-butyl-protected 6-azidosaccharin **3** [10], *N*-*t*-butyl-protected 6-ethynylsaccharin **5**, and *N*-*t*-butyl-protected bis-alkyne saccharin **6**. Reagents and conditions: (i) *p*TsOH.H_2_O, NaNO_2_, KI, CH_3_CN, 10–15 °C → room temperature (rt), ~1 h, 83%; (ii) Pd(PPh_3_)Cl_2_, CuI, TMSC≡CH, Et_3_N, 40 °C, 2 h, 95%; (iii) TBAF, AcOH, THF, 5 min, rt, 97%; (iv) *t*-BuNO_2_, TMSN_3_, 0 °C → rt, 16 h, 46% [10]; (v) Cs_2_CO_3_, propargyl bromide, dimethylformamide (DMF), 0 °C, 48 h, 75%.

The synthetic route to alkynes **5** and **6** and the earlier reported azide **3** have a common precursor, *N*-*t*-butyl-6-aminosaccharin **7** [10] (Scheme 1). Iodination of **7** with sodium nitrite and potassium iodide was achieved following a literature procedure used for iodination of similar aromatic and heterocyclic compounds to give the *N*-protected 6-iodosaccharin **8** in an 83% yield [21]. The Sonogashira cross-coupling reaction between **8** and ethynyltrimethylsilane **l** generated the trimethylsilyl protected alkyne **9** in high yield. Removal of the trimethylsilyl group of **9** under standard conditions of K_2_CO_3_ in methanol proceeded, however these conditions additionally caused ring opening at the C-N bond of the heterocycle. The successful removal of this silyl group was instead achieved using mild acidic reaction conditions (tetrabutylammonium fluoride (TBAF)/1% AcOH in tetrahydrofuran (THF)) to afford the target alkyne **5** in an almost quantitative yield. Next, to install the two terminal alkyne groups of **6**, the amino saccharin compound **7** [10] was treated with propargyl bromide (2.2 equivalents (equiv)) in the presence of Cs_2_CO_3_.

**Figure 3:**
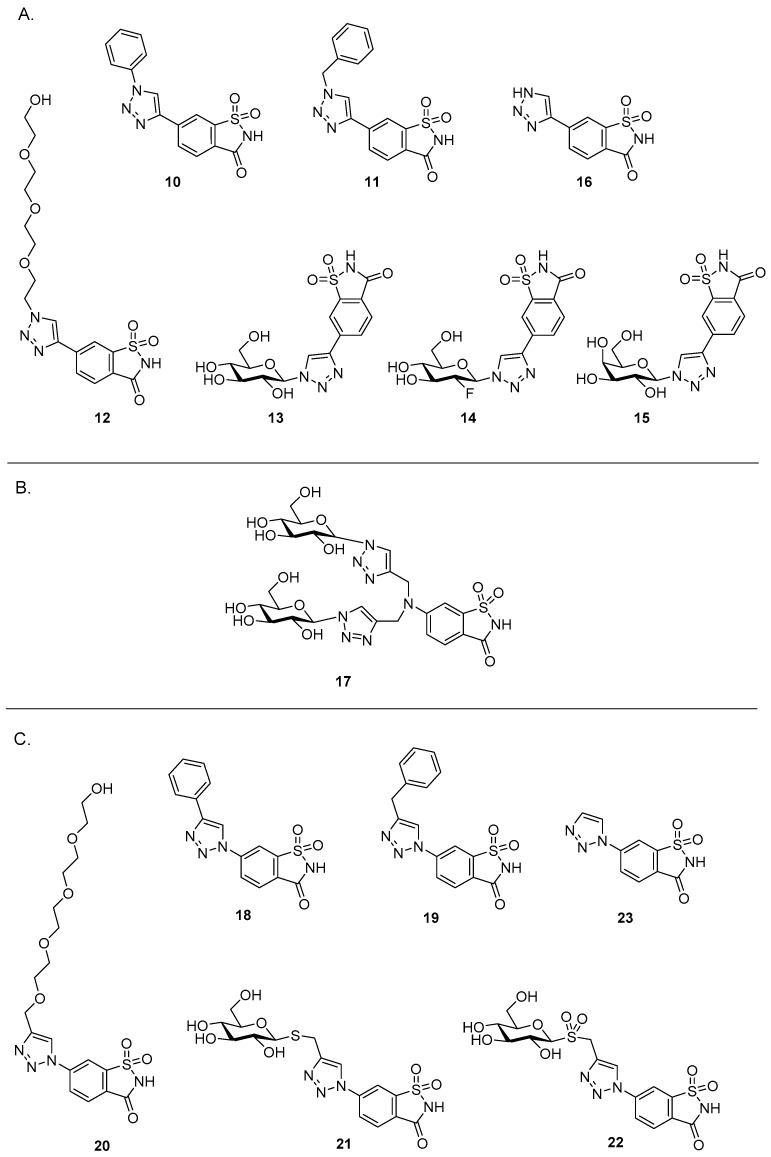
Target derivatives of **1** prepared using CuAAC. (**A**) Derivatives prepared from *N*-*t*-butyl-protected 6-ethynylsaccharin **5** and azide **a**–**g**; (**B**) Derivative prepared from *N*-*t*-butyl-protected bis-alkyne saccharin **6** and azide **d/d′**; (**C**) Derivatives prepared from *N*-*t*-butyl-6-aminosaccharin **7** and alkynes **h**–**l**. We have previously reported the phenyl derivative **18** while all other compounds are novel [10].

The target triazole derivatives of compound **1** for this study are shown in Figure 3. Compounds derived from alkyne **5** and azides **a**–**g** include the phenyl derivative **10**, benzyl derivative **11**, tetraethylene glycol (PEG) derivative **12**, sugar derivatives **13**–**15**, and unsubstituted triazole derivative **16** (Figure 3A). The ‘sugar coated’ bis-triazole saccharin derivative **17** (generated from **6** and azide **d/d′**) comprises two glucose moieties (Figure 3B). The novel target triazoles derived from azide **8** and alkynes **h**–**l** include the benzyl derivative **19**, PEG derivative **20**, sugar derivatives **21** and **22**, and unsubstituted triazole **16** (Figure 3C). We have previously reported the phenyl derivative **18** [10].

**Scheme 2:**
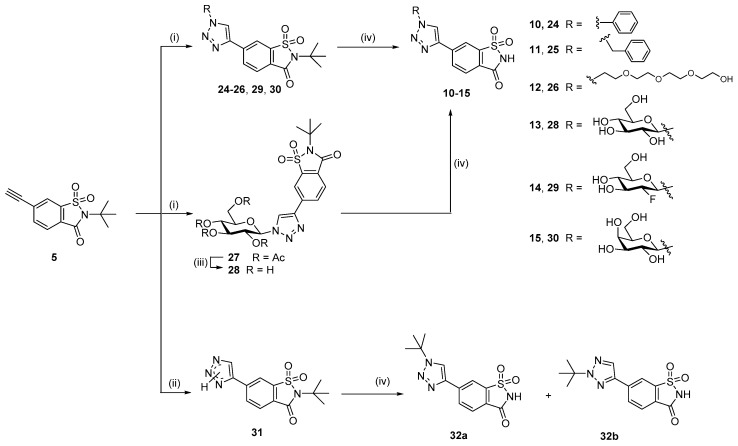
Synthesis of saccharin derivatives from *N*-*t*-butyl-protected 6-ethynylsaccharin **5**. Reagents and conditions: (i) **5** (1 equiv), azide (1 equiv), sodium ascorbate (0.4 equiv), CuSO_4_·5H_2_O (0.2 equiv), 1:1 *t*-BuOH:water, 45–50 °C, 2 h–overnight; (ii) **5** (1 equiv), TMSN_3_ (2 equiv), sodium ascorbate (0.4 equiv), CuSO_4_·5H_2_O (0.2 equiv), 1:1 *t*-BuOH:water, 45–50 °C, overnight; (iii) 8% HCl in MeOH, r.t., 90 h; (iv) TFA, reflux, 18 h.

**Scheme 3:**
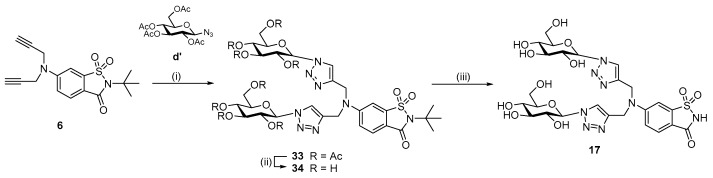
Synthesis of saccharin glycoconjugate **17** from bis-alkyne **6**. Reagents and conditions: (i) CuSO_4_·5H_2_O (0.2 equiv), 1:1 *t*-BuOH:water, 45 °C, 87%; (ii) 8% HCl in MeOH, r.t., 90 h, 92%; (iii) TFA, reflux, 18 h, 84%.

The reaction of **5** with azidobenzene **a** [22], benzylazide **b** [23], and PEG azide **c** [24] was carried out under typical CuAAC conditions (0.2 equiv of CuSO_4_∙5H_2_O and 0.4 equiv of sodium ascorbate, *t*-BuOH:water 1:1, 45 °C) to generate triazoles **24**–**26**, respectively, in 74%–97% yield (Scheme 2). Subsequent removal of the *N*-*t*-butyl group of **24**–**26** was achieved following reflux in trifluoracetic acid (TFA) for 18 h to furnish the target derivatives of **1**, triazoles **10**–**12**, respectively, in high yield (85%–99%). The reaction of alkyne **5** and 2,3,4,6-tetra-*O*-acetyl-β-d-glucopyranosyl azide **d′** [25] at 50 °C produced triazole **27** in high yield (90%). As we had concerns that the harsh basic conditions promote opening of the saccharin heterocyclic ring, the acetyl groups of **27** were hydrolysed using HCl in MeOH instead of the more usual Zemplén conditions of methoxide in MeOH [26], to afford **28** in a 94% yield, however a lengthy reaction time of 90 h was required (Scheme 2). The *N*-*t*-butyl group of **28** was removed with refluxing in TFA for 18 h to yield the target glycoconjugate **13** in high yield. The bis-triazole saccharin glycoconjugate **33** was prepared from bis-alkyne **6** and per-*O*-acetylated glucosyl azide **d′** [25] in high yield (87%) (Scheme 3). Deacetylation of **33** under acidic conditions (HCl in MeOH, 90 h) gave the free sugar **34**, and cleavage of the *N*-*t*-butyl group of **34** with TFA furnished the target bis-triazole saccharin glycoconjugate compound **17** (Scheme 3). As the acidic conditions to remove the acetyl groups of **27** and **33** required a prolonged reaction time (90 h), this prompted us to investigate an alternate route to synthesise the glycoconjugates **14** and **15**. This route employed free glycosyl azides **e** and **f**, instead of the corresponding per-*O*-acetylated glycosyl azides, to eliminate the need for deprotection of the sugar hydroxyl groups following CuAAC, thus removing the dependence on this synthetic bottleneck [25,27,28,29]. The reaction of alkyne **5** and free glycosyl azides **e** and **f** [25,27,28,29] via CuAAC proceeded smoothly to form intermediates **29** and **30** (Scheme 2). Subsequent removal of the *N*-*t*-butyl protecting groups of these intermediates by overnight refluxing in TFA afforded target glycoconjugates **14** and **15**, respectively. Next, CuAAC of azidotrimethylsilane (TMSN_3_) **g** with alkyne **5** gave the monosubstituted triazole **31** as an inseparable mixture of thermodynamically stable tautomers [30]. Cleavage of the *N*-*t*-butyl group of **31** using TFA furnished a mixture of **32a** and **32b**, where ^1^H nuclear magnetic resonance (NMR) and high resolution mass spectrometry (HRMS) analysis confirmed triazole N-alkylation, with the *t*-butyl group on either the N-1 (**32a**) or N-2 (**32b**) of the triazole ring. Although the reaction of **5** with azidotrimethylsilane **g** did not yield the intended target triazole **16**, both **32a** and **32b** are novel compounds that retain the cyclic sulfonamide functional group of **1**.

**Scheme 4:**
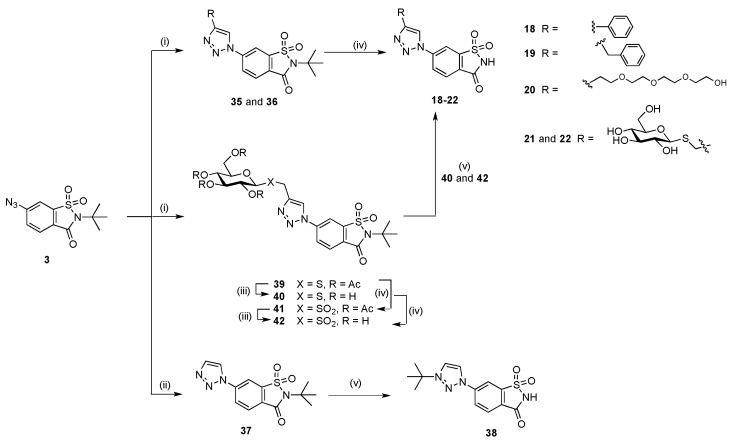
Synthesis of saccharin derivatives from *N*-*t*-butyl-protected 6-azidosaccharin **3**. Reagents and conditions: (i) **3** (1 equiv), alkyne (1 equiv), sodium ascorbate (0.4 equiv), CuSO_4_·5H_2_O (0.2 equiv), 1:1 *t*-BuOH:water, 45 °C–50 °C, 2 h—overnight; (ii) **3** (1 equiv), TMSC≡CH (2 equiv), sodium ascorbate (0.4 equiv), CuSO_4_·5H_2_O (0.2 equiv), 1:1 *t*-BuOH:water, 45 °C, overnight; (iii) 8% HCl in MeOH, r.t., 90 h; (iv) *m*CPBA, CH_2_Cl_2_, 0 °C to r.t.; (v) TFA, reflux, 18 h. The phenyl derivative **18** as previously reported [10].

The target compounds prepared from the reaction of azidosaccharin **3** [10] with alkynes **h**–**l** have similar physicochemical diversity to azides **a**–**g** reacted with the ethynylsaccharin **5** (Figure 3). We have previously reported the synthesis of the phenyl derivative **18** while all other compounds are novel [10]. Reaction of azide **3** with 3-phenyl-1-propyne (benzyl alkyne) **i** and PEG alkyne **j** [31] under standard CuAAC conditions gave triazoles **35** and **36**, respectively (Scheme 4). Acid mediated cleavage of the *t*-butyl protecting group in refluxing TFA furnished the target saccharin compounds **19** and **20**, respectively. CuAAC of ethynyltrimethylsilane (TMSC≡CH) **l** and azide **3** [10] gave **37**, a 1-substituted 1,2,3-triazole. Consistent with the outcome of deprotection of the related 1-substituted triazole **31**, treatment of **37** with TFA removed the *N*-*t*-butyl group from the sulfonamide nitrogen, but furnished the alternate N-alkylation product **38**, where the *t*-butyl group is on N-3 of the 1,2,3-triazole instead of the desired target compound **16** (Scheme 4). ^1^H-NMR and HRMS confirmed the formation of **38**. Finally, the reaction of saccharin azide **3** and propargyl 2,3,4,6-tetra-*O*-acetyl-thio-β-d-glucopyranoside **k′** [14] using CuAAC gave glycoconjugate **39** in high yield. Acidic cleavage of the acetyl groups of **39** (HCl in MeOH, 90 h) gave the free sugar derivative **40**. Given the long 90 h reaction time, triazole **40** was also prepared directly from **3** utilising propargyl thio-β-d-glucopyranoside **k** [32], as described for **14** and **15**. Oxidation of **39** and **40** with *m*-chloroperbenzoic acid (*m*CPBA) gave sulfones **41** and **42** in high yields, respectively. Compound **42** was also prepared by the deacetylation of **41** under acidic conditions, and this demonstrated the versatility of protecting group manipulation in the presence of the saccharin core scaffold. The *N*-*t*-butyl protecting group of **40** and **42** was removed by refluxing in TFA for 18 h to afford **21** and **22**, respectively (Scheme 4).

## 3. Materials and Methods

### 3.1. General Chemistry

All starting materials and reagents were purchased from commercial suppliers. All solvents were available commercially dried or dried prior to use. Reaction progress was monitored by thin layer chromatography (TLC) using silica gel-60 F254 plates (Merck Millipore, Darmstadt, Germany) with detection by short wave ultraviolet (UV) fluorescence (λ = 254 nm) and staining with 5% *w*/*v* dodecamolybdophosphoric acid in ethanol or vanillin staining (5 g of vanillin in a mixture of EtOH:H_2_O:H_2_SO_4_ = 85:10:2.75) with subsequent heating. Silica gel flash chromatography was performed using silica gel 60 Å (230–400 mesh) (Merck Millipore, Darmstadt, Germany). NMR (^1^H, ^13^C, ^19^F, gradient correlation spectroscopy (gCOSY), and heteronuclear single quantum coherence (HSQC) spectra were recorded on either a 400 or 500 MHz spectrometer at 30 °C. ^1^H-NMR spectra were obtained at 500 MHz and were referenced to the residual solvent peak (CDCl_3_ δ 7.26 ppm, dimethylsulfoxide (DMSO)-*d*_6_ δ 2.50 ppm). ^13^C-NMR spectra were recorded at 125 MHz and were referenced to the internal solvent (CDCl_3_ δ 77.0 ppm, DMSO-*d*_6_ δ 39.5 ppm). ^19^F-NMR spectra were recorded at 376 MHz. Multiplicity is indicated as follows: s (singlet); d (doublet); t (triplet); m (multiplet); dd (doublet of doublet); ddd (doublet of doublet of doublet); b (broad). Coupling constants are reported in hertz (Hz). Melting points are uncorrected. Low and high resolution mass spectra (MS) were recorded using electrospray ionization (ESI) in positive ion and/or negative ion modes as stated. All MS analysis samples were prepared as solutions in methanol. The purity of all compounds was ≥95% as determined by HPLC with UV. ^1^H-, ^13^C-, and ^19^F-NMR spectra of all novel compounds are provided in the supporting information.

*N*-*t*-Butyl-6-amino-1,2-benzisothiazole-3-one-1,1-dioxide **7**, *N*-*t*-butyl-6-azido-1,2-benzisothiazole-3-one-1,1-dioxide **3**, and phenyl derivative **18** were synthesised using methods we have previously reported [10]. Azide and alkyne building blocks that were not commercially available were prepared in accordance with the literature, including: azidobenzene **a** [22], benzylazide **b** [23], PEG azide **c** [24], 2,3,4,6-tetra-*O*-acetyl-β-d-glucopyranosyl azide **d′** [25], 2-deoxy-2-fluoro-β-d-glucopyranosyl azide **e** [28], 2-deoxy-2-fluoro-β-d-glycopyranosyl azide **f** [28], propargyl 2,3,4,6-tetra-*O*-acetyl-thio-β-d-glucopyranoside **k′** [14], propargyl thio-β-d-glucopyranoside **k** [32], and PEG alkyne **j** [31].

### 3.2. General Procedure 1—CuAAC

A mixture of azide (1.0 equiv) and alkyne (1.0 equiv) was prepared in *t*-butyl alcohol/H_2_O (1:1, 6–10 mL). To the mixture was added a solution of sodium ascorbate (0.4 equiv) in water (0.25 mL) followed by a solution of CuSO_4_.5H_2_O (0.2 equiv) in water (0.25 mL). The resulting suspension was stirred vigorously at the temperature and time indicated below. The solvent was removed in vacuo and the residue was purified by column chromatography on silica gel using the eluent conditions described below.

### 3.3. N-t-Butyl-6-ethynyl-1,2-benzisothiazole-3-one-1,1-dioxide *(**5**)*

TBAF (1.0 M in THF, 0.328 mL, 0.328 mmol) was added to a solution of *N-t*-butyl-6-trimethylsilylethynyl-1,2-benzisothiazole-3-one-1,1-dioxide (**9**) (0.100 g, 0.298 mmol) and acetic acid (0.051 mL, 0.894 mmol) in THF (5 mL). The reaction mixture was stirred for 5 min, then quenched by the addition of water (20 mL) and extracted into EtOAc (3 × 30 mL). The combined organic fractions were washed with brine (30 mL), dried with MgSO_4_, filtered, and the solvent was removed in vacuo. The residue was purified by column chromatography on silica gel (EtOAc/hexane = 1:9) to give the title compound **5** (0.076 g, 97%) as a pale yellow solid. m.p. 162–164 °C (EtOAc/hexane); ^1^H-NMR (500 MHz, CDCl_3_) δ 1.76 (s, 9H, *t*Bu), 3.40 (s, 1H, CH_alkyne_), 7.83 (dd, *J* = 1.3, 7.9 Hz, 1H, Ar-H), 7.90 (dd, *J* = 0.7, 1.4 Hz, 1H, Ar-H), 7.93 (dd, *J* = 0.7, 7.9 Hz, 1H, Ar-H); ^13^C-NMR (125 MHz, CDCl_3_) δ 27.9 (C(CH_3_)_3_), 61.6 (C(CH_3_)_3_), 80.9 (PhCCH), 83.1 (PhCCH), 123.6 (Ar-CH), 124.6 (Ar-CH), 126.8 (Ar-C), 129.1 (Ar-C), 137.5 (Ar-CH), 138.2 (Ar-C), 159.4 (C=O); HRMS-ESI [M + Na]^+^ Calcd. for C_13_H_13_NNaO_3_S: 286.0508. Found: 286.0529.

### 3.4. N-t-Butyl-6-N,N-bis(prop-2-yn-1-yl)amino-1,2-benzisothiazole-3-one-1,1-dioxide *(**6**)*

To a solution of *N*-*t*-butyl-6-amino-1,2-benzisothiazole-3-one-1,1-dioxide **7** [10] (0.100 g, 0.393 mmol) in DMF (5 mL) was added cesium carbonate (0.256 g, 0.786 mmol) and the solution was cooled to 0 °C. Propargyl bromide 80% solution in toluene (0.096 mL, 0.865 mmol) was added dropwise and the solution was left to stir for 48 h. The solvent was removed in vacuo. The residue was dissolved into EtOAc (50 mL) and washed with water (3 × 40 mL). The combined organic fractions were washed with brine (50 mL), dried (MgSO_4_), and the solvent was removed in vacuo. The residue was purified by column chromatography on silica gel (EtOAc/hexane = 1:9 to 1:2) to give the title compound **6** (0.098 g, 75%) as a white solid. m.p. 178–180 °C (EtOAc/hexane); ^1^H-NMR (500 MHz, (CD_3_)_2_SO) δ 1.37 (s, 9H, *t*Bu), 3.27 (t, *J* = 2.3 Hz, 2H, CH_2_CCH), 4.43 (d, *J* = 2.5 Hz, 4H, NCH_2_), 7.29 (dd, *J* = 2.4, 8.8 Hz, 1H, Ar-H), 7.42 (d, *J* = 2.3 Hz, 1H, Ar-H), 7.85 (d, *J* = 8.7 Hz, 1H, Ar-H); ^13^C-NMR (125 MHz, (CD_3_)_2_SO) δ 27.5 (C(CH_3_)_3_), 40.3 (NCH_2_), 59.8 (C(CH_3_)_3_), 75.6 (CH_2_CCH), 78.7 (CH_2_CCH), 103.7 (Ar-CH), 114.2 (Ar-C), 118.6, 125.5 (Ar-CH), 139.4, 151.9 (Ar-C), 159.8 (C=O); HRMS-ESI [M + Na]^+^ Calcd. for C_17_H_18_N_2_NaO_3_S: 353.0930. Found: 353.0944.

### 3.5. N-t-Butyl-6-iodo-1,2-benzisothiazole-3-one-1,1-dioxide *(**8**)*

To a solution of *p*-toluenesulfonic acid, *p*TsOH·H_2_O (4.49 g, 23.6 mmol) in CH_3_CN (20 mL) was added *N*-*t*-butyl-6-amino-1,2-benzisothiazole-3-one-1,1-dioxide (**7**) [10] (2.00 g, 7.86 mmol). The resulting suspension of amine salt was cooled to 10–15 °C and to this was added, dropwise, a solution of NaNO_2_ (1.09 g, 15.7 mmol) and KI (3.26 g 19.7 mmol) in H_2_O (5 mL). The reaction mixture was stirred for 10 min, then warmed to r.t. and stirred for 1 h. To the reaction mixture was added H_2_O (10 mL), NaHCO_3_ (1.0 M; until pH = 9–10) and Na_2_S_2_O_3_ (2.0 M, 5 mL). The solution was extracted with EtOAc (3 × 50 mL) and the combined organic extracts were washed with brine (100 mL), dried (MgSO_4_), and the solvent was removed in vacuo. The residue was purified by column chromatography on silica gel (EtOAc/hexane = 1:2) to give the title compound **8** (2.39 g, 83%) as a light brown solid. m.p. 166–168 °C (EtOAc/hexane); ^1^H-NMR (500 MHz, CDCl_3_) δ 1.75 (s, 9H, *t*Bu), 7.69 (dd, *J* = 0.6, 8.0 Hz, 1H, Ar-H), 8.11 (dd, *J* = 1.4, 8.0 Hz, 1H, Ar-H), 8.16 (dd, *J* = 0.5, 1.4 Hz, 1H, Ar-H); ^13^C-NMR (125 MHz, CDCl_3_) δ 27.8 (C(CH_3_)_3_), 61.6 (C(CH_3_)_3_), 101.1 (Ar-C), 125.8 (Ar-CH), 126.8 (Ar-C), 129.0 (Ar-CH), 139.0 (Ar-C), 143.3 (Ar-CH), 159.6 (C=O); HRMS-ESI [M + Na]^+^ Calcd. for C_11_H_12_INNaO_3_S: 387.9475. Found: 387.951.

### 3.6. N-t-Butyl-6-trimethylsilylethynyl-1,2-benzisothiazole-3-one-1,1-dioxide *(**9**)*

*N*-*t*-Butyl-6-iodo-1,2-benzisothiazole-3-one-1,1-dioxide (**8**) (1.00 g, 2.74 mmol), Pd(PPh_3_)Cl_2_ (0.077 g, 0.110 mmol) and CuI (0.026 g, 0.137 mmol) were dried together under high vacuum and then flushed with argon. Triethylamine (20 mL) was added and the reaction mixture was stirred and heated to 40 °C. To this was added ethynyltrimethylsilane **l** (0.468 mL, 3.29 mmol) and the solution was stirred for 2 h. The reaction mixture was filtered through a pad of Celite and washed with EtOAc (100 mL). The residue was purified by column chromatography on silica gel (EtOAc/hexane = 1:19 to 1:9) to give the title compound **9** (0.871 g, 95%) as a light brown solid. m.p. 114–115 °C (EtOAc/hexane); ^1^H-NMR (500 MHz, CDCl_3_) δ 0.27 (s, 9H, Si(CH_3_)_3_), 1.76 (s, 9H, *t*Bu), 7.78 (dd, *J* = 1.3, 7.9 Hz, 1H, Ar-H), 7.87 (dd, *J* = 0.7, 1.3 Hz, 1H, Ar-H), 7.90 (dd, *J* = 0.7, 7.9 Hz, 1H, Ar-H); ^13^C-NMR (125 MHz, CDCl_3_) δ −0.3 (Si(CH_3_)_3_), 27.9 (C(CH_3_)_3_), 61.5 (C(CH_3_)_3_), 101.7 (Si(CH_3_)_3_C), 101.8 (Si(CH_3_)_3_CC), 123.4 (Ar-CH), 124.5 (Ar-CH), 126.3 (Ar-C), 130.2 (Ar-C), 137.2 (Ar-CH), 138.1 (Ar-C), 159.6 (C=O); HRMS-ESI [M + Na]^+^ Calcd. for C_16_H_21_NNaO_3_SSi: 358.0904. Found: 358.0899.

### 3.7. 6-(1-Phenyl-1H-1,2,3-triazol-4-yl)-1,2-benzisothiazole-3-one-1,1-dioxide *(**10**)*

*N*-*t*-Butyl protected **24** (0.090 g, 0.235 mmol) was refluxed in TFA (3 mL) for 18 h. The solvent was removed in vacuo. EtOAc was added to the residue and the solid was collected by filtration to give the title compound **10** (0.065 g, 85%) as a white solid. m.p. greater than 300 °C (EtOAc); ^1^H-NMR (500 MHz, (CD_3_)_2_SO) δ 7.55 (t, *J* = 7.4 Hz, 1H, Ar-H), 7.66 (t, *J* = 7.9 Hz, 2H, Ar-H), 7.94 (d, *J* = 7.6 Hz, 2H, Ar-H), 8.13 (d, *J* = 8.0 Hz, 1H, Ar-H), 8.50 (dd, *J* = 1.4, 8.0 Hz, 1H, Ar-H), 8.60 (d, *J* = 1.4 Hz, 1H, Ar-H), 9.64 (s, 1H, CH_triazole_); ^13^C-NMR (125 MHz, (CD_3_)_2_SO) δ 117.2, 120.1 (Ar-CH), 122.2 (CH_triazole_), 125.8 (Ar-CH), 126.8 (Ar-C), 129.1, 130.1, 130.5 (Ar-CH), 136.3, 137.0, 140.7 (Ar-C), 145.0 (C_triazole_), 160.7 (C=O); HRMS-ESI [M − H]^−^ Calcd. for C_15_H_9_N_4_O_3_S: 325.0389. Found: 325.0378.

### 3.8. 6-(1-Benzyl-1H-1,2,3-triazol-4-yl)-1,2-benzisothiazole-3-one-1,1-dioxide *(**11**)*

*N*-*t*-Butyl protected **25** (0.080 g, 0.202 mmol) was refluxed in TFA (3 mL) for 18 h. The solvent was removed in vacuo and the residue was purified by column chromatography on silica gel (MeOH/CH_2_Cl_2_ = 1:4) and gave the title compound **11** (0.068 g, 99%) as a white solid. m.p. 247–249 °C (MeOH/CH_2_Cl_2_); ^1^H-NMR (500 MHz, (CD_3_)_2_SO) δ 5.67 (s, 2H, CH_2_), 7.33–7.43 (m, 5H, Ar-H), 7.66 (d, *J* = 8.2 Hz, 1H, Ar-H), 8.09–8.12 (m, 2H, Ar-H), 8.87 (s, 1H, CH_triazole_); ^13^C-NMR (125 MHz, (CD_3_)_2_SO) δ 53.2 (CH_2_), 115.7, (Ar-CH), 122.9 (CH_triazole_), 123.3, 128.0, 128.2, 128.8 (Ar-CH), 133.5, 133.6, 135.7, 145.5 (Ar-C), 146.0 (C_triazole_), 167.1 (C=O); HRMS-ESI [M − H]^−^ Calcd. for C_16_H_11_N_4_O_3_S: 339.0546. Found: 339.0534.

### 3.9. 6-(1-[2-[2-[2-(2-Hydroxyethoxy)ethoxy]ethoxy]ethyl]-1H-1,2,3-triazol-4-yl)-1,2-benzisothiazole-3-one-1,1-dioxide *(**12**)*

*N*-*t*-Butyl protected **26** (0.087 g, 0.180 mmol) was refluxed in TFA (3 mL) for 18 h. The solvent was removed in vacuo and the residue purified by reverse phase (RP-18) column chromatography (MeOH/H_2_O = 0:1 to 1:9, product eluting at 1:9) to give the title compound **12** (0.074 g, 96%) as a colourless oil which solidified to a white gum upon standing. ^1^H-NMR (500 MHz, (CD_3_)_2_SO) δ 3.34–3.37 (m, 2H, CH_2_), 3.42–3.52 (m, 10H, CH_2_), 3.54–3.57 (m, 2H, CH_2_), 3.88 (dd, *J* = 4.6, 5.6 Hz, 2H, CH_2_), 4.61 (t, *J* = 5.1 Hz, 1H, OH), 7.94 (d, *J* = 7.9 Hz, 1H, Ar-H), 8.32 (dd, *J* = 1.4, 7.9 Hz, 1H, Ar-H), 8.40 (d, *J* = 1.4 Hz, 1H, Ar-H), 8.86 (s, 1H, CH_triazole_); ^13^C-NMR (125 MHz, (CD_3_)_2_SO) δ 49.9, 60.2, 68.5, 69.60, 69.64, 69.7, 69.8, 72.3 (CH_2_), 116.7, (Ar-CH), 124.0 (CH_triazole_), 125.2 (Ar-CH), 128.0 (Ar-C), 129.9 (Ar-CH), 136.7, 141.9 (Ar-C), 144.2 (C_triazole_), 162.2 (C=O); HRMS-ESI [M + Na]^+^ Calcd. for C_17_H_22_N_4_NaO_7_S: 425.1136. Found: 425.1132.

### 3.10. 6-(1-β-d-Glucopyranosyl-1H-1,2,3-triazol-4-yl)-1,2-benzisothiazole-3-one-1,1-dioxide *(**13**)*

*N*-*t*-Butyl protected **28** (0.060 g, 0.128 mmol) was refluxed in TFA (1.5 mL) for 18 h. The solvent was removed in vacuo and the residue was purified by RP-18 column chromatography (MeOH/H_2_O = 0:1 to 1:9, product eluting at 1:19) to give the title compound **13** (0.050 g, 94%) as a white solid. m.p. 227–229 °C (MeOH/H_2_O); ^1^H-NMR (500 MHz, (CD_3_)_2_SO) δ 3.26 (t, *J* = 9.2 Hz, 1H, H-4), 3.42–3.49 (m, 2H, H-3, H-6), 3.50–3.55 (m, 1H, H-5), 3.71–3.78 (m, 2H, H-2, H-6), 5.64 (d, *J* = 9.2 Hz, 1H, H-1), 8.08 (d, *J* = 8.0 Hz, 1H, Ar-H), 8.45 (dd, *J* = 1.4, 8.0 Hz, 1H, Ar-H), 8.57 (d, *J* = 1.3 Hz, 1H, Ar-H), 9.23 (s, 1H, CH_triazole_); ^13^C-NMR (125 MHz, (CD_3_)_2_SO) δ 60.7 (C-6), 69.6 (C-4), 72.5 (C-2), 76.6 (C-3), 80.0 (C-5), 87.8 (C-1), 117.0, (Ar-CH), 123.0 (CH_triazole_), 125.7 (Ar-CH), 126.9 (Ar-C), 130.3 (Ar-CH), 137.2, 140.9 (Ar-C), 144.2 (C_triazole_), 161.0 (C=O); HRMS-ESI [M − H]^−^ Calcd. for C_15_H_15_N_4_O_8_S: 411.0616. Found: 411.0601.

### 3.11. 6-(1-[2-Deoxy-2-fluoro-β-d-glucopyranosyl]-1H-1,2,3-triazol-4-yl)-1,2-benzisothiazole-3-one-1,1-dioxide *(**14**)*

*N-t*-Butyl protected **29** (0.080 g, 0.170 mmol) was refluxed in TFA (1.5 mL) for 18 h. The solvent was removed in vacuo and the residue purified by RP-18 column chromatography (MeOH/H_2_O = 0:1 to 1:9, product eluting at 1:9) to give the title compound **14** (0.057 g, 81%) as a white solid. m.p. 274–276 °C (MeOH/H_2_O); ^1^H-NMR (500 MHz, (CD_3_)_2_SO) δ 3.35 (dd, *J* = 8.9, 9.8 Hz, 1H, H-4), 3.49 (dd, *J* = 5.7, 12.2 Hz, 1H, H-6), 3.67 (ddd, *J* = 2.0, 5.8, 9.9 Hz, 1H, H-5), 3.73 (dd, *J* = 2.0, 12.3 Hz, 1H, H-6), 3.82 (dt, *J* = 8.9, 15.6 Hz, 1H, H-3), 4.80 (dt, *J* = 9.0, 51.0 Hz, 1H, H-2), 6.20 (dd, *J* = 2.4, 9.1 Hz, 1H, H-1), 8.09 (d, *J* = 8.1 Hz, 1H, Ar-H), 8.43 (dd, *J* = 1.4, 8.1 Hz, 1H, Ar-H), 8.55 (d, *J* = 1.4 Hz, 1H, Ar-H), 9.35 (s, 1H, CH_triazole_); ^13^C-NMR (125 MHz, (CD_3_)_2_SO) δ 60.3 (C-6), 69.3 (d, *J* = 8.0 Hz, C-4), 74.1 (d, *J* = 15.9 Hz, C-3), 79.9 (C-5), 84.2 (d, *J* = 24.2 Hz, C-1), 91.2 (d, *J* = 186.8 Hz, C-2), 117.2 (Ar-H), 123.1 (CH_triazole_), 125.7 (Ar-CH), 127.1 (Ar-C), 130.1 (Ar-CH), 136.7, 140.1 (Ar-C), 144.7 (C_triazole_), 160 (C=O); ^19^F-NMR (376 MHz, (CD_3_)_2_SO) δ -193.6 (ddd, *J* = 2.4, 15.6, 51.1 Hz); HRMS-ESI [M − H]^−^ Calcd. for C_15_H_14_FN_4_O_7_S: 413.0573. Found: 413.0563.

### 3.12. 6-(1-β-d-Galactopyranosyl-1H-1,2,3-triazol-4-yl)-1,2-benzisothiazole-3-one-1,1-dioxide *(**15**)*

*N*-*t*-Butyl protected **30** (0.080 g, 0.171 mmol) was refluxed in TFA (1.5 mL) for 18 h. The solvent was removed in vacuo and the residue was purified by RP-18 column chromatography (MeOH/H_2_O = 0:1 to 1:19, product eluting at 1:32) to give the title compound **15** (0.024 g, 34%) as a white solid. m.p. 232–235 °C (MeOH/H_2_O); ^1^H-NMR (500 MHz, (CD_3_)_2_SO) δ 3.50–3.58 (m, 2H, 6-CH_2_), 3.60 (dd, *J* = 3.1, 9.4 Hz, 1H, H-3), 3.76–3.82 (m, 2H, H-4, H-5), 4.09 (t, *J* = 9.2 Hz, 1H, H-2), 5.57 (d, *J* = 9.1 Hz, 1H, H-1), 7.95 (d, *J* = 8.0 Hz, 1H, Ar-H), 8.30 (dd, *J* = 1.5, 7.9 Hz, 1H, Ar-H), 8.49 (d, *J* = 1.4 Hz, 1H, Ar-H), 9.16 (s, 1H, CH_triazole_); ^13^C-NMR (125 MHz, (CD_3_)_2_SO) δ 60.5 (C-6), 68.4 (C-4), 69.4 (C-2), 73.5 (C-3), 78.5 (C-5), 88.4 (C-1), 116.7 (Ar-CH), 122.5 (CH_triazole_), 124.9 (Ar-CH), 128.9 (Ar-C), 129.7 (Ar-CH), 136.1, 142.5 (Ar-C), 144.6 (C_triazole_), 162.9 (C=O); HRMS-ESI [M − H]^−^ Calcd. for C_15_H_15_N_4_O_8_S: 411.0616. Found: 411.0608.

### 3.13. 6-*N*,*N*-Bis([1-β-d-glucopyranosyl-1H-1,2,3-triazol-4-yl]methyl)amino-1,2-benzisothiazole-3-one-1,1-dioxide *(**17**)*

*N*-*t*-Butyl protected **34** (0.070 g, 0.095 mmol) was refluxed in TFA (2.0 mL) for 18 h. The solvent was removed in vacuo and the residue was purified by RP-18 column chromatography (MeOH/H_2_O = 0:1 to 1:9, product eluting at 1:9) to give the title compound **17** (0.057 g, 84%) as a white solid. m.p. 184–186 °C (MeOH/H_2_O); ^1^H-NMR (500 MHz, (CD_3_)_2_SO) δ 3.22 (t, *J* = 9.0 Hz, 2H, H-4), 3.38 (t, *J* = 8.9 Hz, 2H, H-3), 3.41–3.47 (m, 4H, H-5, H-6), 3.66–3.71 (m, 2H, H-6), 3.75 (t, *J* = 9.1 Hz, 2H, H-2), 4.48–4.72 (m, 2H, OH), 4.80–4.89 (m, 4H, NCH_2_), 5.03–5.46 (m, 6H, OH), 5.53 (d, *J* = 9.3 Hz, 2H, H-1), 7.30 (dd, *J* = 2.4, 8.9 Hz, 1H, Ar-H), 7.54 (d, *J* = 2.3 Hz, 1H, Ar-H), 7.68 (d, *J* = 8.8 Hz, 1H, Ar-H), 8.38 (s, 2H, CH_triazole_); ^13^C-NMR (125 MHz, (CD_3_)_2_SO) δ 45.2 (NCH_2_), 60.7 (C-6), 69.6 (C-4), 72.1 (C-2), 76.9 (C-3), 80.0 (C-5), 87.5 (C-1), 102.8, (Ar-CH), 114.6 (Ar-C), 116.6 (Ar-C), 122.6 (CH_triazole_), 125.7 (Ar-CH), 142.4 (Ar-C), 142.8 (C_triazole_), 152.8 (Ar-C), 161.4 (C=O); HRMS-ESI [M + Na]^+^ Calcd. for C_25_H_32_N_8_NaO_13_S: 707.1702. Found: 707.1756.

### 3.14. 6-(4-Benzyl-1H-1,2,3-triazol-1-yl)-1,2-benzisothiazole-3-one-1,1-dioxide *(**19**)*

*N*-*t*-Butyl protected **35** (0.080 g, 0.202 mmol) was refluxed in TFA (3 mL) for 18 h. The solvent was removed in vacuo and the residue was purified by column chromatography on silica gel (MeOH/CH_2_Cl_2_ = 1:9 to 1:4) to give the title compound **19** (0.044 g, 64%) as a light brown solid. m.p. 177–179 °C (MeOH/CH_2_Cl_2_); ^1^H-NMR (500 MHz, (CD_3_)_2_SO) δ 4.12 (s, 2H, CH_2_), 7.21–7.26 (m, 1H, Ar-H), 7.31–7.34 (m, 4H, Ar-H), 8.10 (d, *J* = 8.3 Hz, 1H, Ar-H), 8.43 (dd, *J* = 1.9, 8.4 Hz, 1H, Ar-H), 8.65 (d, *J* = 1.9 Hz, 1H, Ar-H), 8.86 (s, 1H, CH_triazole_); ^13^C-NMR (125 MHz, (CD_3_)_2_SO) δ 31.2 (CH_2_) 111.9, (Ar-CH), 121.5 (CH_triazole_), 124.8, 126.3, 126.4 (Ar-CH), 128.0 (Ar-C), 128.5, 128.6 (Ar-CH), 138.8, 140.8, 142.3 (Ar-C), 147.8 (C_triazole_), 161.4 (C=O); HRMS-ESI [M − H]^−^ Calcd. for C_16_H_11_N_4_O_3_S: 339.0546. Found: 339.0532.

### 3.15. 6-[4-(13-Hydroxy-2,5,8,11-tetraoxatridec-1-yl)-1H-1,2,3-triazol-1-yl]-1,2-benzisothiazole-3-one-1,1-dioxide *(**20**)*

*N*-*t*-Butyl protected **36** (0.080 g, 0.156 mmol) was refluxed in TFA (3 mL) for 18 h. The solvent was removed in vacuo and the residue was purified by RP-18 column chromatography (MeOH/H_2_O = 0:1 to 1:9, product eluting at 1:9) to give the title compound **20** (0.052 g, 73%) as a colourless oil. ^1^H-NMR (500 MHz, (CD_3_)_2_SO) δ 3.38–3.41 (m, 2H, CH_2_), 3.45–3.53 (m, 10H, CH_2_), 3.55–3.58 (m, 2H, CH_2_), 3.61–3.64 (m, 2H, CH_2_), 4.58 (t, *J* = 5.5 Hz, 1H, OH), 4.63 (s, 2H, CH_2_), 7.78 (d, *J* = 8.1 Hz, 1H, Ar-H), 8.18 (dd, *J* = 1.9, 8.1 Hz, 1H, Ar-H), 8.23 (d, *J* = 1.9 Hz, 1H, Ar-H), 8.98 (s, 1H, CH_triazole_); ^13^C-NMR (125 MHz, (CD_3_)_2_SO) δ 60.2, 63.3, 69.1, 69.7, 69.74, 69.79, 69.8, 72.3 (CH_2_), 110.8, (Ar-CH), 122.7 (CH_triazole_), 123.3, 124.2 (Ar-CH), 134.3, 138.5, 145.4 (Ar-C), 147.1 (C_triazole_), 166.7 (C=O); HRMS-ESI [M + Na]^+^ Calcd. for C_18_H_24_N_4_NaO_8_S: 479.1207. Found: 479.1202.

### 3.16. 6-(4-{[β-d-Glucopyranosyl]thiomethyl}-1H-1,2,3-triazol-1-yl)-1,2-benzisothiazole-3-one-1,1-dioxide *(**21**)*

*N*-*t*-Butyl protected **40** (0.100 g, 0.194 mmol) was refluxed in TFA (1.5 mL) for 18 h. The solvent was removed in vacuo and the residue was purified by RP-18 column chromatography (MeOH/H_2_O = 0:1 to 1:9, product eluting at 1:9) to give the title compound **21** (0.057 g, 64%) as a white solid. m.p. 176–178 °C (MeOH/H_2_O); ^1^H-NMR (500 MHz, (CD_3_)_2_SO) δ 3.03–3.10 (m, 2H, H-2, H-4), 3.16 (t, *J* = 8.6 Hz, 1H, H-3), 3.20 (ddd, *J* = 2.0, 6.4, 9.8 Hz, 1H, H-5), 3.46 (dd, *J* = 6.4, 11.9 Hz, 1H, H-6), 3.73 (dd, *J* = 2.0, 11.9 Hz, 1H, H-6), 3.95 (d, *J* = 14.4 Hz, 1H, SCH_2_), 4.09 (d, *J* = 14.4 Hz, 1H, SCH_2_), 4.34 (d, *J* = 9.6 Hz, 1H, H-1), 7.99 (d, *J* = 8.3 Hz, 1H, Ar-H), 8.31 (dd, *J* = 1.9, 8.3 Hz, 1H, Ar-H), 8.47 (d, *J* = 1.8 Hz, 1H, Ar-H), 8.92 (s, 1H, CH_triazole_); ^13^C-NMR (125 MHz, (CD_3_)_2_SO) δ 22.9 (SCH_2_), 61.3 (C-6), 70.1 (C-4), 73.2 (C-2), 78.1 (C-3), 81.0 (C-5), 84.0 (C-1), 111.5, (Ar-CH), 122.0 (CH_triazole_), 124.3, 125.6 (Ar-CH), 130.3, 140.0, 143.9 (Ar-C), 146.2 (C_triazole_), 163.2 (C=O); HRMS-ESI [M + H]^+^ Calcd. for C_16_H_19_N_4_O_8_S_2_: 459.0638. Found: 459.0665.

### 3.17. 6-(4-{[β-d-Glucopyranosyl]sulfonylmethyl}-1H-1,2,3-triazol-1-yl)-1,2-benzisothiazole-3-one-1,1-dioxide *(**22**)*

*N*-*t*-Butyl protected **42** (0.120 g, 0.220 mmol) was refluxed in TFA (1.5 mL) for 18 h. The solvent was removed in vacuo and the residue was purified by RP-18 column chromatography (MeOH/H_2_O = 0:1 to 1:9, product eluting at 5:95) to give the title compound **22** (0.046 g, 43%) as a white solid. m.p. 186–189 °C (MeOH/H_2_O); ^1^H-NMR (500 MHz, (CD_3_)_2_SO) δ 3.08 (dd, *J* = 8.8, 9.8 Hz, 1H, H-4), 3.29 (t, *J* = 8.8 Hz, 1H, H-3), 3.43 (ddd, *J* = 1.9, 6.8, 9.9 Hz, 1H, H-5), 3.51 (dd, *J* = 6.8, 12.1 Hz, 1H, H-6), 3.59 (t, *J* = 9.1 Hz, 1H, H-2), 3.80 (dd, *J* = 1.8, 12.2 Hz, 1H, H-6), 4.51 (d, *J* = 9.5 Hz, 1H, H-1), 4.70 (d, *J* = 14.7 Hz, 1H, SCH_2_), 4.85 (d, *J* = 14.7 Hz, 1H, SCH_2_), 8.14 (d, *J* = 8.3 Hz, 1H, Ar-H), 8.42 (dd, *J* = 1.9, 8.4 Hz, 1H, Ar-H), 8.65 (d, *J* = 1.8 Hz, 1H, Ar-H), 9.15 (s, 1H, CH_triazole_); ^13^C-NMR (125 MHz, (CD_3_)_2_SO) δ 48.3 (SCH_2_), 61.1 (C-6), 69.2 (C-2), 69.5 (C-4), 77.5 (C-3), 81.6 (C-5), 88.6 (C-1), 112.4, (Ar-CH), 124.7 (CH_triazole_), 125.3, 126.4 (Ar-CH), 128.4, 136.9, 140.7 (Ar-C), 142.2 (C_triazole_), 161.3 (C=O); HRMS-ESI [M + Na]^+^ Calcd. for C_16_H_18_N_4_NaO_10_S_2_: 513.0356. Found: 513.0404.

### 3.18. N-t-Butyl-6-(1-phenyl-1H-1,2,3-triazol-4-yl)-1,2-benzisothiazole-3-one-1,1-dioxide *(**24**)*

The title compound **24** was prepared from *N-t*-butyl-6-ethynyl-1,2-benzisothiazole-3-one-1,1-dioxide **5** (0.150 g, 0.570 mmol) and azidobenzene **a** [22] (0.068 g, 0.570 mmol) at 45 °C in 2 h according to general procedure 1. Purification of the crude product by flash chromatography (EtOAc/hexane = 9:1 to 1:1) gave the title compound **24** (0.162 g, 74%) as a yellow solid. m.p. 207–208 °C (EtOAc/hexane); ^1^H-NMR (500 MHz, (CD_3_)_2_SO) δ1.71 (s, 9H, *t*Bu), 7.53–7.56 (m, 1H, Ar-H), 7.64–7.68 (m, 2H, Ar-H), 7.91–7.95 (m, 2H, Ar-H), 8.15 (d, *J* = 8.0 Hz, 1H, Ar-H), 8.51 (dd, *J* = 1.4, 8.0 Hz, 1H, Ar-H), 8.61 (d, *J* = 1.4 Hz, 1H, Ar-H), 9.65 (s, 1H, CH_triazole_); ^13^C-NMR (125 MHz, (CD_3_)_2_SO) δ 27.3 (C(CH_3_)_3_), 60.5 (C(CH_3_)_3_), 116.7, 120.0 (Ar-CH), 122.3 (CH_triazole_), 125.1 (Ar-C), 125.7, 129.1, 130.0, 130.8 (Ar-CH), 136.3, 137.2, 138.1 (Ar-C), 144.8 (C_triazole_), 159.2 (C=O); HRMS-ESI [M + Na]^+^ Calcd. for C_19_H_18_N_4_NaO_3_S: 405.0992. Found: 405.0993.

### 3.19. N-t-Butyl-6-(1-benzyl-1H-1,2,3-triazol-4-yl)-1,2-benzisothiazole-3-one-1,1-dioxide *(**25**)*

The title compound **25** was prepared from *N-t*-butyl-6-ethynyl-1,2-benzisothiazole-3-one-1,1-dioxide **5** (0.099 g, 0.376 mmol) and benzylazide **b** [23] (0.050 g, 0.376 mmol) at 45 °C in 2 h according to general procedure 1. Purification of the crude product by flash chromatography (EtOAc/hexane = 9:1 to 1:2) gave the title compound **25** (0.144 g, 97%) as a white solid. m.p. 156–157 °C (EtOAc/hexane); ^1^H-NMR (500 MHz, (CD_3_)_2_SO) δ1.69 (s, 9H, *t*Bu), 5.70 (s, 2H, CH_2_), 7.33–7.44 (m, 5H, Ar-H), 8.09 (d, *J* = 8.1 Hz, 1H, Ar-H), 8.46 (dd, *J* = 1.4, 8.1 Hz, 1H, Ar-H), 8.58 (d, *J* = 1.4 Hz, 1H, Ar-H), 8.98 (s, 1H, CH_triazole_); ^13^C-NMR (125 MHz, (CD_3_)_2_SO) δ 27.3 (C(CH_3_)_3_), 53.3 (CH_2_), 60.5 (C(CH_3_)_3_), 116.6, (Ar-CH), 124.1 (CH_triazole_), 124.9 (Ar-C), 125.5, 128.1, 128.3, 128.9, 130.7 (Ar-CH), 135.5, 137.6, 138.1 (Ar-C), 144.3 (C_triazole_), 159.3 (C=O); HRMS-ESI [M + Na]^+^ Calcd. for C_20_H_20_N_4_NaO_3_S: 419.1148. Found: 419.1141.

### 3.20. N-t-Butyl-6-(1-[2-[2-[2-(2-hydroxyethoxy)ethoxy]ethoxy]ethyl]-1H-1,2,3-triazol-4-yl)-1,2-benzisothiazole-3-one-1,1-dioxide *(**26**)*

The title compound **26** was prepared from *N-t*-butyl-6-ethynyl-1,2-benzisothiazole-3-one-1,1-dioxide **5** (0.100 g, 0.380 mmol) and PEG azide **c** [24] (0.083 g, 0.380 mmol) at 45 °C in 16 h according to general procedure 1. Purification of the crude product by flash chromatography (MeOH/EtOAc = 0:1 to 5:95) gave the title compound **26** (0.167 g, 91%) as a pale-yellow oil. ^1^H-NMR (500 MHz, (CD_3_)_2_SO) δ 1.71 (s, 9H, *t*Bu), 3.34–3.37 (m, 2H, CH_2_), 3.41–3.48 (m, 6H, CH_2_), 3.48–3.52 (m, 2H, CH_2_), 3.54–3.57 (m, 2H, CH_2_), 3.88 (dd, *J* = 4.5, 5.6 Hz, 2H, CH_2_), 4.53 (t, *J* = 5.1 Hz, 1H, OH), 4.63 (t, *J* = 5.1 Hz, 2H, CH_2_), 8.11 (d, *J* = 8.1 Hz, 1H, Ar-H), 8.46 (dd, *J* = 1.4, 8.1 Hz, 1H, Ar-H), 8.58 (d, *J* = 1.4 Hz, 1H, Ar-H), 8.92 (s, 1H, CH_triazole_); ^13^C-NMR (125 MHz, (CD_3_)_2_SO) δ 27.3 (C(CH_3_)_3_), 50.0, 60.1 (CH_2_), 60.5 (C(CH_3_)_3_), 68.5, 69.60, 69.62, 69.69, 69.73, 72.3 (CH_2_), 116.5, (Ar-CH), 124.4 (CH_triazole_), 124.8 (Ar-C), 125.5, 130.7 (Ar-CH), 137.8, 138.1 (Ar-C), 143.8 (C_triazole_), 159.3 (C=O); HRMS-ESI [M + Na]^+^ Calcd. for C_21_H_30_N_4_NaO_7_S: 505.1727. Found: 505.1739.

### 3.21. N-t-Butyl-6-(1-[2,3,4,6-tetra-O-acetyl-β-d-glucopyranosyl]-1H-1,2,3-triazol-4-yl)-1,2-benzisothiazole-3-one-1,1-dioxide *(**27**)*

The title compound **27** was prepared from *N-t*-butyl-6-ethynyl-1,2-benzisothiazole-3-one-1,1-dioxide **5** (0.100 g, 0.380 mmol) and 2,3,4,6-tetra-*O*-acetyl-β-d-glucopyranosyl azide **d′** [25] (0.142 g, 0.380 mmol) at 50 °C in 2 h according to general procedure 1. Purification of the crude product by flash chromatography (EtOAc/hexane = 2:1 to 1:1) gave the title compound **27** (0.217 g, 90%) as a white solid. m.p. 112–115 °C (EtOAc/hexane); ^1^H-NMR (500 MHz, (CD_3_)_2_SO) δ 1.71 (s, 9H, *t*Bu), 1.83 (s, 3H, OCOCH_3_), 1.99 (s, 3H, OCOCH_3_), 2.02 (s, 3H, OCOCH_3_), 2.05 (s, 3H, OCOCH_3_), 4.11 (dd, *J* = 2.3, 12.6 Hz, 1H, H-6), 4.19 (dd, *J* = 5.4, 12.7 Hz, 1H, H-6), 4.46 (ddd, *J* = 2.3, 5.4 10.1 Hz, 1H, H-5), 5.16 (dd, *J* = 9.3, 10.1 Hz, 1H, H-4), 5.57 (t, *J* = 9.3 Hz, 1H, H-2), 5.64 (t, *J* = 9.5 Hz, 1H, H-3), 6.49 (d, *J* = 9.0 Hz, 1H, H-1), 8.14 (dd, *J* = 0.6, 8.0 Hz, 1H, Ar-H), 8.44 (dd, *J* = 1.5, 8.1 Hz, 1H, Ar-H), 8.58 (d, *J* = 1.3 Hz, 1H, Ar-H), 9.35 (s, 1H, CH_triazole_); ^13^C-NMR (125 MHz, (CD_3_)_2_SO) δ 19.9, 20.2, 20.4, 20.5 (OCOCH_3_), 27.3 (C(CH_3_)_3_), 60.6 (C(CH_3_)_3_), 61.7 (C-6), 67.5 (C-4), 70.4 (C-2), 71.9 (C-3), 73.4 (C-5), 84.0 (C-1), 116.7, (Ar-CH), 123.2 (CH_triazole_), 125.3 (Ar-C), 125.7, 131.0 (Ar-CH), 136.9, 138.1 (Ar-C), 144.6 (C_triazole_), 159.2 (C=O), 168.6, 169.3, 169.5, 170.0 (OCOCH_3_); HRMS-ESI [M + H]^+^ Calcd. for C_27_H_33_N_4_O_12_S: 637.1810. Found: 637.1811.

### 3.22. N-t-Butyl-6-(1-β-d-glucopyranosyl-1H-1,2,3-triazol-4-yl)-1,2-benzisothiazole-3-one-1,1-dioxide *(**28**)*

To a solution of **27** (0.149 g, 0.234 mmol) in methanol (9.2 mL) was added HCl (0.8 mL). The reaction was stirred at rt for 90 h and the solvent was removed in vacuo. The residue was purified by column chromatography on silica gel (MeOH/CH_2_Cl_2_ = 0:1 to 1:9) to give the title compound **28** (0.103 g, 94%) as a white solid. m.p. 169–170 °C (MeOH/CH_2_Cl_2_); ^1^H-NMR (500 MHz, (CD_3_)_2_SO) δ 1.70 (s, 9H, *t*Bu), 3.26 (t, *J* = 9.3 Hz, 1H, H-4), 3.45 (t, *J* = 8.9 Hz, 1H, H-3), 3.47–3.55 (m, 2H, H-5, H-6), 3.70–3.78 (m, 2H, H-2, H-6), 5.65 (d, *J* = 9.2 Hz, 1H, H-1), 8.13 (d, *J* = 8.1 Hz, 1H, Ar-H), 8.49 (dd, *J* = 1.4, 8.0 Hz, 1H, Ar-H), 8.61 (d, *J* = 1.3 Hz, 1H, Ar-H), 9.26 (s, 1H, CH_triazole_); ^13^C-NMR (125 MHz, (CD_3_)_2_SO) δ 27.3 (C(CH_3_)_3_), 60.5 (C(CH_3_)_3_), 60.7 (C-6), 69.6 (C-4), 72.5 (C-2), 76.6 (C-3), 80.0 (C-5), 87.8 (C-1), 116.6 (Ar-CH), 123.1 (CH_triazole_), 125.0 (Ar-C), 125.6, 130.7 (Ar-CH), 137.5, 138.1 (Ar-C), 144.1 (C_triazole_), 159.3 (C=O); HRMS-ESI [M + H]^+^ Calcd. for C_19_H_25_N_4_O_8_S: 469.1388. Found: 469.1397.

### 3.23. N-t-Butyl-6-(1-[2-deoxy-2-fluoro-β-d-glucopyranosyl]-1H-1,2,3-triazol-4-yl)-1,2-benzisothiazole-3-one-1,1-dioxide *(**29**)*

The title compound **29** was prepared from *N-t*-butyl-6-ethynyl-1,2-benzisothiazole-3-one-1,1-dioxide **5** (0.057 g, 0.217 mmol) and 2-deoxy-2-fluoro-β-d-glucopyranosyl azide **e** [28] (0.045 g, 0.217 mmol) at 50 °C in 2 h according to general procedure 1. Purification of the crude product by flash chromatography (MeOH/CH_2_Cl_2_ = 1:9) gave the title compound **29** (0.103 g, quant.) as a white solid. m.p. 142–144 °C (MeOH/CH_2_Cl_2_); ^1^H-NMR (500 MHz, (CD_3_)_2_SO) δ 1.71 (s, 9H, *t*Bu), 3.32–3.38 (m, 1H, H-4), 3.46–3.51 (m, 1H, H-6), 3.67 (ddd, *J* = 2.0, 5.7, 9.9 Hz, 1H, H-5), 3.73 (ddd, *J* = 2.0, 5.6, 12.1 Hz, 1H, H-6), 3.82 (dtd, *J* = 5.4, 8.9, 14.4 Hz, 1H, H-3), 4.72 (t, *J* = 5.8 Hz, 1H, OH-6), 4.79 (dt, *J* = 9.0, 51.0 Hz, 1H, H-2), 5.52 (d, *J* = 5.6 Hz, 1H, OH-4), 5.88 (d, *J* = 5.4 Hz, 1H, OH-3), 6.20 (dd, *J* = 2.4, 9.1 Hz, 1H, H-1), 8.14 (d, *J* = 8.1 Hz, 1H, Ar-H), 8.47 (dd, *J* = 1.5, 8.1 Hz, 1H, Ar-H), 8.59 (d, *J* = 1.4 Hz, 1H, Ar-H), 9.37 (s, 1H, CH_triazole_); ^13^C-NMR (125 MHz, (CD_3_)_2_SO) δ 27.3 (C(CH_3_)_3_), 60.3 (C-6), 60.5 (C(CH_3_)_3_), 69.3 (d, *J* = 8.0 Hz, C-4), 74.1 (d, *J* = 16.0 Hz, C-3), 79.9 (C-5), 84.2 (d, *J* = 24.3 Hz, C-1), 91.2 (d, *J* = 186.8 Hz, C-2), 116.7 (Ar-H), 123.2 (CH_triazole_), 125.3 (Ar-C), 125.7, 131.0 (Ar-CH), 137.1, 138.1, (Ar-C), 144.5 (C_triazole_), 159.2 (C=O); ^19^F-NMR (376 MHz, (CD_3_)_2_SO) δ -193.6 (ddd, *J* = 1.7, 15.6, 51.3 Hz); HRMS-ESI [M + Na]^+^ Calcd. for C_19_H_23_FN_4_NaO_7_S: 493.1164. Found: 493.1166.

### 3.24. N-t-Butyl-6-(1-β-d-galactopyranosyl-1H-1,2,3-triazol-4-yl)-1,2-benzisothiazole-3-one-1,1-dioxide *(**30**)*

The title compound **30** was prepared from *N*-*t*-butyl-6-ethynyl-1,2-benzisothiazole-3-one-1,1-dioxide **5** (0.103 g, 0.390 mmol) and β-d-galactopyranosyl azide **f** [25] (0.080 g, 0.390 mmol) at 50 °C in 2 h according to general procedure 1. Purification of the crude product by flash chromatography (MeOH/CH_2_Cl_2_ = 0:1 to 1:9) gave the title compound **30** (0.161 g, 88%) as a white solid. m.p. 195–196 °C (MeOH/CH_2_Cl_2_); ^1^H-NMR (500 MHz, (CD_3_)_2_SO) δ 1.70 (s, 9H, *t*Bu), 3.49–3.58 (m, 2H, 6-CH_2_), 3.58–3.63 (m, 1H, H-3), 3.77–3.83 (m, 2H, H-4, H-5), 4.08 (td, *J* = 5.8, 9.2, 9.3 Hz, 1H, H-2), 4.71 (t, *J* = 5.6 Hz, 1H, OH-6), 4.76 (d, *J* = 4.3 Hz, 1H, OH-4), 5.11 (d, *J* = 5.5 Hz, 1H, OH-3), 5.32 (d, *J* = 5.8 Hz, 1H, OH-2), 5.59 (d, *J* = 9.1 Hz, 1H, H-1), 8.11 (d, *J* = 8.1 Hz, 1H, Ar-H), 8.52 (dd, *J* = 1.5, 8.1 Hz, 1H, Ar-H), 8.67 (d, *J* = 1.3 Hz, 1H, Ar-H), 9.24 (s, 1H, CH_triazole_); ^13^C-NMR (125 MHz, (CD_3_)_2_SO) δ 27.3 (C(CH_3_)_3_), 60.5 (C(CH_3_)_3_ and C-6), 68.4 (C-4), 69.5 (C-2), 73.4 (C-3), 78.5 (C-5), 88.4 (C-1), 116.7 (Ar-CH), 123.0 (CH_triazole_), 125.0 (Ar-C), 125.5, 130.1 (Ar-CH), 137.6, 138.1 (Ar-C), 144.1 (C_triazole_), 159.3 (C=O); HRMS-ESI [M + H]^+^ Calcd. for C_19_H_25_N_4_O_8_S: 469.1388. Found: 469.1340.

### 3.25. N-t-Butyl-6-1H-1,2,3-triazol-4-yl-1,2-benzisothiazole-3-one-1,1-dioxide *(**31**)*

*N*-*t*-Butyl-6-ethynyl-1,2-benzisothiazole-3-one-1,1-dioxide **5** (0.150 g, 0.570 mmol) and azidotrimethylsilane **g** (0.151 mL, 1.14 mmol) were dissolved in *tert*-butyl alcohol/H_2_O (1:1, 8 mL). To the reaction mixture was added a solution of sodium ascorbate (0.045 g, 0.228 mmol) in water (0.25 mL) followed by a solution of CuSO_4_·5H_2_O (0.028 g, 0.114 mmol) in water (0.25 mL). The suspension was stirred vigorously at 45 °C overnight. The solvent was removed in vacuo and the residue was purified by column chromatography on silica gel (EtOAc/hexane = 2:3) to give the title compound **31** (0.086 g, 50%) as a white solid. m.p. greater than 300 °C (EtOAc/hexane); ^1^H-NMR (500 MHz, (CD_3_)_2_SO) δ 1.70 (s, 9H, *t*Bu), 8.10 (d, *J* = 8.1 Hz, 1H, Ar-H), 8.46 (dd, *J* = 1.4, 8.1 Hz, 1H, Ar-H), 8.62 (d, *J* = 1.4 Hz, 1H, Ar-H), 8.75 (s, 1H, CH_triazole_), 15.5 (brs, 1H, NH); ^13^C-NMR (125 MHz, (CD_3_)_2_SO) δ 27.3 (C(CH_3_)_3_), 60.5 (C(CH_3_)_3_), 116.9, (Ar-CH), 125.0 (Ar-C), 125.5 (Ar-CH), 127.3 (CH_triazole_), 131.1 (Ar-CH), 137.7, 138.1 (Ar-C), 143.5 (C_triazole_), 159.3 (C=O); HRMS-ESI [M − H]^−^ Calcd. for C_13_H_13_N_4_O_3_S: 305.0703. Found: 305.0690.

### 3.26. 6-(1-t-Butyl-1H-1,2,3-triazol-4-yl)-1,2-benzisothiazole-3-one-1,1-dioxide *(**32a**)* and 6-(2-*t*-Butyl-1H-1,2,3-triazol-4-yl)-1,2-benzisothiazole-3-one-1,1-dioxide *(**32b**)*

*N*-*t*-Butyl protected **31** (0.085 g, 0.277 mmol) was refluxed in TFA (3 mL) for 18 h. The solvent was removed in vacuo and the residue was purified by column chromatography on silica gel (MeOH/CH_2_Cl_2_ = 1:9) to give the title compounds **32a** and **32b** (0.068 g, 80%) as white solids. m.p. 203–204 °C (MeOH/CH_2_Cl_2_); ^1^H-NMR (500 MHz, (CD_3_)_2_SO) δ1.67 (s, 9H, *t*Bu), 7.97 (d, *J* = 8.0 Hz, 0.7H, Ar-H), 8.00 (d, *J* = 8.3 Hz, 0.3H, Ar-H), 8.30 (dd, *J* = 1.4, 7.8 Hz, 0.7H, Ar-H), 8.39 (dd, *J* = 1.4, 7.8 Hz, 0.3H, Ar-H), 8.47–8.50 (m, 1H, Ar-H), 8.53 (s, 0.7H, CH_triazole_), 9.08 (s, 0.3H, CH_triazole_); ^13^C-NMR (125 MHz, (CD_3_)_2_SO) δ 29.1, 29.4 (C(CH_3_)_3_), 59.6, 63.2 (C(CH_3_)_3_), 116.7, 117.2 (Ar-CH), 121.5 (CH_triazole_), 125.1, 125.2 (Ar-CH), 127.6, 128.6 (Ar-C), 129.7, 130.4 (Ar-CH), 132.3 (CH_triazole_), 136.1, 137.1, 141.7, 142.0 (Ar-C), 143.9, 144.2 (C_triazole_), 162.0, 162.3 (C=O); HRMS-ESI [M − H]^−^ Calcd. for C_13_H_13_N_4_O_3_S: 305.0714. Found: 305.0715.

### 3.27. N-t-Butyl-6-N,N-bis([1-{2,3,4,6-tetra-O-acetyl-β-d-glucopyranosyl}-1H-1,2,3-triazol-4-yl]methyl) amino-1,2-benzisothiazole-3-one-1,1-dioxide *(**33**)*

The title compound **33** was prepared from saccharin bis-alkyne **6** (0.108 g, 0.327 mmol) and 2,3,4,6-tetra-*O*-acetyl-β-d-glucopyranosyl azide **d′** [25] (0.244 g, 0.654 mmol) at 45 °C in 2 h according to general procedure 1. Purification of the crude product by flash chromatography (EtOAc/hexane = 1:1 to 7:3) gave the title compound **33** (0.308 g, 87%) as a white solid. m.p. 197–199 °C (EtOAc/hexane); ^1^H-NMR (500 MHz, (CD_3_)_2_SO) δ 1.63 (s, 9H, *t*Bu), 1.73 (s, 6H, OCOCH_3_), 1.95 (s, 6H, OCOCH_3_), 1.99 (s, 6H, OCOCH_3_), 2.02 (s, 6H, OCOCH_3_), 4.07 (dd, *J* = 2.4, 12.5 Hz, 2H, H-6), 4.13 (dd, *J* = 5.5, 12.6 Hz, 2H, H-6), 4.36 (ddd, *J* = 2.5, 5.4 10.2 Hz, 2H, H-5), 4.84 (s, 4H, NCH_2_), 5.15 (dd, *J* = 9.1, 10.1 Hz, 2H, H-4), 5.54 (t, *J* = 9.3 Hz, 2H, H-3), 5.59 (t, *J* = 9.2 Hz, 2H, H-2), 6.34 (d, *J* = 8.8 Hz, 2H, H-1), 7.18 (dd, *J* = 2.4, 9.0 Hz, 1H, Ar-H), 7.44 (d, *J* = 2.4 Hz, 1H, Ar-H), 7.68 (d, *J* = 8.8 Hz, 1H, Ar-H), 8.42 (s, 2H, CH_triazole_); ^13^C-NMR (125 MHz, (CD_3_)_2_SO) δ 19.7, 20.2, 20.4, 20.5 (OCOCH_3_), 27.4 (C(CH_3_)_3_), 45.5 (NCH_2_), 59.6 (C(CH_3_)_3_), 61.7 (C-6), 67.5 (C-4), 70.1 (C-2), 72.0 (C-3), 73.2 (C-5), 83.8 (C-1), 102.5 (Ar-CH), 113.0 (Ar-C), 117.4 (Ar-CH), 122.5 (CH_triazole_), 125.4 (Ar-CH), 139.6 (Ar-C), 143.6 (C_triazole_), 152.8 (Ar-C), 159.9 (C=O), 168.4, 169.3, 169.5, 170.0 (OCOCH_3_); HRMS-ESI [M + Na]^+^ Calcd. for C_45_H_56_N_8_NaO_21_S: 1099.3173. Found: 1099.3144.

### 3.28. N-t-Butyl-6-N,N-bis([1-β-d-glucopyranosyl-1H-1,2,3-triazol-4-yl]methyl)amino-1,2-benzisothiazole- 3-one-1,1-dioxide *(**34**)*

To a solution of **33** (0.110 g, 0.102 mmol) in methanol (9.2 mL) was added HCl (0.8 mL). The reaction was stirred at rt for 90 h and the solvent was removed in vacuo. The residue was purified by RP-18 column chromatography (MeOH/H_2_O = 5:95 to 1:1, product eluting at 1:1) to give the title compound **34** (0.069 g, 92%) as a white solid. m.p. 203–205 °C (MeOH/CH_2_Cl_2_); ^1^H-NMR (500 MHz, (CD_3_)_2_SO) δ 1.65 (s, 9H, *t*Bu), 3.19–3.25 (m, 2H, H-4), 3.35–3.47 (m, 6H, H-3, H-5, H-6), 3.66–3.77 (m, 4H, H-2, H-6), 4.61 (t, *J* = 5.6 Hz, 2H, OH-6), 4.80–4.89 (m, 4H, NCH_2_), 5.15 (d, *J* = 5.5 Hz, 2H, OH-4), 5.27 (d, *J* = 4.9 Hz, 2H, OH-3), 5.36 (d, *J* = 6.0 Hz, 2H, OH-2), 5.53 (d, *J* = 9.2 Hz, 2H, H-1), 7.34 (dd, *J* = 2.4, 9.0 Hz, 1H, Ar-H), 7.56 (d, *J* = 2.3 Hz, 1H, Ar-H), 7.73 (d, *J* = 8.8 Hz, 1H, Ar-H), 8.36 (s, 2H, CH_triazole_); ^13^C-NMR (125 MHz, (CD_3_)_2_SO) δ 27.5 (C(CH_3_)_3_), 45.2 (NCH_2_), 59.6 (C(CH_3_)_3_), 60.7 (C-6), 69.6 (C-4), 72.1 (C-2), 76.9 (C-3), 79.9 (C-5), 87.5 (C-1), 102.2 (Ar-CH), 112.7 (Ar-C), 117.2 (Ar-CH), 122.6 (CH_triazole_), 125.7 (Ar-CH), 139.7 (Ar-C), 142.7 (C_triazole_), 153.0 (Ar-C), 159.9 (C=O); HRMS-ESI [M + Na]^+^ Calcd. for C_29_H_40_N_8_NaO_13_S: 763.2328. Found: 763.2366.

### 3.29. N-t-Butyl-6-(4-benzyl-1H-1,2,3-triazol-1-yl)-1,2-benzisothiazole-3-one-1,1-dioxide *(**35**)*

The title compound **35** was prepared from *N-t*-butyl-6-azido-1,2-benzisothiazole-3-one-1,1-dioxide **3** [10] (0.150 g, 0.535 mmol) and 3-phenyl-1-propyne **i** (0.067 mL, 0.535 mmol) at 45 °C in 2 h according to general procedure 1. Purification of the crude product by flash chromatography (EtOAc/hexane = 1:4 to 1:2) gave the title compound **35** (0.185 g, 87%) as a light brown solid. m.p. 171–173 °C (EtOAc/hexane); ^1^H-NMR (500 MHz, (CD_3_)_2_SO) δ 1.70 (s, 9H, *t*Bu), 4.12 (s, 2H, CH_2_), 7.21–7.26 (m, 1H, Ar-H), 7.31–7.34 (m, 4H, Ar-H), 8.20 (d, *J* = 8.4 Hz, 1H, Ar-H), 8.52 (dd, *J* = 1.9, 8.4 Hz, 1H, Ar-H), 8.78 (d, *J* = 1.9 Hz, 1H, Ar-H), 8.88 (s, 1H, CH_triazole_); ^13^C-NMR (125 MHz, (CD_3_)_2_SO) δ 27.3 (C(CH_3_)_3_), 31.2 (CH_2_), 60.8 (C(CH_3_)_3_), 111.7, (Ar-CH), 121.5 (CH_triazole_), 125.1 (Ar-C), 125.5, 126.4, 126.6, 128.5, 128.6 (Ar-CH), 138.6, 138.7, 141.5 (Ar-C), 148.0 (C_triazole_), 158.7 (C=O); HRMS-ESI [M + Na]^+^ Calcd. for C_20_H_20_N_4_NaO_3_S: 419.1148. Found: 419.1144.

### 3.30. N-t-Butyl-6-[4-(13-hydroxy-2,5,8,11-tetraoxatridec-1-yl)-1H-1,2,3-triazol-1-yl]-1,2-benzisothiazole-3-one-1,1-dioxide *(**36**)*

The title compound **36** was prepared from *N-t*-butyl-6-azido-1,2-benzisothiazole-3-one-1,1-dioxide **3** [10] (0.100 g, 0.357 mmol) and PEG alkyne **j** [31] (0.083 mL, 0.357 mmol) at 45 °C in 2 h according to general procedure 1. Purification of the crude product by flash chromatography (MeOH/CH_2_Cl_2_ = 0:1 to 5:95) gave the title compound **36** (0.135 g, 74%) as a pale yellow oil. ^1^H-NMR (500 MHz, (CD_3_)_2_SO) δ 1.71 (s, 9H, *t*Bu), 3.38–3.41 (m, 2H, CH_2_), 3.44–3.54 (m, 10H, CH_2_), 3.56–3.59 (m, 2H, CH_2_), 3.63–3.66 (m, 2H, CH_2_), 4.55 (t, *J* = 5.4 Hz, 1H, OH), 4.66 (s, 2H, CH_2_), 8.23 (d, *J* = 8.4 Hz, 1H, Ar-H), 8.55 (dd, *J* = 1.9, 8.4 Hz, 1H, Ar-H), 8.82 (d, *J* = 1.9 Hz, 1H, Ar-H), 9.09 (s, 1H, CH_triazole_); ^13^C-NMR (125 MHz, (CD_3_)_2_SO) δ 27.3 (C(CH_3_)_3_), 60.2 (CH_2_), 60.8 (C(CH_3_)_3_), 63.3, 69.2, 69.68, 69.73, 69.8, 72.3 (CH_2_), 111.9, (Ar-CH), 122.9 (CH_triazole_), 125.3 (Ar-C), 125.8, 126.7 (Ar-CH), 138.6, 141.4 (Ar-C), 145.8 (C_triazole_), 158.7 (C=O); HRMS-ESI [M + Na]^+^ Calcd. for C_22_H_32_N_4_NaO_8_S: 535.1833. Found: 535.1845.

### 3.31. N-t-Butyl-6-1H-1,2,3-triazol-1-yl-1,2-benzisothiazole-3-one-1,1-dioxide *(**37**)*

*N*-*t*-Butyl-6-azido-1,2-benzisothiazole-3-one-1,1-dioxide **3 [10]** (0.150 g, 0.535 mmol) and ethynyltrimethylsilane **l** (0.152 mL, 1.07 mmol) were dissolved in *tert*-butyl alcohol/H_2_O (1:1, 8 mL). To the reaction mixture was added a solution of sodium ascorbate (0.042 g, 0.214 mmol) in water (0.25 mL) followed by a solution of CuSO_4_.5H_2_O (0.027 g, 0.107 mmol) in water (0.25 mL). The suspension was stirred vigorously at 45 °C overnight. The solvent was removed in vacuo and the residue was purified by column chromatography on silica gel (EtOAc/hexane = 1:4 to 1:1) to give the title compound **37** (0.073 g, 45%) as a pale yellow solid. m.p. 187–188 °C (EtOAc/hexane); ^1^H-NMR (500 MHz, (CD_3_)_2_SO) δ 1.71 (s, 9H, *t*Bu), 8.09 (d, *J* = 1.2 Hz, 1H, CH_triazole_), 8.24 (d, *J* = 8.4 Hz, 1H, Ar-H), 8.56 (dd, *J* = 1.9, 8.4 Hz, 1H, Ar-H), 8.82 (d, *J* = 1.9 Hz, 1H, Ar-H), 9.11 (d, *J* = 1.3 Hz, 1H, CH_triazole_); ^13^C-NMR (125 MHz, (CD_3_)_2_SO) δ 27.3 (C(CH_3_)_3_), 60.8 (C(CH_3_)_3_), 116.9, (Ar-CH), 124.0 (CH_triazole_), 125.3 (Ar-C), 125.9, 126.7 (Ar-CH), 135.1 (CH_triazole_), 138.6, 141.4 (Ar-C), 158.7 (C=O); HRMS-ESI [M − H]^−^ Calcd. for C_13_H_13_N_4_O_3_S: 305.0714. Found: 305.0712.

### 3.32. 6-(3-t-Butyl-1H-1,2,3-triazol-1-yl)-1,2-benzisothiazole-3-one-1,1-dioxide *(**38**)*

*N*-*t*-Butyl protected **37** (0.080 g, 0.261 mmol) was refluxed in TFA (3 mL) for 18 h. The solvent was removed in vacuo. EtOAc was added to the residue and the solid collected by filtration to give the title compound **38** (0.058 g, 89%) as an off white solid. m.p. 166–168 °C (EtOAc); ^1^H-NMR (500 MHz, (CD_3_)_2_SO) δ 1.71 (s, 9H, *t*Bu), 7.93 (d, *J* = 8.1 Hz, 1H, Ar-H), 8.26 (dd, *J* = 2.0, 8.1 Hz, 1H, Ar-H), 8.45 (d, *J* = 1.9 Hz, 1H, Ar-H), 9.39 (d, *J* = 1.7 Hz, 1H, CH_triazole_), 9.72 (d, *J* = 1.7 Hz, 1H, CH_triazole_); ^13^C-NMR (125 MHz, (CD_3_)_2_SO) δ 28.5 (C(CH_3_)_3_), 66.6 (C(CH_3_)_3_), 113.3, 124.4, 125.4 (Ar-CH), 129.2, 129.8 (CH_triazole_), 136.7, 136.8, 147.0 (Ar-C), 165.9 (C=O); HRMS-ESI [M + H]^+^ Calcd. for C_13_H_13_N_4_O_3_S: 305.0714. Found: 305.0702.

### 3.33. N-t-Butyl-6-(4-{2,3,4,6-tetra-O-acetyl-[β-d-glucopyranosyl]thiomethyl}-1H-1,2,3-triazol-1-yl)-1,2- benzisothiazole-3-one-1,1-dioxide *(**39**)*

The title compound **39** was prepared from N-*t*-butyl-6-amino-1,2-benzisothiazole-3-one-1,1-dioxide **3** [10] (0.300 g, 1.07 mmol) and propargyl 2,3,4,6-tetra-*O*-acetyl-thio-β-d-glucopyranoside **k′ [14]** (0.431 g, 1.07 mmol) at 40 °C in 2 h according to general procedure 1. Purification of the crude product by flash chromatography (EtOAc/hexane = 2:3 to 1:1) gave the title compound **39** (0.656 g, 90%) as a white solid. m.p. 96–98 °C (EtOAc/hexane); ^1^H-NMR (500 MHz, (CD_3_)_2_SO) δ 1.71 (s, 9H, *t*Bu), 1.94 (s, 3H, OCOCH_3_), 1.98 (s, 6H, 2 × OCOCH_3_), 1.99 (s, 3H, OCOCH_3_), 3.99–4.16 (m, 5H, H-5, SCH_2_, CH_2_-6), 4.91–5.00 (m, 3H, H-1, H-2, H-4), 5.31 (t, *J* = 9.0 Hz, 1H, H-3), 8.24 (d, *J* = 8.4 Hz, 1H, Ar-H), 8.52 (dd, *J* = 1.9, 8.4 Hz, 1H, Ar-H), 8.78 (d, *J* = 1.9 Hz, 1H, Ar-H), 8.95 (s, 1H, CH_triazole_); ^13^C-NMR (125 MHz, (CD_3_)_2_SO) δ 20.3, 20.4, 20.4, 20.5 (OCOCH_3_), 23.1 (SCH_2_), 27.3 (C(CH_3_)_3_), 60.8 (C(CH_3_)_3_), 61.8 (C-6), 68.1 (C-4), 69.6 (C-2), 72.9 (C-3), 74.4 (C-5), 80.9 (C-1), 111.9, (Ar-CH), 122.4 (CH_triazole_), 125.3 (Ar-C), 125.7, 126.7 (Ar-CH), 138.6, 141.4 (Ar-C), 145.3 (C_triazole_), 158.7 (C=O), 169.1, 169.2, 169.5, 170.0 (OCOCH_3_); HRMS-ESI [M + Na]^+^ Calcd. for C_28_H_34_N_4_NaO_12_S_2_: 705.1507. Found: 705.1551.

### 3.34. N-t-Butyl-6-(4-{[β-d-glucopyranosyl]thiomethyl}-1H-1,2,3-triazol-1-yl)-1,2-benzisothiazole-3-one-1,1-dioxide *(**40**)*

The title compound **40** was prepared using two different synthetic routes, A and B.

- The title compound **40** was prepared from *N*-*t*-butyl-6-amino-1,2-benzisothiazole-3-one-1,1-dioxide **3** [10] (0.200 g, 0.714 mmol) and propargyl thio-β-d-glucopyranoside (**k**) [32] (0.167 g, 0.714 mmol) at 40 °C in 2 h according to general procedure 1. Purification of the crude product by flash chromatography (MeOH/CH_2_Cl_2_ = 0:1 to 3:17) gave the title compound **40** (0.324 g, 88%) as a white solid.
- To a solution of **39** (0.385 g, 0.564 mmol) in methanol (9.2 mL) was added HCl (0.8 mL). The reaction was stirred at r.t. for 90 h and the solvent was removed in vacuo. The residue was purified by column chromatography on silica gel (MeOH/CH_2_Cl_2_ = 0:1 to 1:9) to give the title compound **40** (0.255 g, 88%) as a white solid. m.p. 155–157 °C (MeOH/CH_2_Cl_2_); ^1^H-NMR (500 MHz, (CD_3_)_2_SO) δ 1.71 (s, 9H, *t*Bu), 3.03–3.11 (m, 2H, H-2, H-4), 3.15 (dd, *J* = 4.8, 8.6 Hz, 1H, H-3), 3.20 (ddd, *J* = 2.0, 6.5, 8.6 Hz, 1H, H-5), 3.42–3.48 (m, 1H, H-6), 3.73 (ddd, *J* = 2.0, 5.9, 11.9 Hz, 1H, H-6), 3.97 (d, *J* = 14.4 Hz, 1H, SCH_2_), 4.10 (d, *J* = 14.4 Hz, 1H, SCH_2_), 4.36 (d, *J* = 9.6 Hz, 1H, H-1), 4.70 (t, *J* = 5.8 Hz, 1H, OH-6), 4.97 (d, *J* = 5.3 Hz, 1H, OH-4), 5.05 (d, *J* = 4.8 Hz, 1H, OH-3), 5.18 (d, *J* = 5.9 Hz, 1H, OH-2), 8.22 (d, *J* = 8.4 Hz, 1H, Ar-H), 8.50 (dd, *J* = 1.9, 8.4 Hz, 1H, Ar-H), 8.76 (d, *J* = 1.9 Hz, 1H, Ar-H), 8.97 (s, 1H, CH_triazole_); ^13^C-NMR (125 MHz, (CD_3_)_2_SO) δ 22.9 (SCH_2_), 27.3 (C(CH_3_)_3_), 60.8 (C(CH_3_)_3_), 61.3 (C-6), 70.1 (C-4), 73.1 (C-2), 78.1 (C-3), 81.0 (C-5), 84.1 (C-1), 111.8, (Ar-CH), 122.2 (CH_triazole_), 125.2 (Ar-C), 125.7, 126.7 (Ar-CH), 138.6, 141.4 (Ar-C), 146.6 (C_triazole_), 158.7 (C=O); HRMS-ESI [M + H]^+^ Calcd. for C_20_H_27_N_4_O_8_S_2_: 515.1265. Found: 515.1269.

### 3.35. N-t-Butyl-6-(4-{2,3,4,6-tetra-O-acetyl-[β-d-glucopyranosyl]sulfonylmethyl}-1H-1,2,3-triazol-1-yl)-1,2-benzisothiazole-3-one-1,1-dioxide *(**41**)*

To a stirred solution of **39** (0.250 g, 0.366 mmol) in anhydrous CH_2_Cl_2_ (5 mL) at 0 °C was added *m*CPBA (0.737 g, 2.56 mmol) in anhydrous CH_2_Cl_2_ (2 mL) dropwise. The solution was allowed to warm to r.t. over 2 h. The reaction mixture was diluted with CH_2_Cl_2_ (50 mL), washed with H_2_O (30 mL), brine (30 mL), dried (MgSO_4_), and the solvent was removed in vacuo. The residue was purified by column chromatography on silica gel (EtOAc/hexane = 1:1 to 3:2) to give the title compound **41** (0.260 g, 99%) as a white solid. m.p. 172–174 °C (EtOAc/hexane); ^1^H-NMR (500 MHz, (CD_3_)_2_SO) δ 1.72 (s, 9H, *t*Bu), 1.94 (s, 3H, OCOCH_3_), 1.95 (s, 3H, OCOCH_3_), 2.00 (s, 3H, OCOCH_3_), 2.03 (s, 3H, OCOCH_3_), 4.17–4.28 (m, 3H, H-5, CH_2_-6), 4.78–4.87 (m, 2H, SCH_2_), 5.01–5.06 (m, 1H, H-4), 5.14–5.20 (m, 1H, H-1), 5.37–5.45 (m, 2H, H-2, H-3), 8.26 (d, *J* = 8.4 Hz, 1H, Ar-H), 8.59 (dd, *J* = 2.0, 8.5 Hz, 1H, Ar-H), 8.89 (d, *J* = 2.0 Hz, 1H, Ar-H), 9.14 (s, 1H, CH_triazole_); ^13^C-NMR (125 MHz, (CD_3_)_2_SO) δ 20.2, 20.29, 20.33, 20.5 (OCOCH_3_), 27.3 (C(CH_3_)_3_), 46.9 (SCH_2_), 60.9 (C(CH_3_)_3_), 61.4 (C-6), 65.7 (C-2), 67.2 (C-4), 72.5 (C-3), 74.9 (C-5), 84.9 (C-1), 112.3, (Ar-CH), 124.8 (CH_triazole_), 125.6 (Ar-C), 126.1, 126.7 (Ar-CH), 135.8, 138.6 (Ar-C), 141.2 (C_triazole_), 158.7 (C=O), 168.6, 169.2, 169.5, 170.1 (OCOCH_3_); HRMS-ESI [M + Na]^+^ Calcd. for C_28_H_34_N_4_NaO_14_S_2_: 737.1405. Found: 737.1486.

### 3.36. N-t-Butyl-6-(4-{[β-d-glucopyranosyl]sulfonylmethyl}-1H-1,2,3-triazol-1-yl)-1,2-benzisothiazole-3-one-1,1-dioxide *(**42**)*

The title compound **42** was prepared using two different synthetic routes, A and B.

- To a stirred solution of **40** (0.150 g, 0.292 mmol) in anhydrous CH_2_Cl_2_ (5 mL) at 0 °C was added *m*CPBA (0.587 g, 2.04 mmol) in anhydrous CH_2_Cl_2_ (2 mL) dropwise. The solution was allowed to warm to rt over 2 h and the solvent was removed in vacuo. The residue was purified by column chromatography on silica gel (MeOH/CH_2_Cl_2_ = 1:9 to 3:17) to give the title compound **42** (0.126 g, 79%) as a white solid.
- To a solution of **41** (0.220 g, 0.308 mmol) in methanol (9.2 mL) was added HCl (0.8 mL). The reaction was stirred at rt for 90 h and the solvent was removed in vacuo. The residue was purified by column chromatography on silica gel (MeOH/CH_2_Cl_2_ = 0:1 to 1:9) to give the title compound **42** (0.117 g, 69%) as a white solid. m.p. 145–146 °C (MeOH/CH_2_Cl_2_); ^1^H-NMR (500 MHz, (CD_3_)_2_SO) δ 1.72 (s, 9H, *t*Bu), 3.08 (ddd, *J* = 5.4, 8.8, 9.8 Hz, 1H, H-4), 3.28 (td, *J* = 5.5, 8.8 Hz, 1H, H-3), 3.42 (ddd, *J* = 1.9, 6.8, 9.8 Hz, 1H, H-5), 3.51 (ddd, *J* = 5.5, 6.9, 12.1 Hz, 1H, H-6), 3.60 (td, *J* = 6.1, 9.1 Hz, 1H, H-2), 3.80 (ddd, *J* = 1.9, 6.2, 12.2 Hz, 1H, H-6), 4.51 (d, *J* = 9.5 Hz, 1H, H-1), 4.73 (d, *J* = 14.7 Hz, 1H, SCH_2_), 4.85 (d, *J* = 14.7 Hz, 1H, SCH_2_), 4.99 (t, *J* = 5.8 Hz, 1H, OH-6), 5.17 (d, *J* = 5.5 Hz, 1H, OH-4), 5.24 (d, *J* = 5.5 Hz, 1H, OH-3), 5.55 (d, *J* = 6.1 Hz, 1H, OH-2), 8.25 (d, *J* = 8.4 Hz, 1H, Ar-H), 8.51 (dd, *J* = 2.0, 8.4 Hz, 1H, Ar-H), 8.81 (d, *J* = 1.9 Hz, 1H, Ar-H), 9.18 (s, 1H, CH_triazole_); ^13^C-NMR (125 MHz, (CD_3_)_2_SO) δ 27.3 (C(CH_3_)_3_), 48.2 (SCH_2_), 60.9 (C(CH_3_)_3_), 61.0 (C-6), 69.2 (C-2), 69.5 (C-4), 77.5 (C-3), 81.6 (C-5), 88.7 (C-1), 112.2, (Ar-CH), 124.8 (CH_triazole_), 125.5 (Ar-C), 126.0, 126.8 (Ar-CH), 137.0, 138.6 (Ar-C), 141.3 (C_triazole_), 158.7 (C=O); HRMS-ESI [M + Na]^+^ Calcd. for C_20_H_26_N_4_NaO_10_S_2_: 569.0983. Found: 569.0994.

## 4. Conclusions

In summary, we have demonstrated that specifically functionalized derivatives of **1** may be prepared from either saccharin azide or saccharin alkyne building blocks in high yield using CuAAC. The application of novel alkyne building blocks **5** and **6** and azide building block **3** proved remarkably straightforward to handle. Moreover, the novel target compounds all retain the capability for interactions with the metal centres of metalloenzymes via the sulfonamide anion. The compounds also present two orientations of the 1,2,3-triazole, thus further adding diversity to the potential hydrogen bonding interactions of these compounds with biomolecules of therapeutic interest. Collectively the novel compounds described here represent desirable attributes not found in the more usual N-alkylated saccharin derivatives.

## Supplementary Materials

Supplementary materials are available online.
