# Supplementary material for: Synthesis of Novel Saccharin Derivatives
Source: Molecules. 2017 Mar 23;22(4):516. doi: 10.3390/molecules22040516 (PMC6153755; doi:10.3390/molecules22040516)

`

SUPPLEMENTARY MATERIALS

Synthesis of novel saccharin derivatives

Gregory M. Rankin and Sally-Ann Poulsen*

Griffith Institute for Drug Discovery, Griffith University, Don Young Road, Nathan, Queensland 4111, Australia

E-mail: s.poulsen@griffith.edu.au

^{1}H, ^{13}C and ^{19}F NMR spectra

Compound **5** 500 MHz ^{1}H NMR $CDCl_3$ S4
Compound **5** 125 MHz ^{13}C NMR $CDCl_3$ S5
Compound **6** 500 MHz ^{1}H NMR $(CD_3)_2SO$ S6
Compound **6** 125 MHz ^{13}C NMR $(CD_3)_2SO$ S7
Compound **8** 500 MHz ^{1}H NMR $CDCl_3$ S8
Compound **8** 125 MHz ^{13}C NMR $CDCl_3$ S9
Compound **9** 500 MHz ^{1}H NMR $CDCl_3$ S10
Compound **9** 125 MHz ^{13}C NMR $CDCl_3$ S11
Compound **10** 500 MHz ^{1}H NMR $(CD_3)_2SO$ S12
Compound **10** 125 MHz ^{13}C NMR $(CD_3)_2SO$ S13
Compound **11** 500 MHz ^{1}H NMR $(CD_3)_2SO$ S14
Compound **11** 125 MHz ^{13}C NMR $(CD_3)_2SO$ S15
Compound **12** 500 MHz ^{1}H NMR $(CD_3)_2SO$ S16
Compound **12** 125 MHz ^{13}C NMR $(CD_3)_2SO$ S17
Compound **13** 500 MHz ^{1}H NMR $(CD_3)_2SO$ S18
Compound **13** 125 MHz ^{13}C NMR $(CD_3)_2SO$ S19
Compound **14** 376 MHz ^{19}F NMR $(CD_3)_2SO$ S20
Compound **14** 500 MHz ^{1}H NMR $(CD_3)_2SO$ S21

Compound **14** 125 MHz ^{13}C NMR $(CD_3)_2SO$ S22
Compound **15** 500 MHz ^1H NMR $(CD_3)_2SO$ S23
Compound **15** 125 MHz ^{13}C NMR $(CD_3)_2SO$ S24
Compound **17** 500 MHz ^1H NMR $(CD_3)_2SO$ S25
Compound **17** 125 MHz ^{13}C NMR $(CD_3)_2SO$ S26
Compound **19** 500 MHz ^1H NMR $(CD_3)_2SO$ S27
Compound **19** 125 MHz ^{13}C NMR $(CD_3)_2SO$ S28
Compound **20** 500 MHz ^1H NMR $(CD_3)_2SO$ S29
Compound **20** 125 MHz ^{13}C NMR $(CD_3)_2SO$ S30
Compound **21** 500 MHz ^1H NMR $(CD_3)_2SO$ S31
Compound **21** 125 MHz ^{13}C NMR $(CD_3)_2SO$ S32
Compound **22** 500 MHz ^1H NMR $(CD_3)_2SO$ S33
Compound **22** 125 MHz ^{13}C NMR $(CD_3)_2SO$ S34
Compound **24** 500 MHz ^1H NMR $(CD_3)_2SO$ S35
Compound **24** 125 MHz ^{13}C NMR $(CD_3)_2SO$ S36
Compound **25** 500 MHz ^1H NMR $(CD_3)_2SO$ S37
Compound **25** 125 MHz ^{13}C NMR $(CD_3)_2SO$ S38
Compound **26** 500 MHz ^1H NMR $(CD_3)_2SO$ S39
Compound **26** 125 MHz ^{13}C NMR $(CD_3)_2SO$ S40
Compound **27** 500 MHz ^1H NMR $(CD_3)_2SO$ S41
Compound **27** 125 MHz ^{13}C NMR $(CD_3)_2SO$ S42
Compound **28** 500 MHz ^1H NMR $(CD_3)_2SO$ S43
Compound **28** 125 MHz ^{13}C NMR $(CD_3)_2SO$ S44
Compound **29** 500 MHz ^1H NMR $(CD_3)_2SO$ S45
Compound **29** 125 MHz ^{13}C NMR $(CD_3)_2SO$ S46
Compound **29** 376 MHz ^{19}F NMR $(CD_3)_2SO$ S47
Compound **30** 500 MHz ^1H NMR $(CD_3)_2SO$ S48
Compound **30** 125 MHz ^{13}C NMR $(CD_3)_2SO$ S49
Compound **31** 500 MHz ^1H NMR $(CD_3)_2SO$ S50
Compound **31** 125 MHz ^{13}C NMR $(CD_3)_2SO$ S51
Compound **31** HSQC NMR $(CD_3)_2SO$ S52
Compound **32a + 32b** 500 MHz ^1H NMR $(CD_3)_2SO$ S53
Compound **32a + 32b** 125 MHz ^{13}C NMR $(CD_3)_2SO$ S54
Compound **33** 500 MHz ^1H NMR $(CD_3)_2SO$ S55
Compound **33** 125 MHz ^{13}C NMR $(CD_3)_2SO$ S56
Compound **34** 500 MHz ^1H NMR $(CD_3)_2SO$ S57

`

Compound **34** 125 MHz ^{13}C NMR $(CD_3)_2SO$ S58
Compound **35** 500 MHz ^{1}H NMR $(CD_3)_2SO$ S59
Compound **35** 125 MHz ^{13}C NMR $(CD_3)_2SO$ S60
Compound **36** 500 MHz ^{1}H NMR $(CD_3)_2SO$ S61
Compound **36** 125 MHz ^{13}C NMR $(CD_3)_2SO$ S62
Compound **37** 500 MHz ^{1}H NMR $(CD_3)_2SO$ S63
Compound **37** 125 MHz ^{13}C NMR $(CD_3)_2SO$ S64
Compound **38** 500 MHz ^{1}H NMR $(CD_3)_2SO$ S65
Compound **38** 125 MHz ^{13}C NMR $(CD_3)_2SO$ S66
Compound **39** 500 MHz ^{1}H NMR $(CD_3)_2SO$ S67
Compound **39** 125 MHz ^{13}C NMR $(CD_3)_2SO$ S68
Compound **40** 500 MHz ^{1}H NMR $(CD_3)_2SO$ S69
Compound **40** 125 MHz ^{13}C NMR $(CD_3)_2SO$ S70
Compound **41** 500 MHz ^{1}H NMR $(CD_3)_2SO$ S71
Compound **41** 125 MHz ^{13}C NMR $(CD_3)_2SO$ S72
Compound **42** 500 MHz ^{1}H NMR $(CD_3)_2SO$ S73
Compound **42** 125 MHz ^{13}C NMR $(CD_3)_2SO$ S74

Compound **5** 500 MHz ^{1}H NMR $CDCl_3$

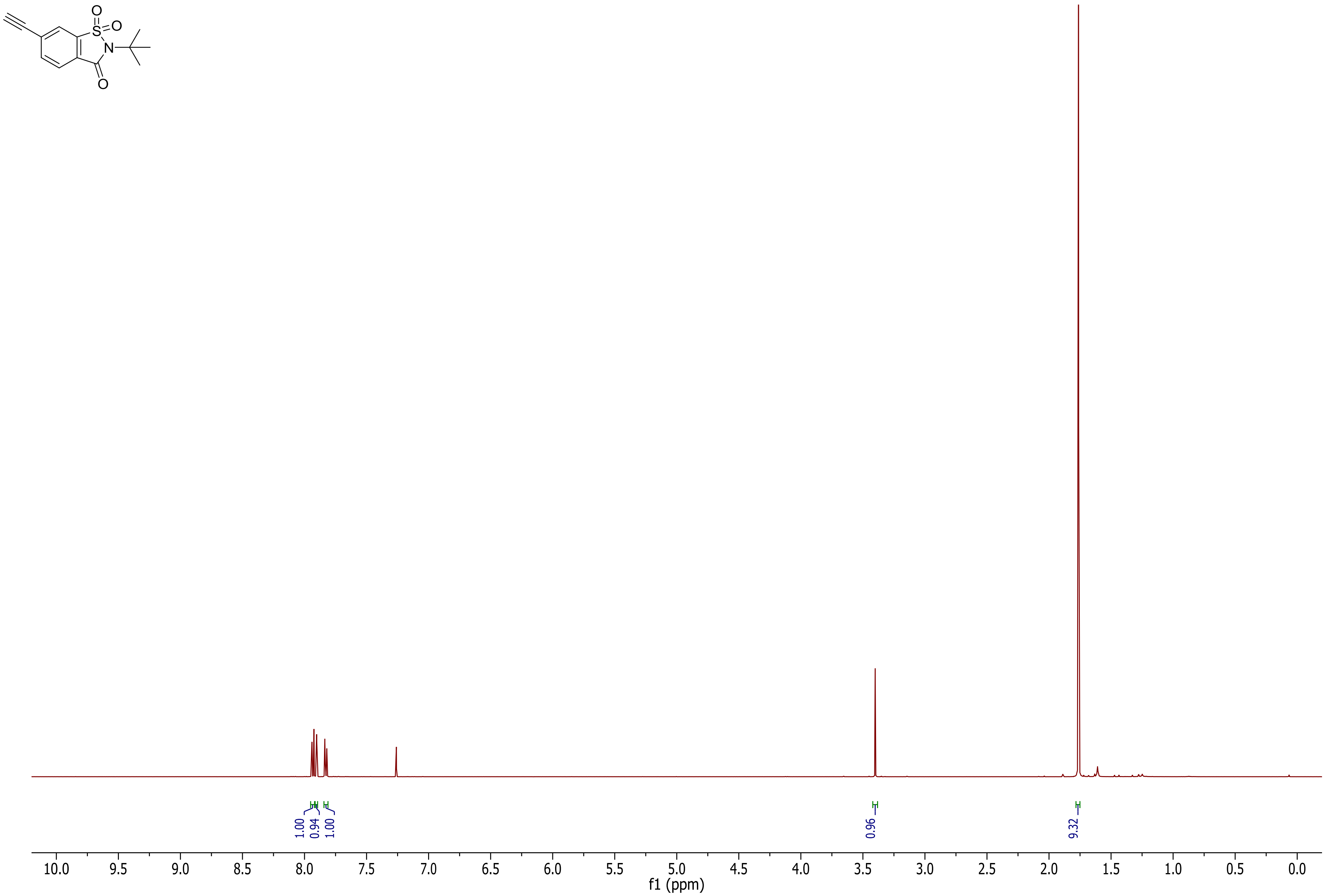

Compound **5** 125 MHz ^{13}C NMR $CDCl_3$

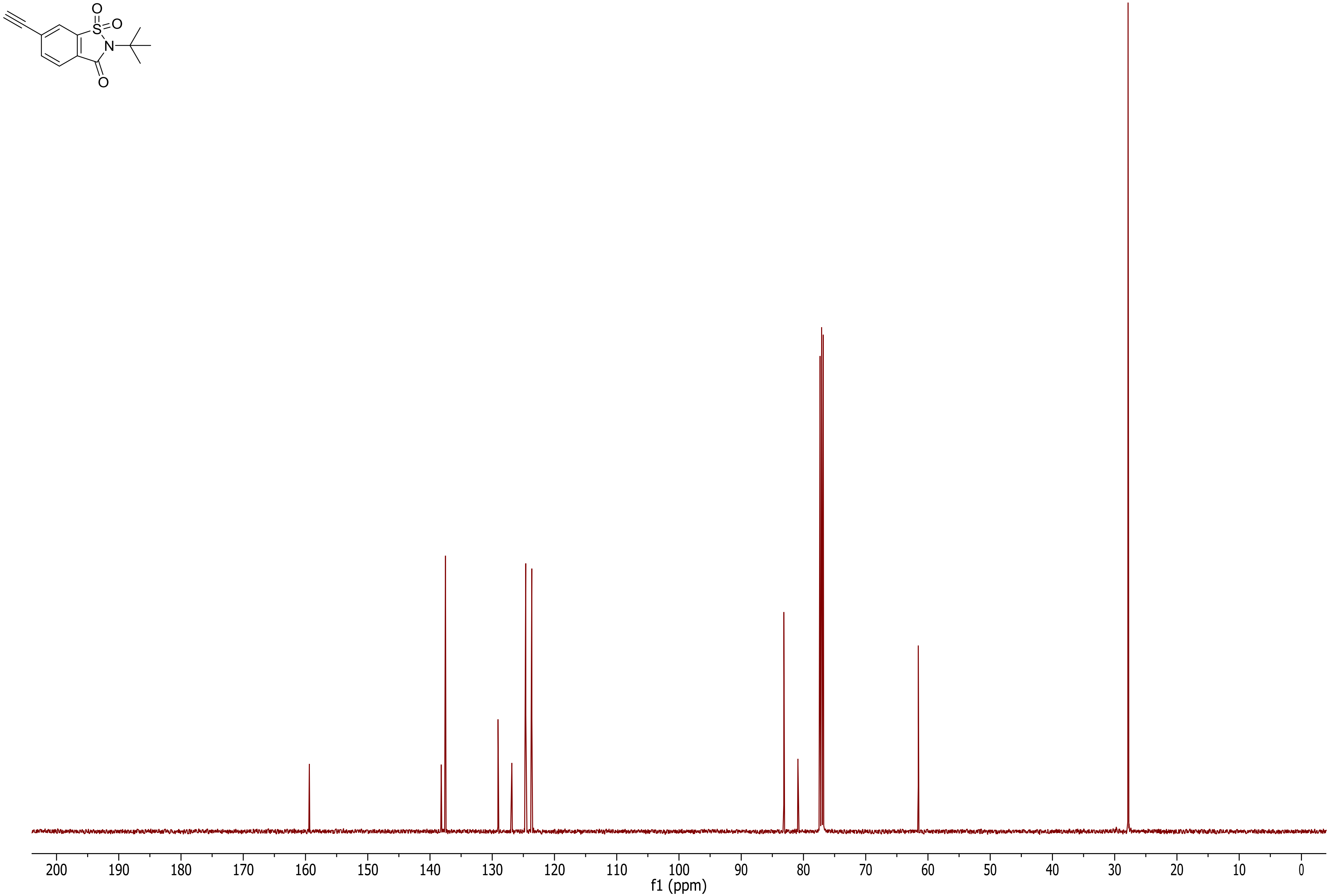

Compound **6** 500 MHz ^1H NMR $(CD_3)_2SO$

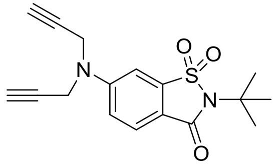

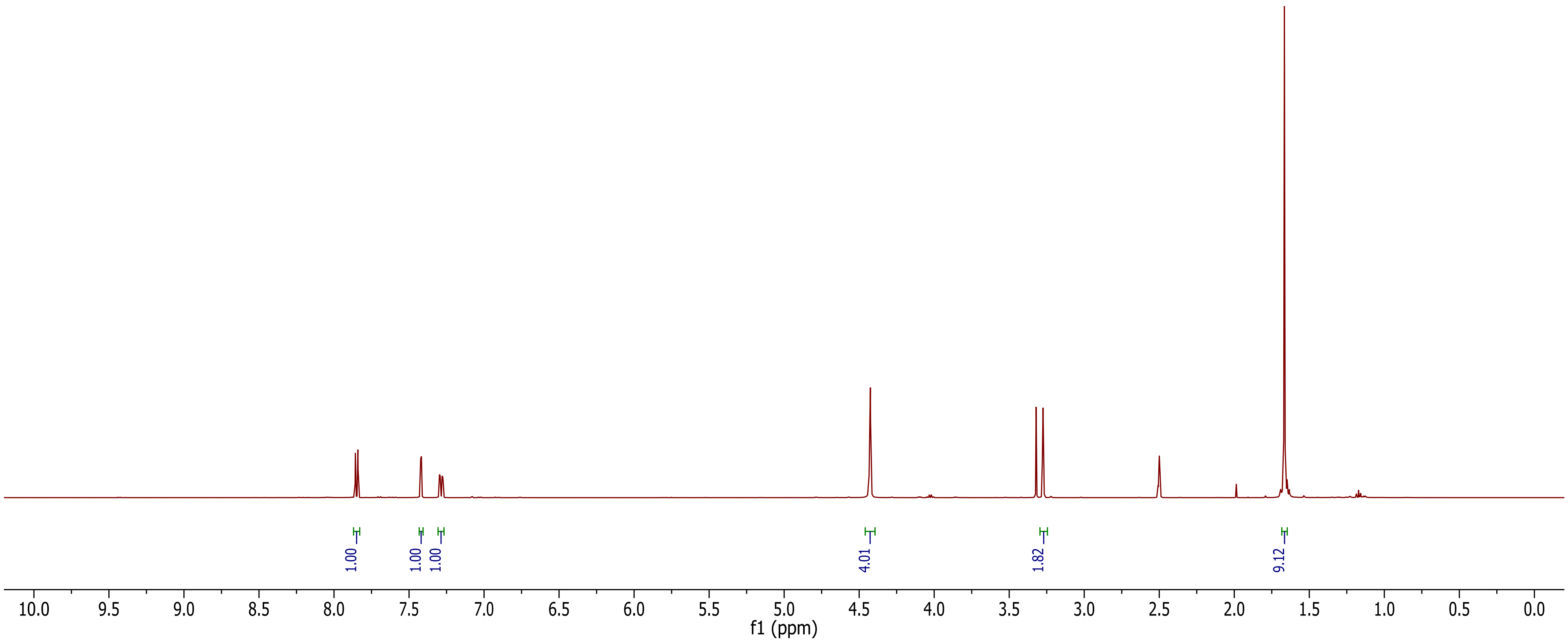

Compound **6** 125 MHz ^{13}C NMR $(CD_3)_2SO$

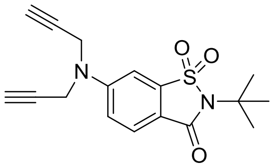

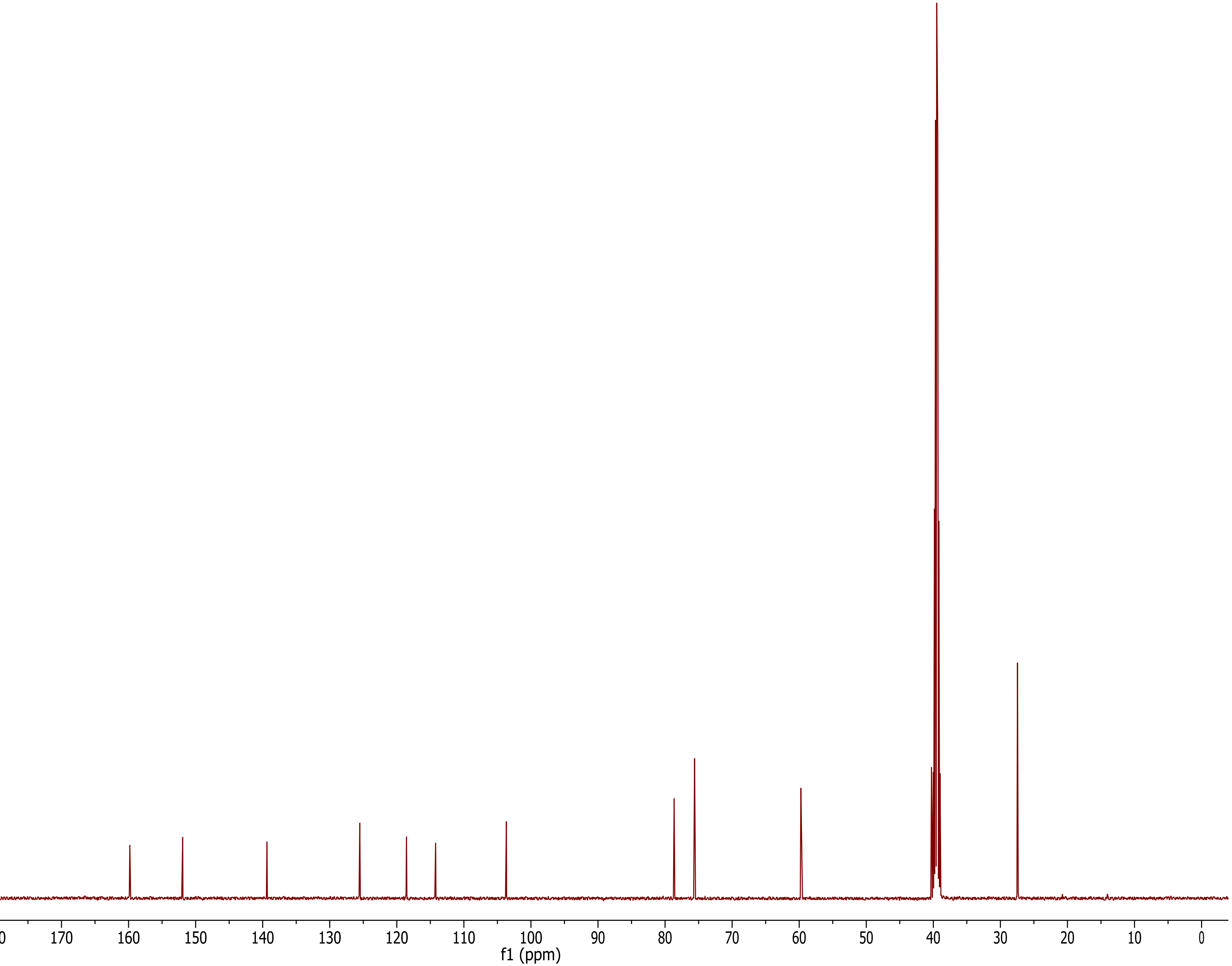

Compound **8** 500 MHz ^{1}H NMR $CDCl_3$

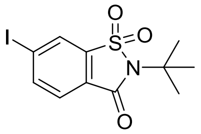

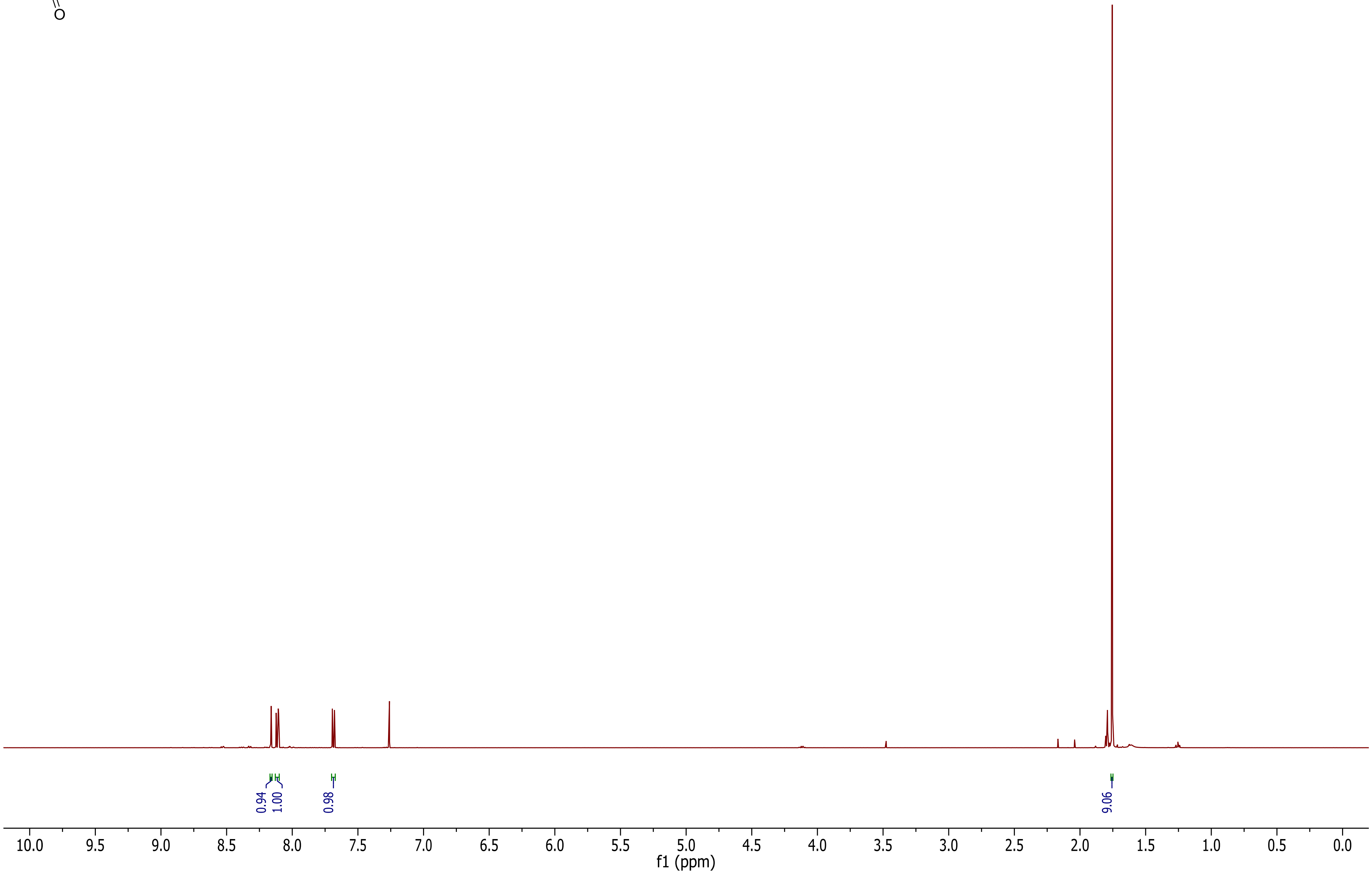

Compound **8** 125 MHz ^{13}C NMR $CDCl_3$

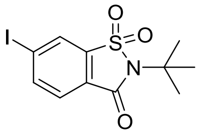

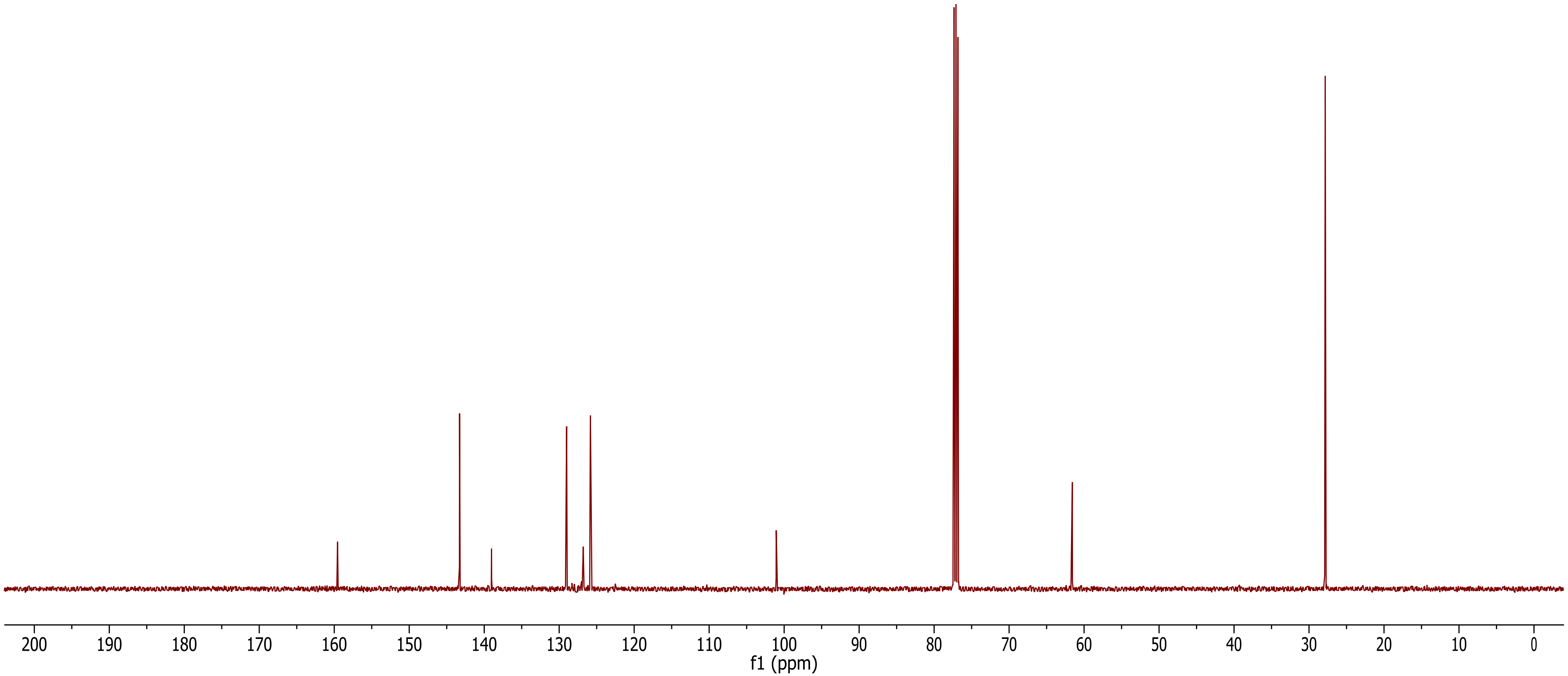

Compound **9** 500 MHz ^{1}H NMR $CDCl_3$

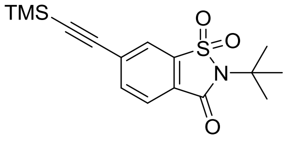

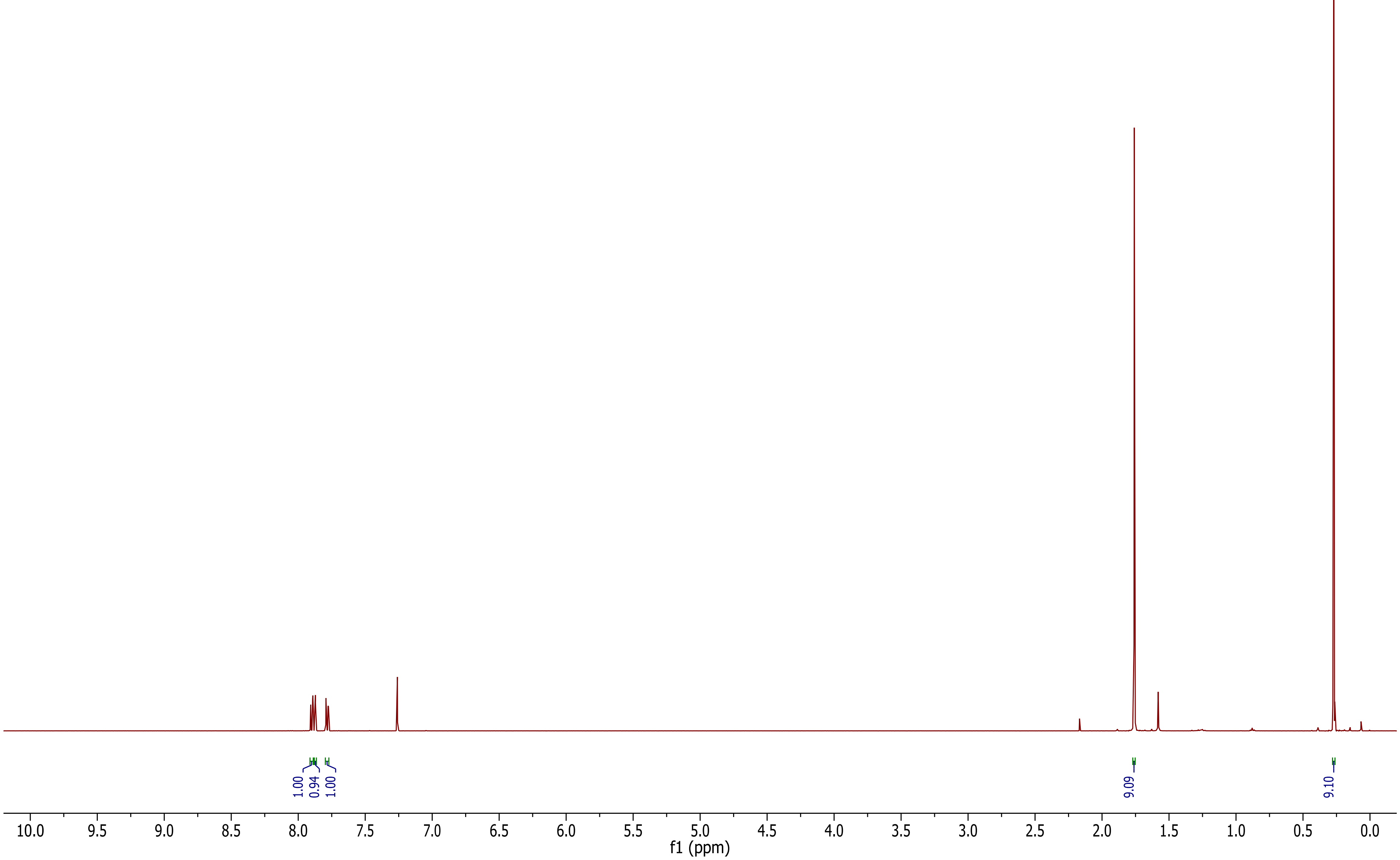

Compound **9** 125 MHz ^{13}C NMR $CDCl_3$

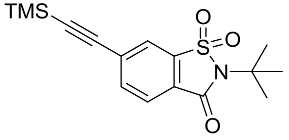

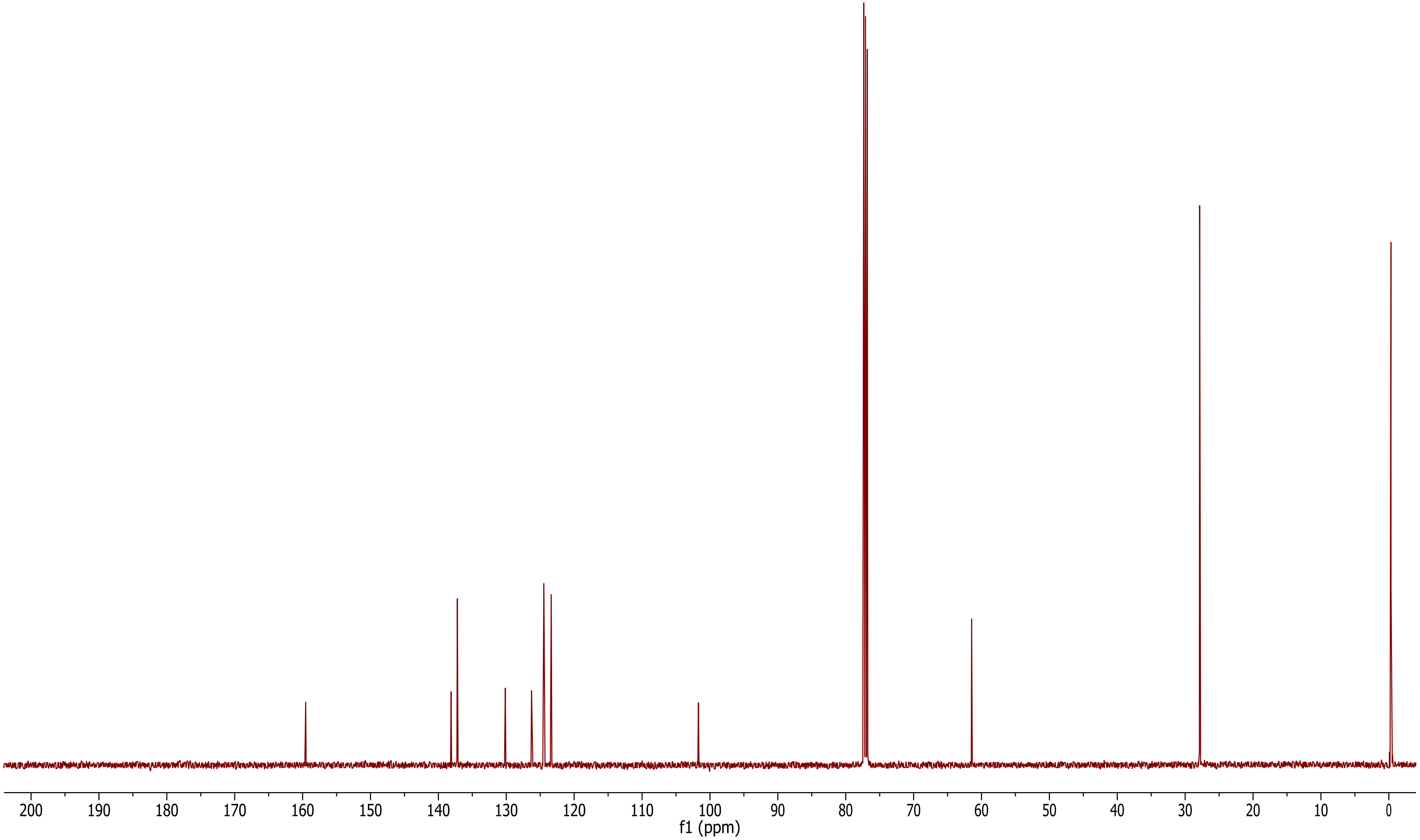

Compound **10** 500 MHz ^{1}H NMR $(CD_3)_2SO$

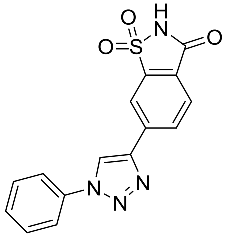

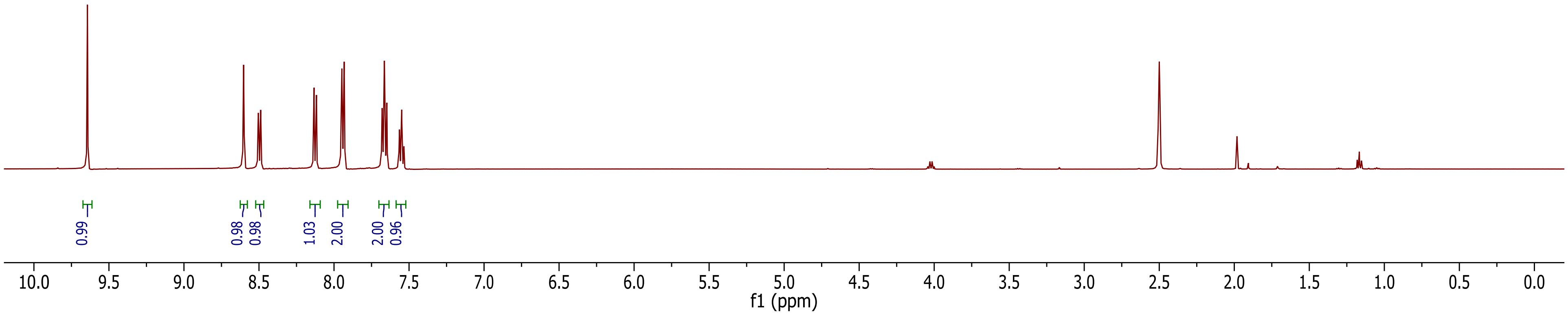

Compound **10** 125 MHz ^{13}C NMR $(CD_3)_2SO$

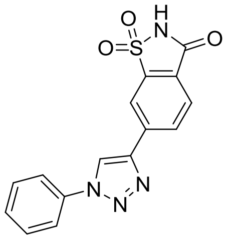

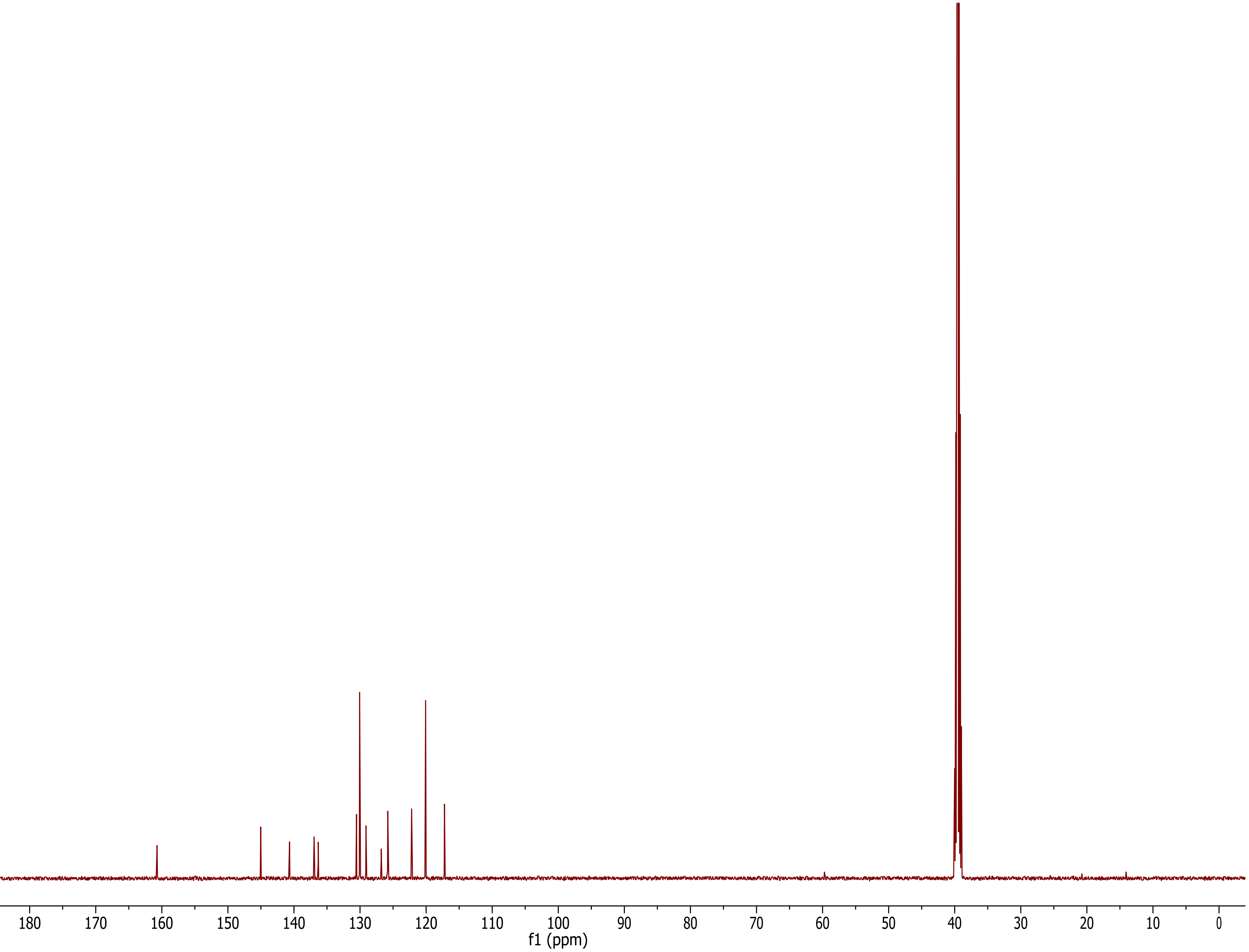

Compound **11** 500 MHz ^{1}H NMR $(CD_3)_2SO$

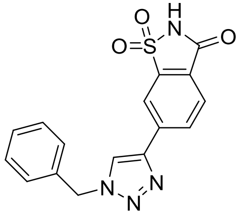

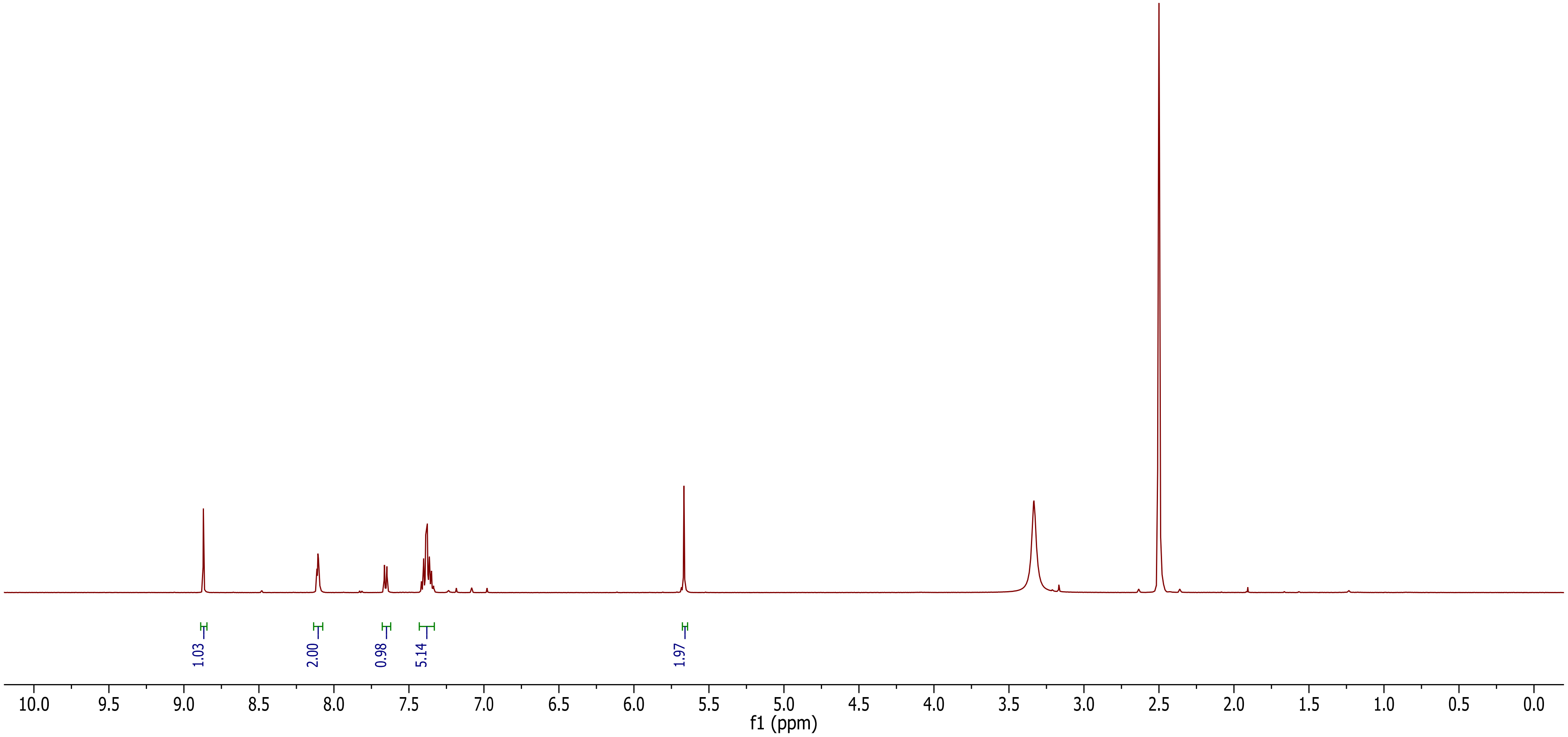

Compound **11** 125 MHz ^{13}C NMR $(CD_3)_2SO$

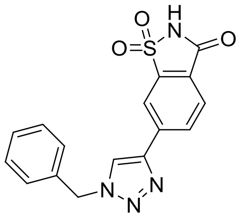

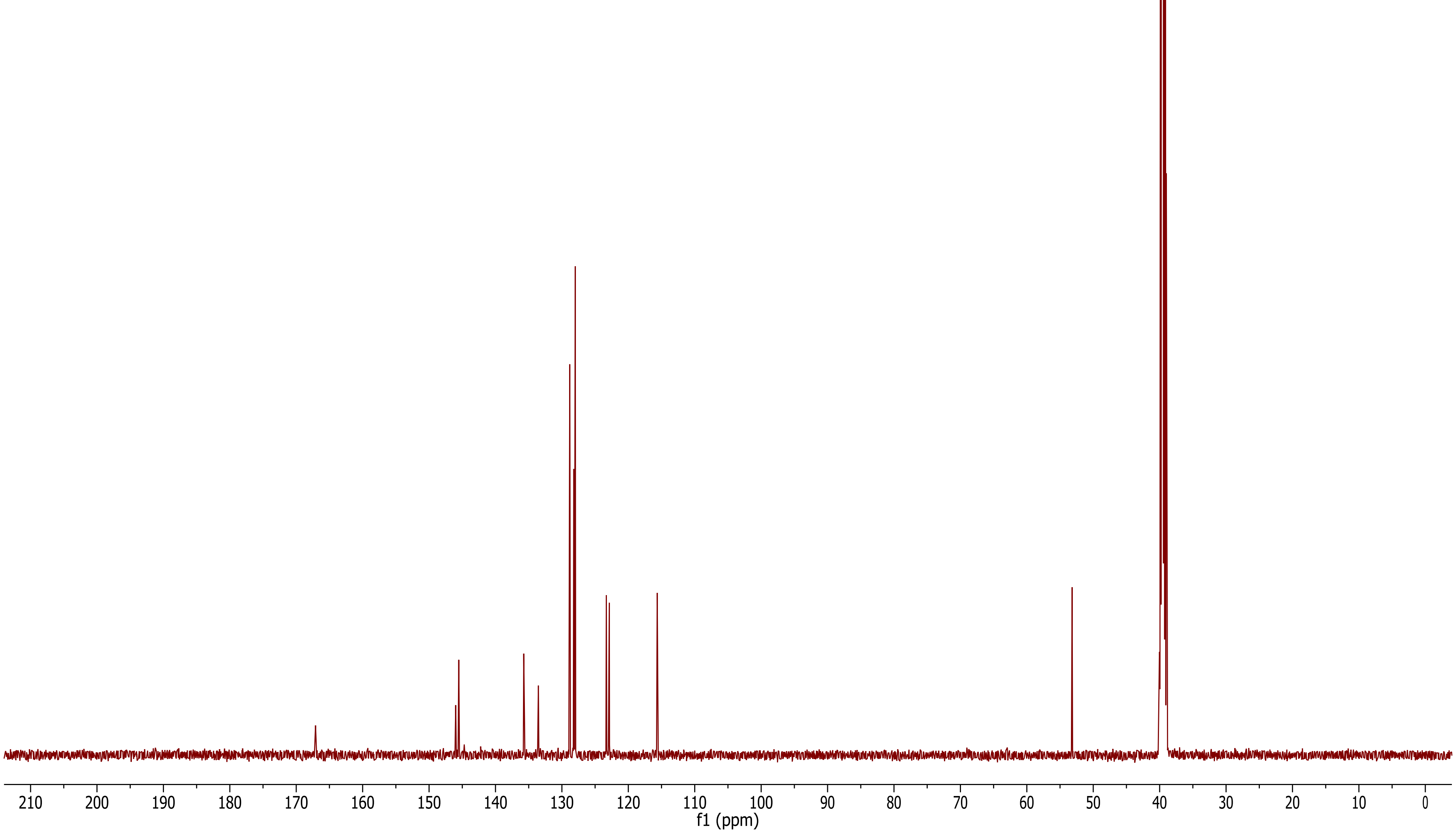

Compound **12** 500 MHz ^{1}H NMR $(CD_3)_2SO$

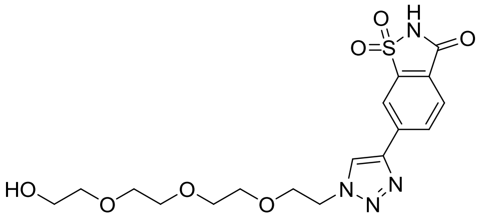

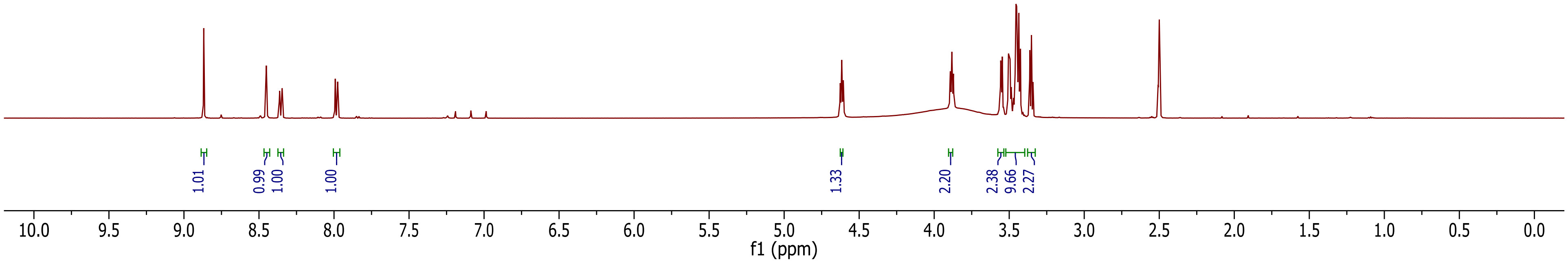

Compound **12** 125 MHz ^{13}C NMR $(CD_3)_2SO$

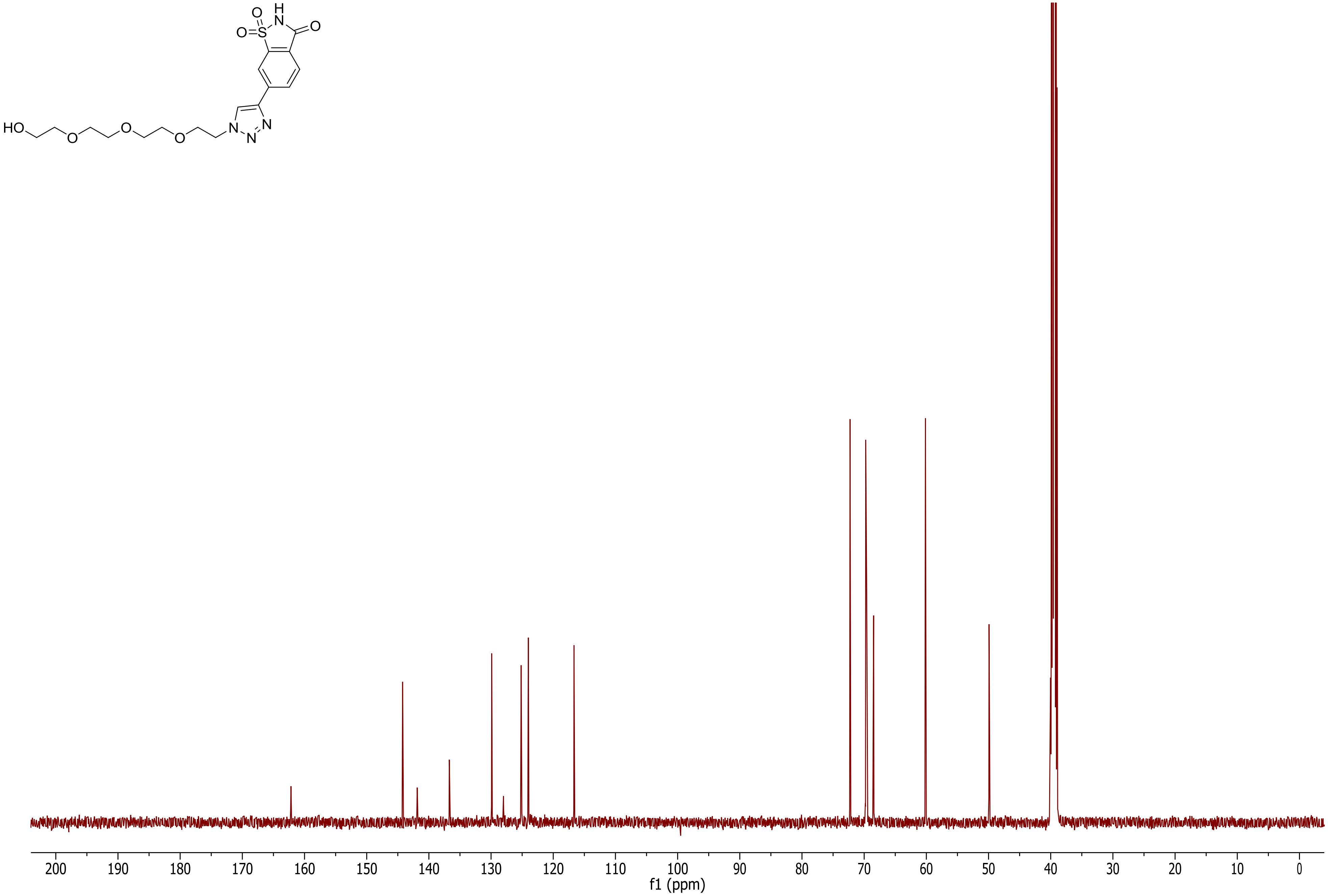

Compound **13** 500 MHz ^{1}H NMR $(CD_3)_2SO$

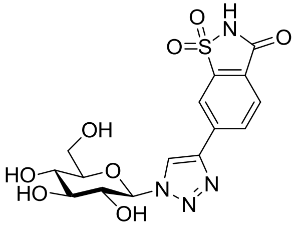

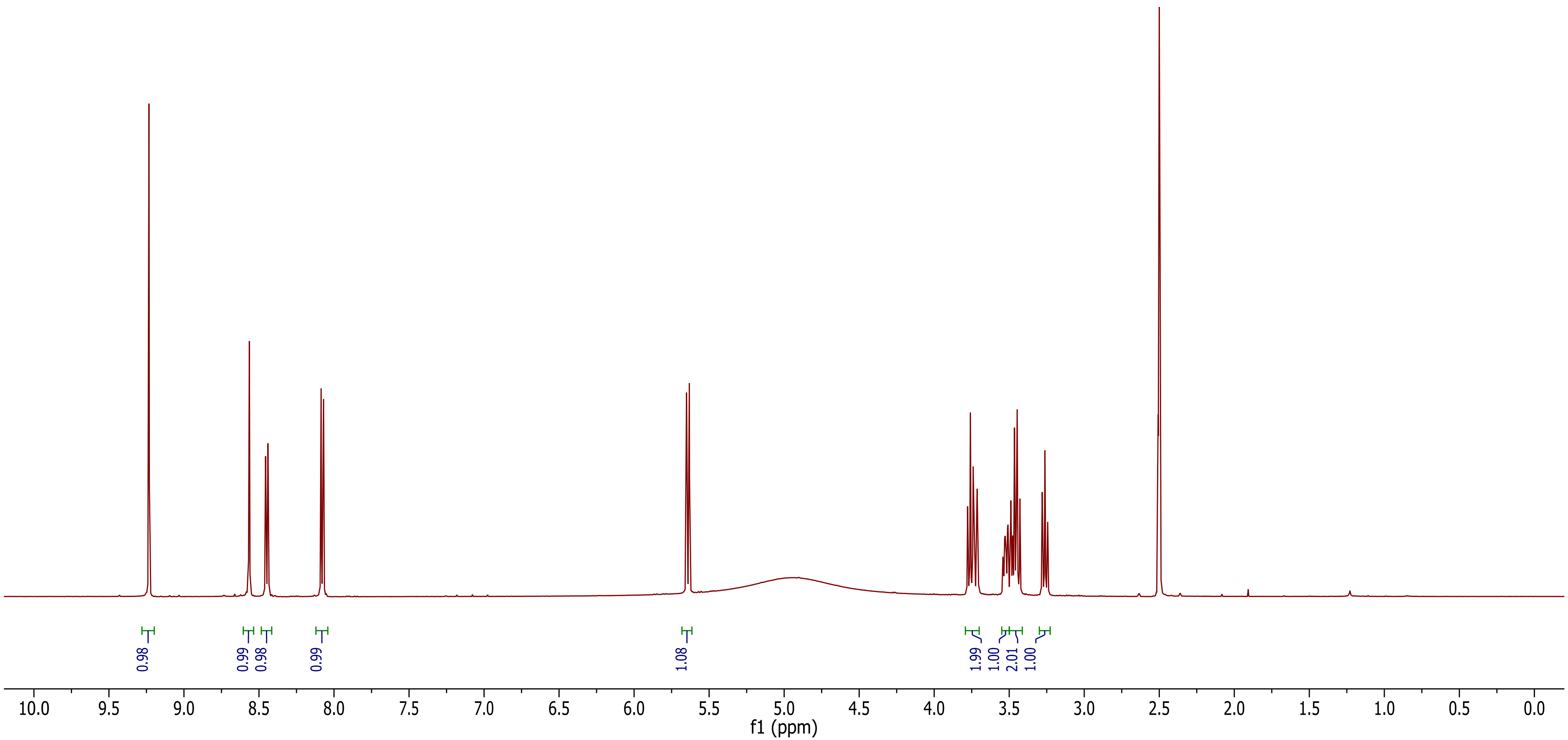

Compound **13** 125 MHz ^{13}C NMR $(CD_3)_2SO$

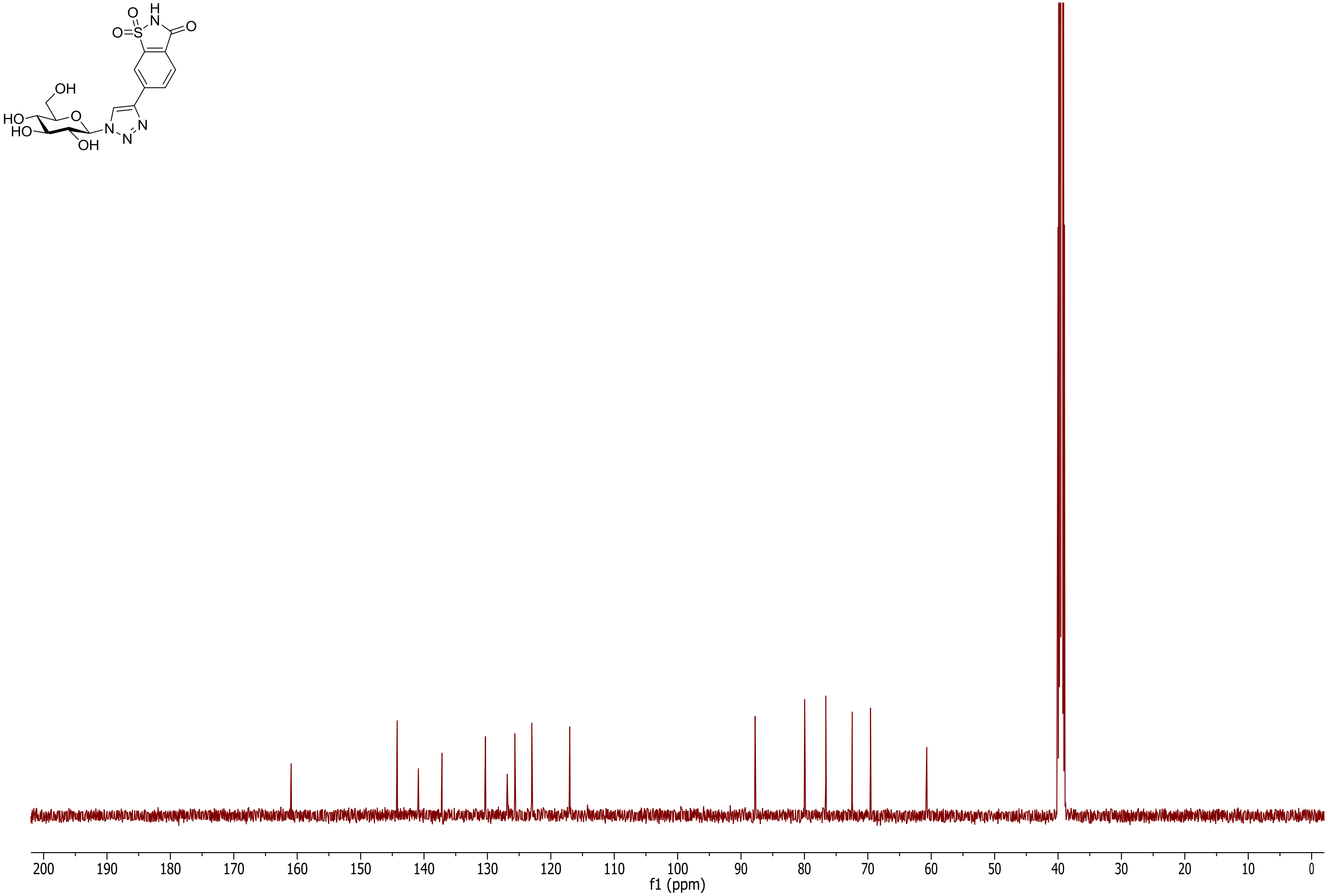

Compound **14** 500 MHz ^1H NMR $(CD_3)_2SO$

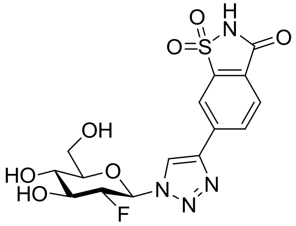

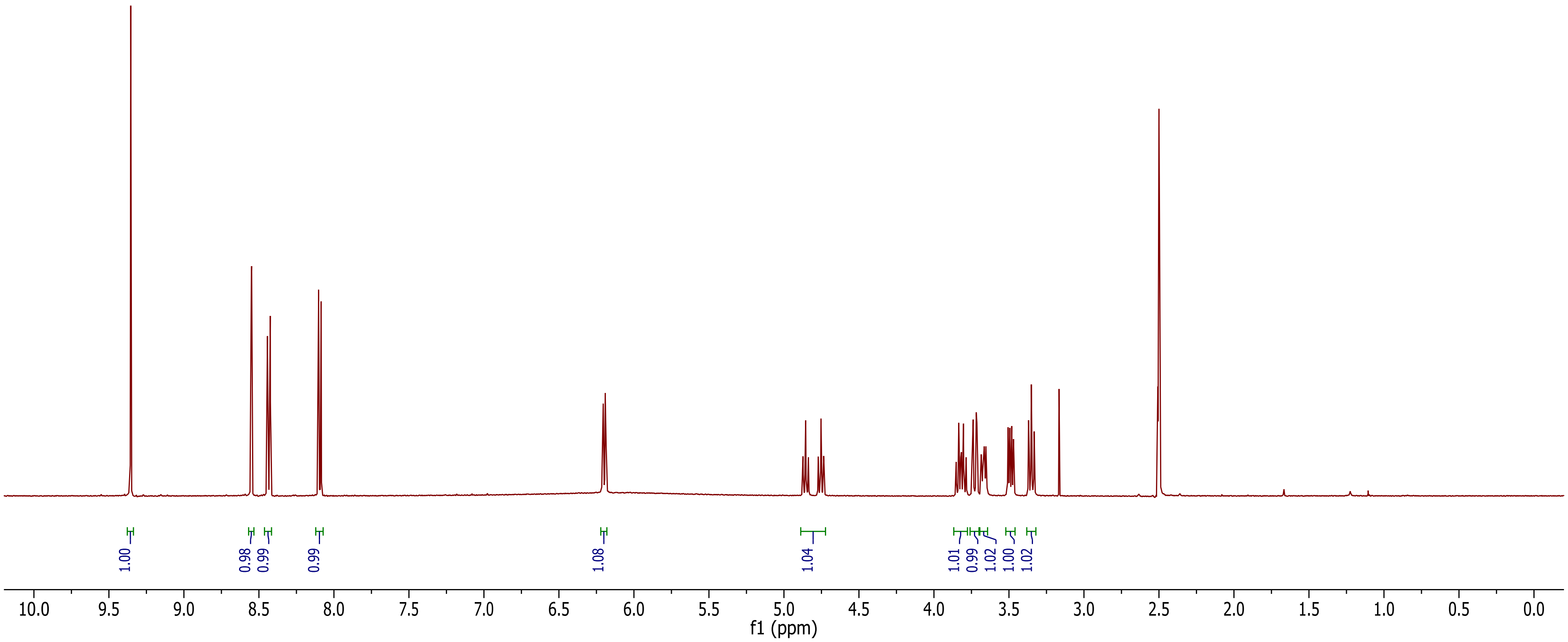

Compound **14** 125 MHz ^{13}C NMR $(CD_3)_2SO$

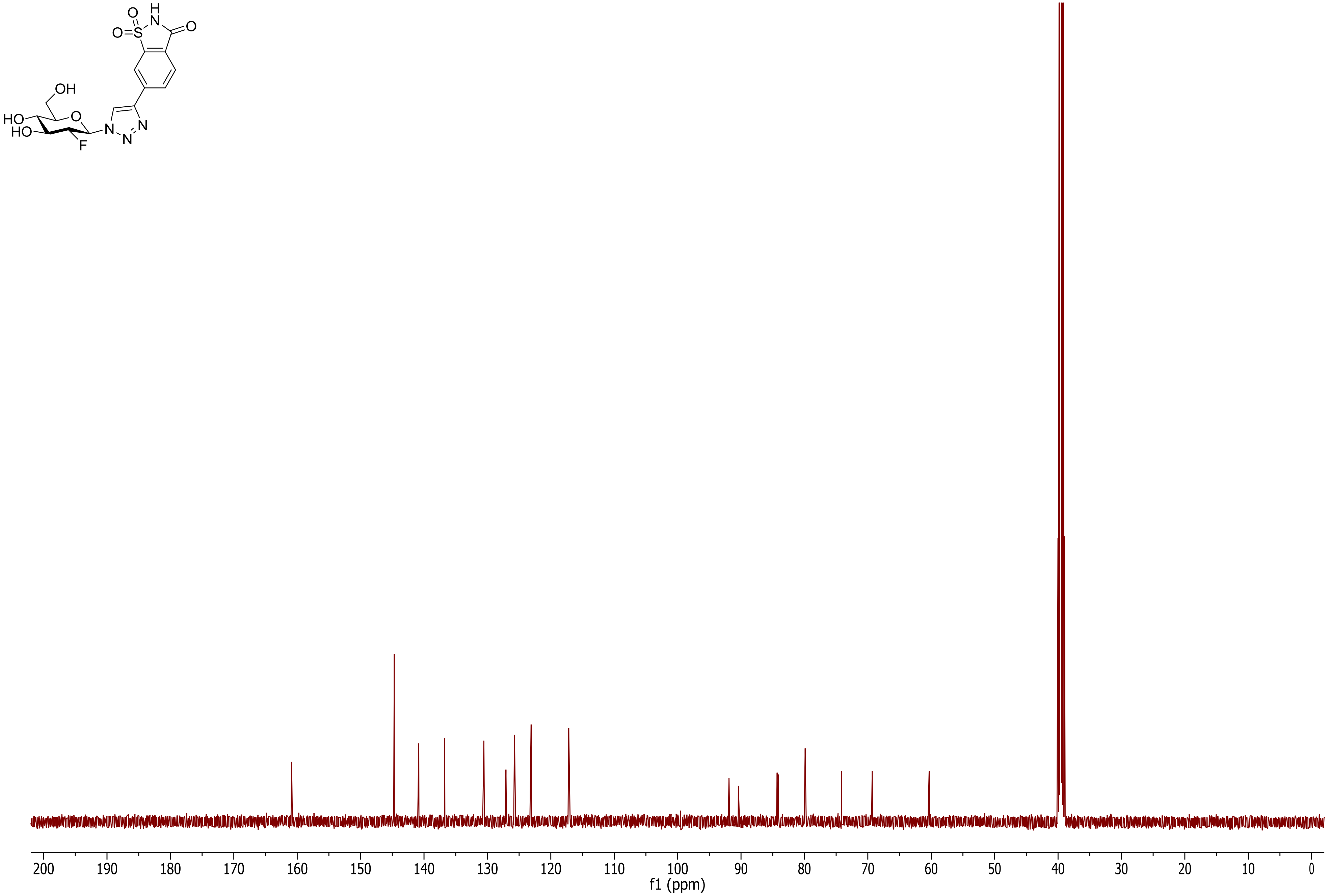

Compound **14** 376 MHz ^{19}F NMR $(CD_3)_2SO$

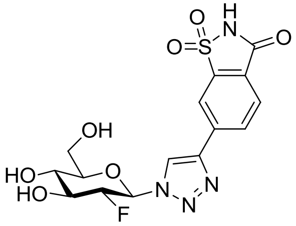

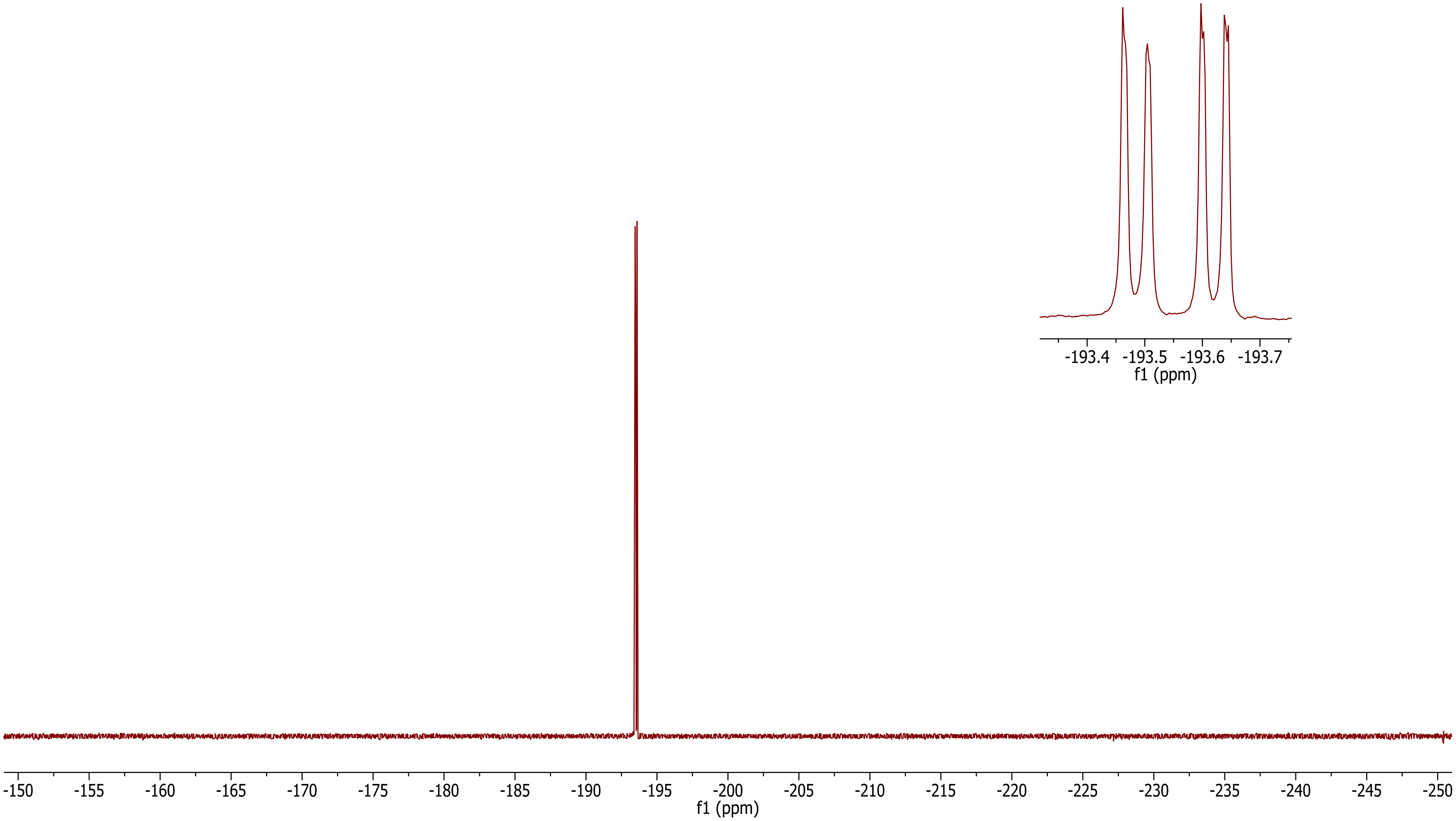

Compound **15** 500 MHz ^{1}H NMR $(CD_3)_2SO$

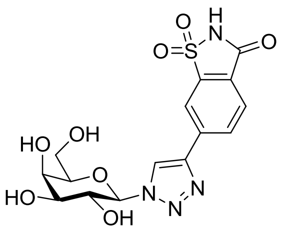

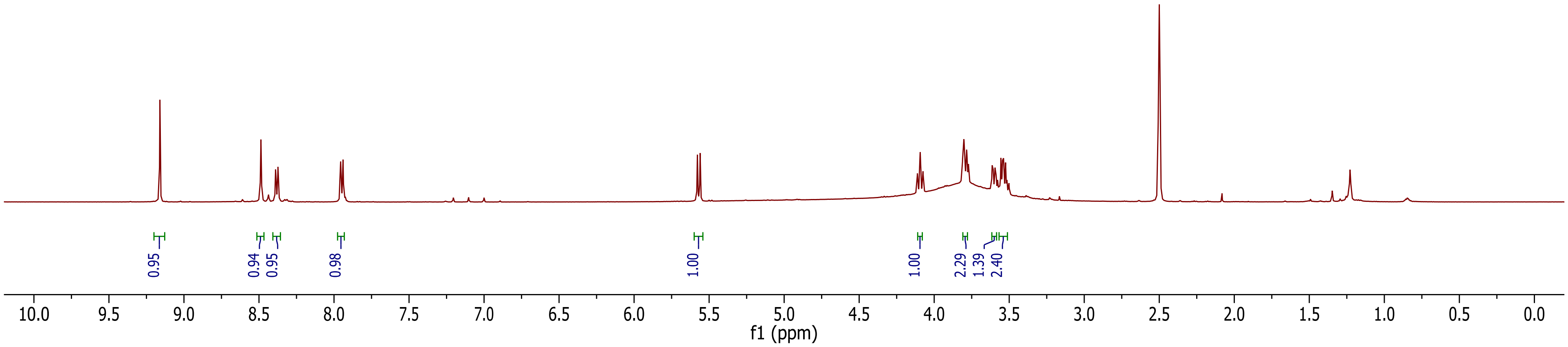

Compound **15** 125 MHz ^{13}C NMR $(CD_3)_2SO$

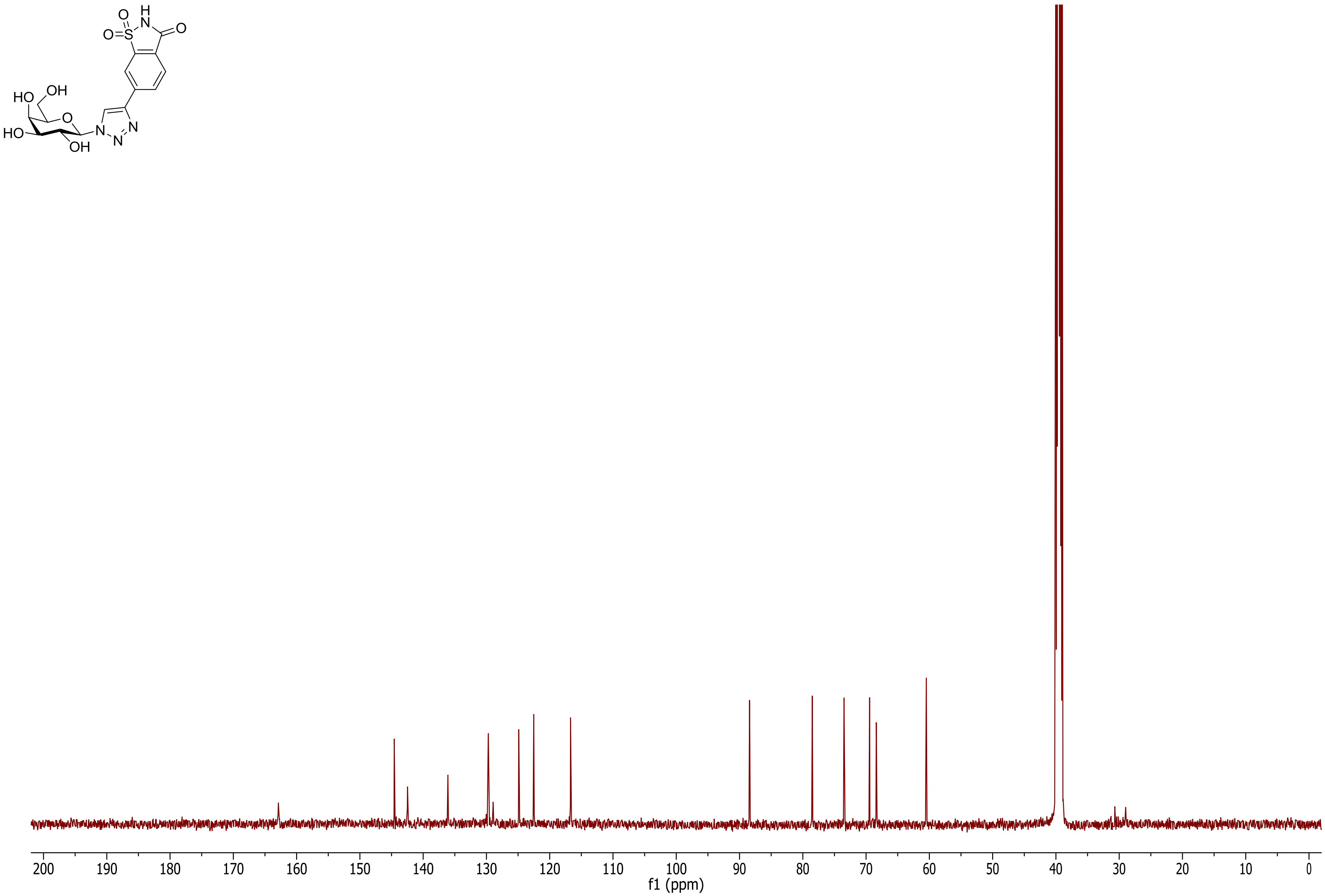

Compound **17** 500 MHz ^{1}H NMR $(CD_3)_2SO$

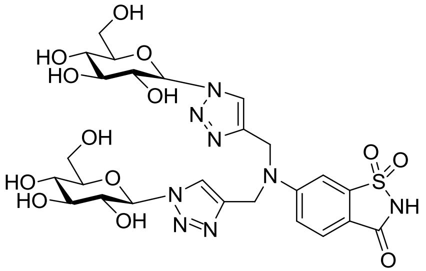

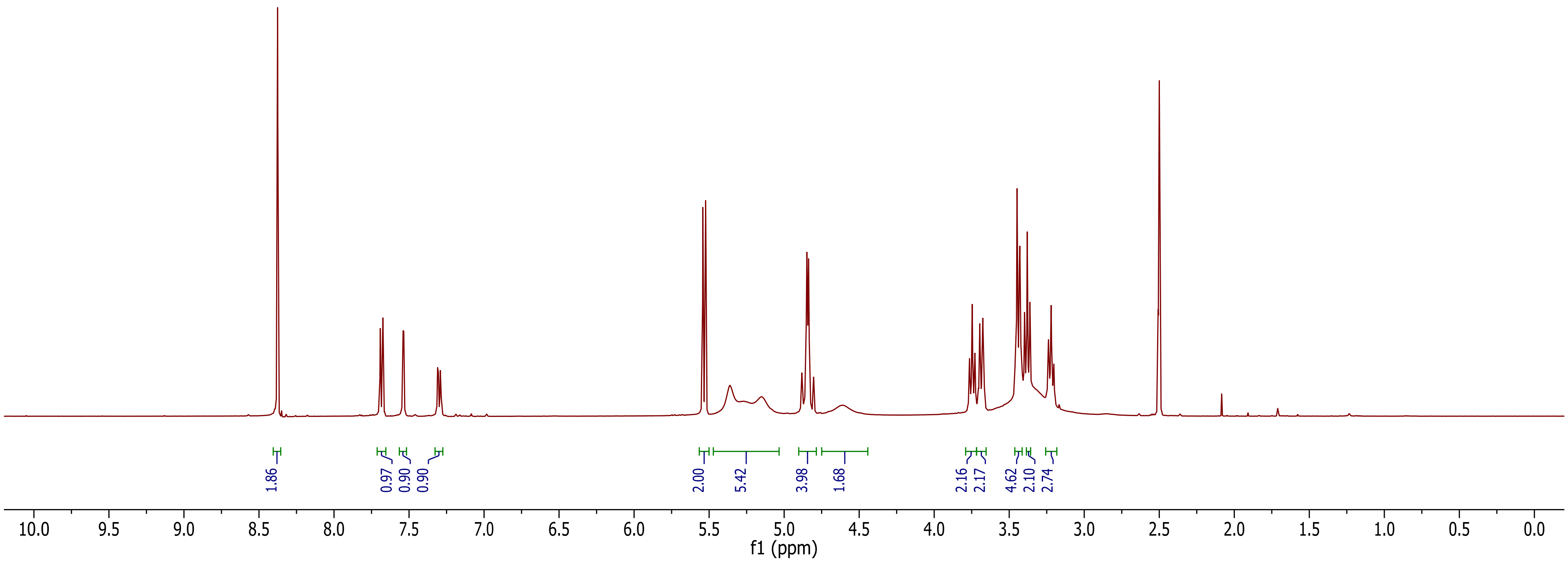

Compound **17** 125 MHz ^{13}C NMR $(CD_3)_2SO$

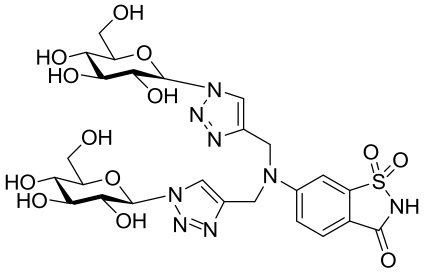

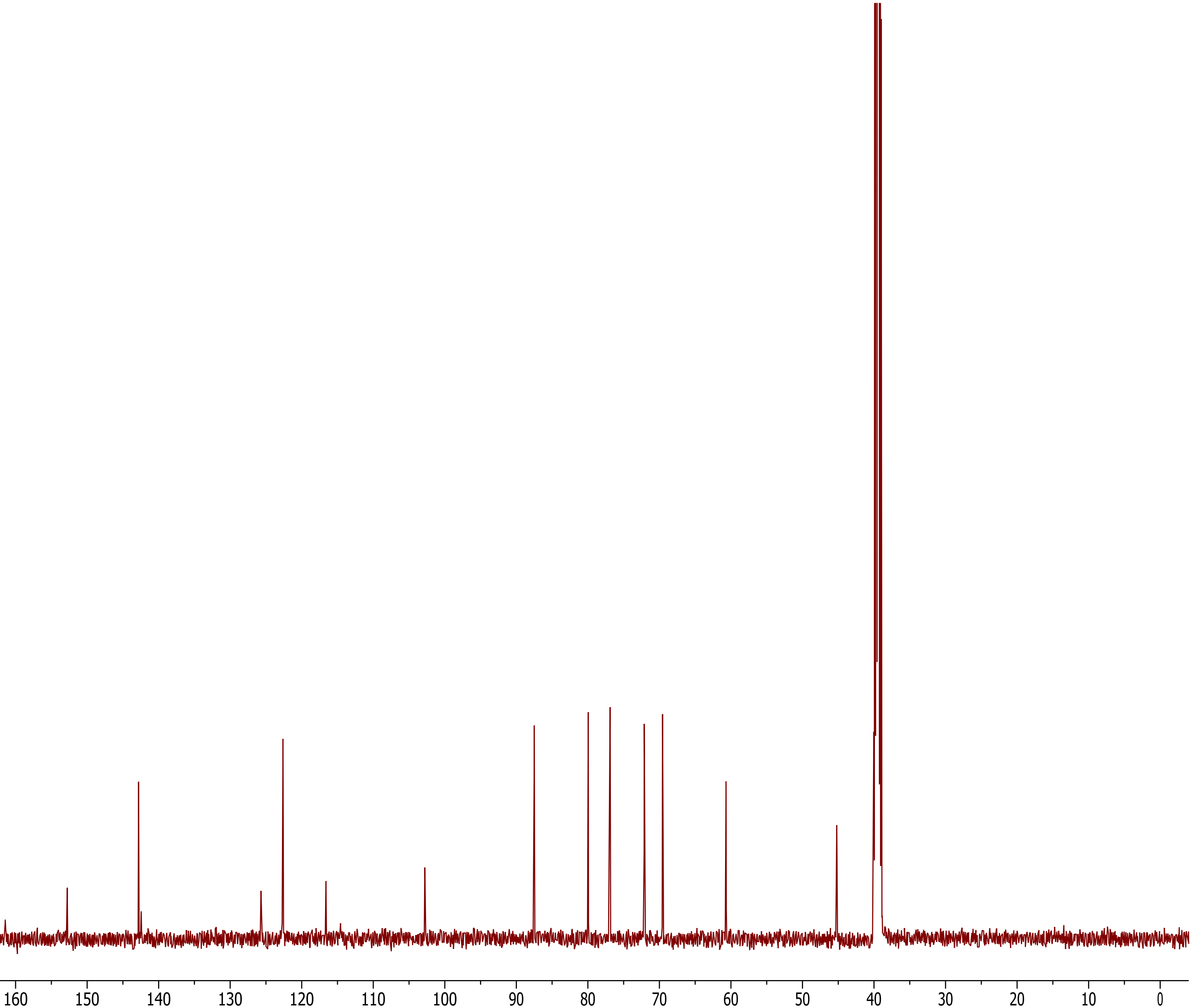

Compound **19** 500 MHz ^{1}H NMR $(CD_3)_2SO$

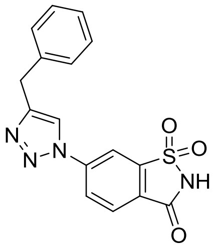

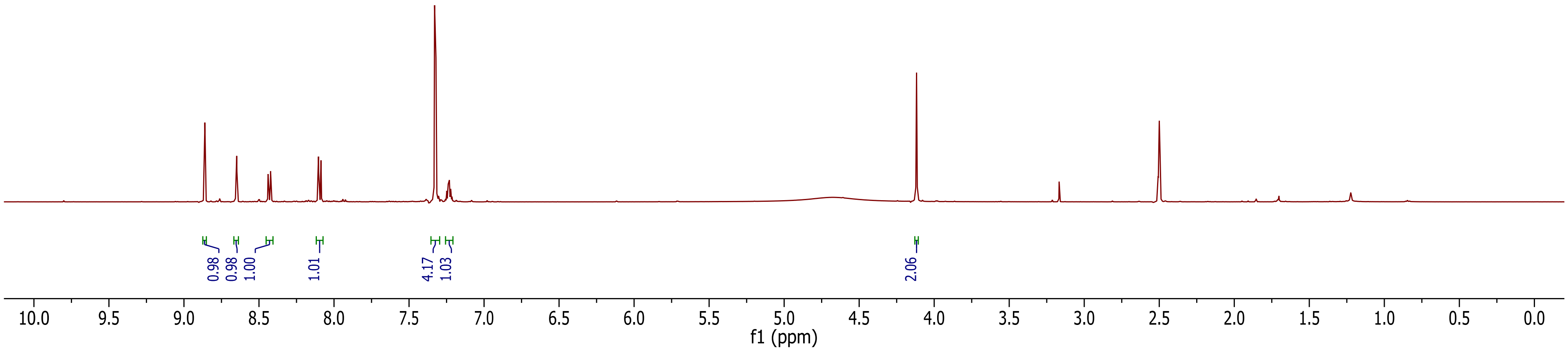

Compound **19** 125 MHz ^{13}C NMR $(CD_3)_2SO$

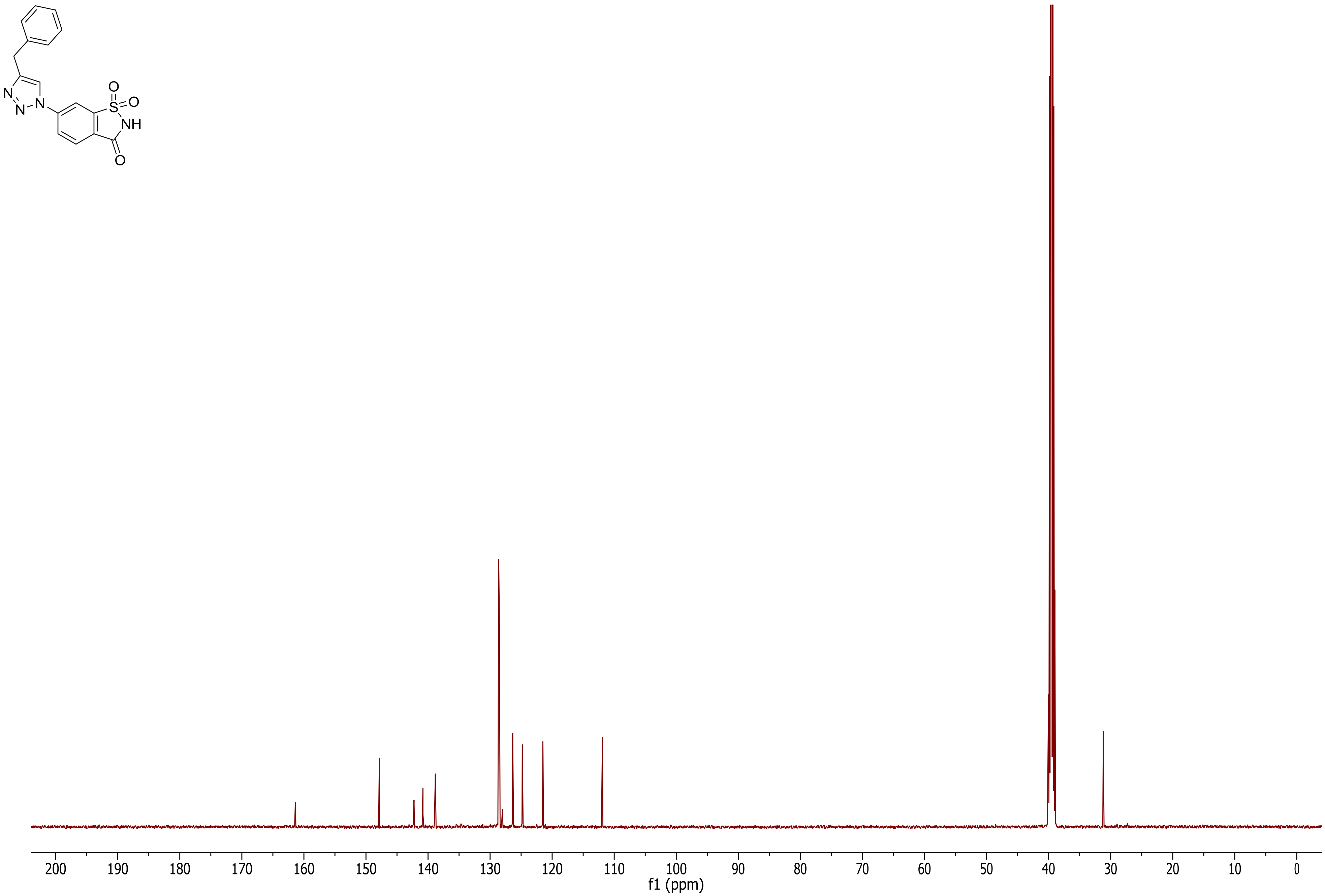

Compound **20** 500 MHz ^{1}H NMR $(CD_3)_2SO$

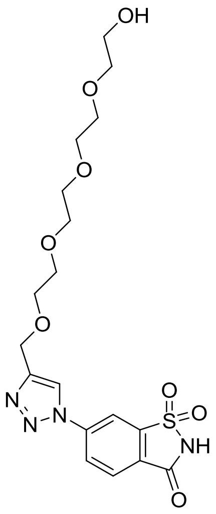

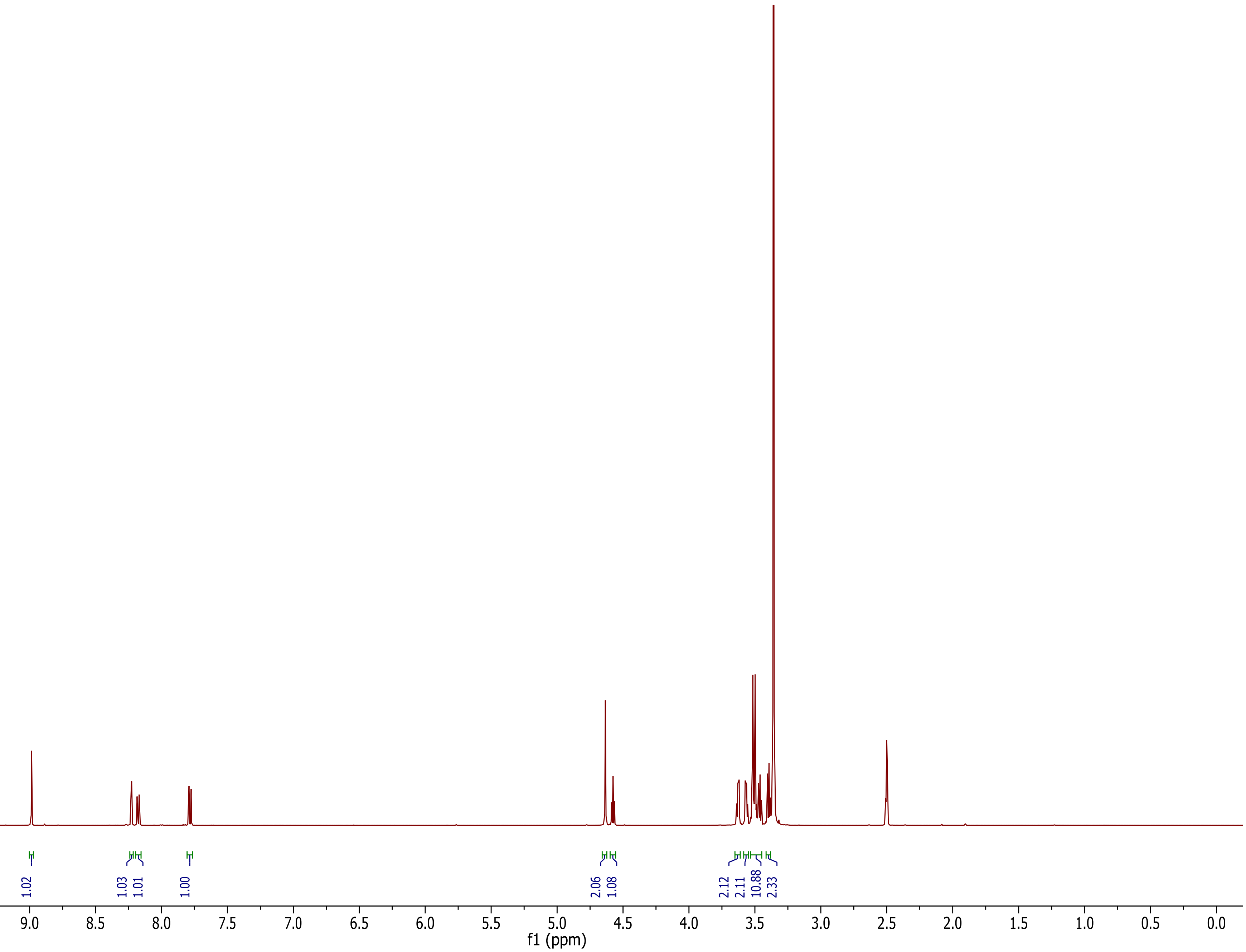

Compound **20** 125 MHz ^{13}C NMR $(CD_3)_2SO$

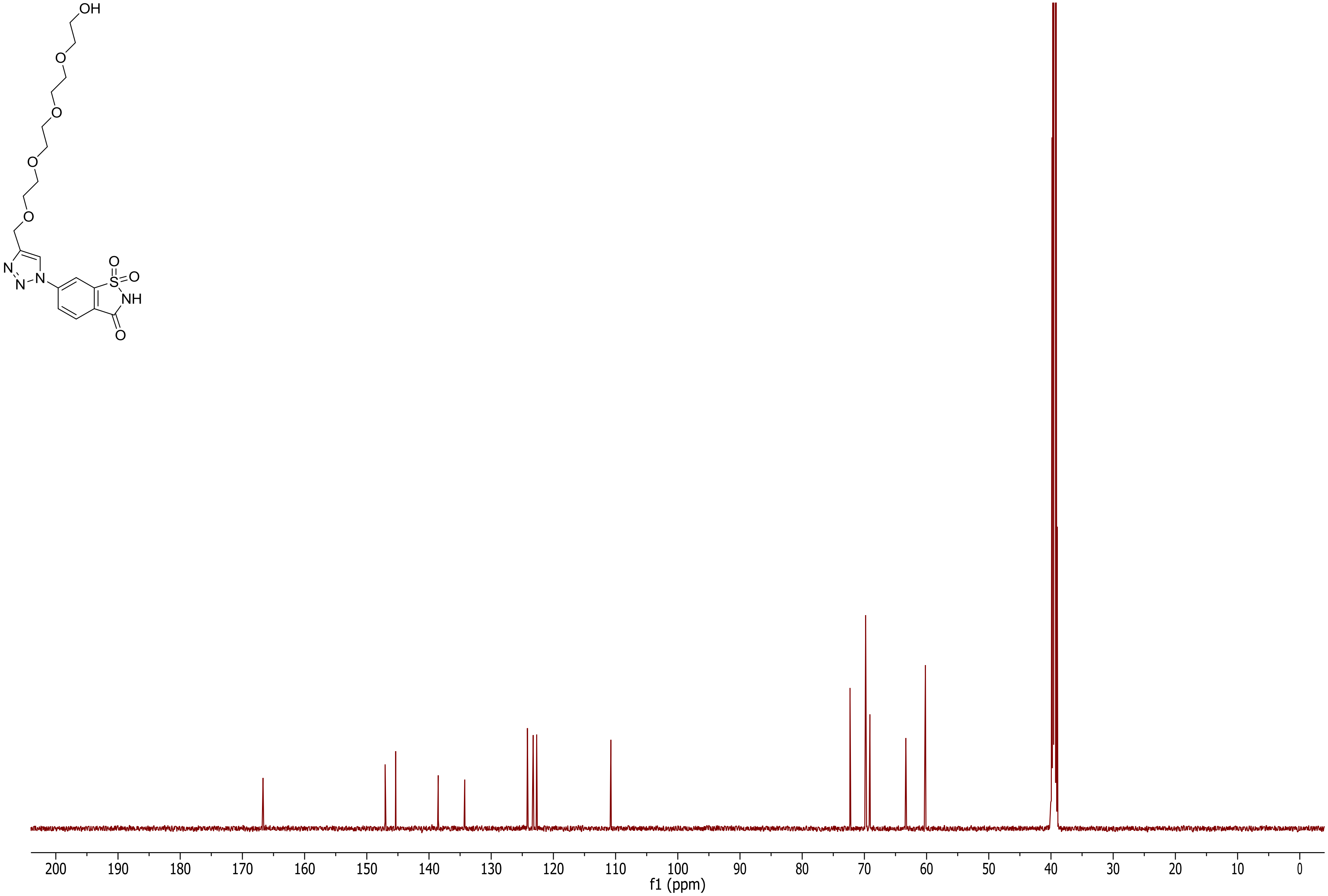

Compound **21** 500 MHz ^{1}H NMR $(CD_3)_2SO$

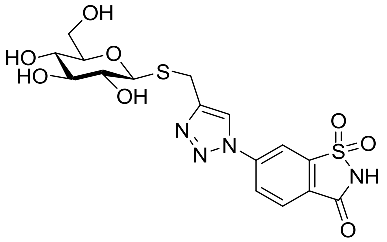

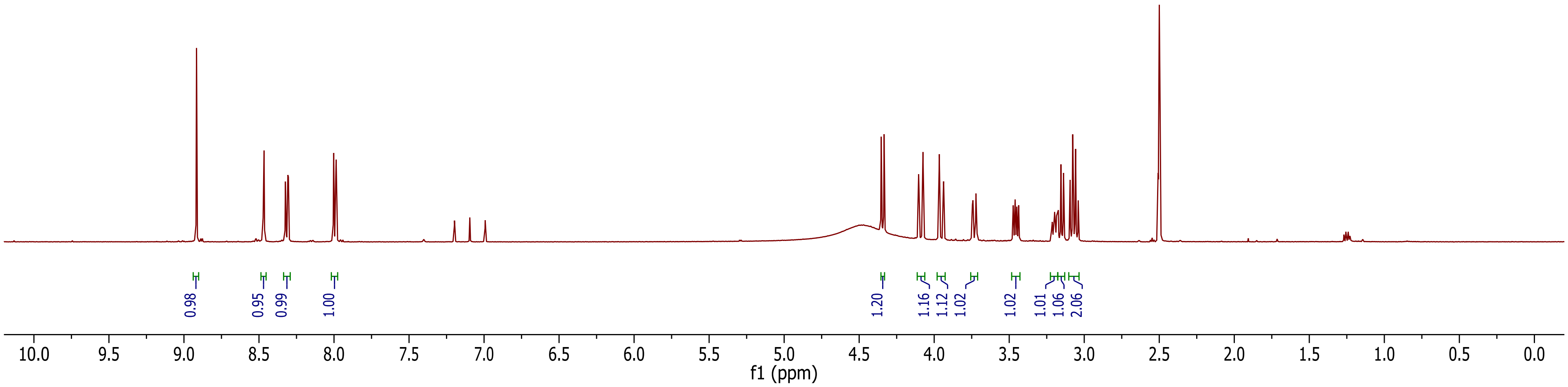

Compound **21** 125 MHz ^{13}C NMR $(CD_3)_2SO$

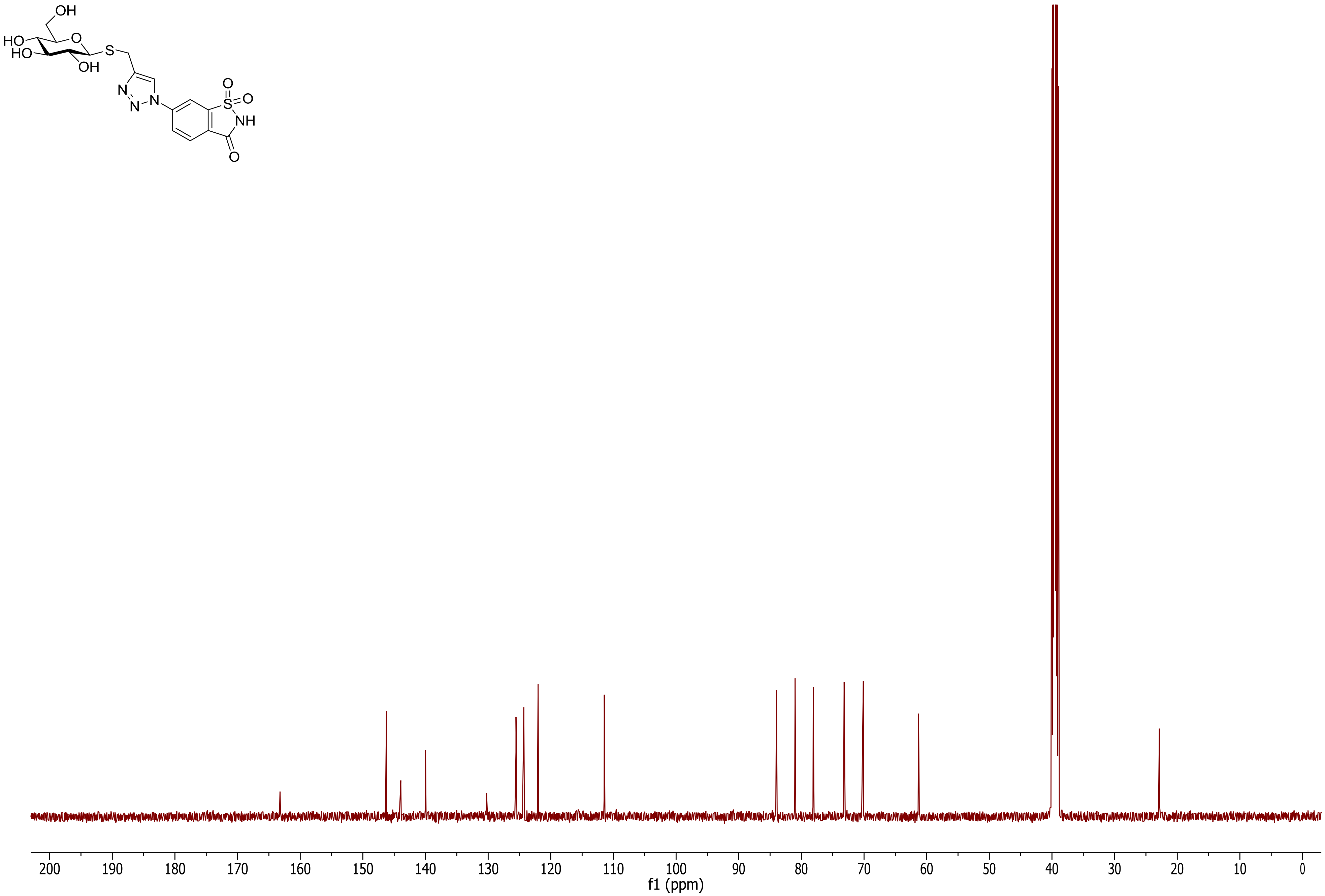

Compound **22** 500 MHz ^{1}H NMR $(CD_3)_2SO$

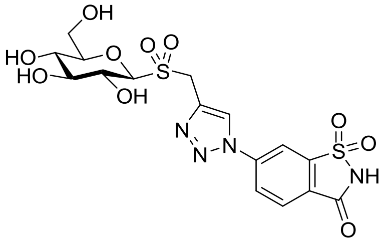

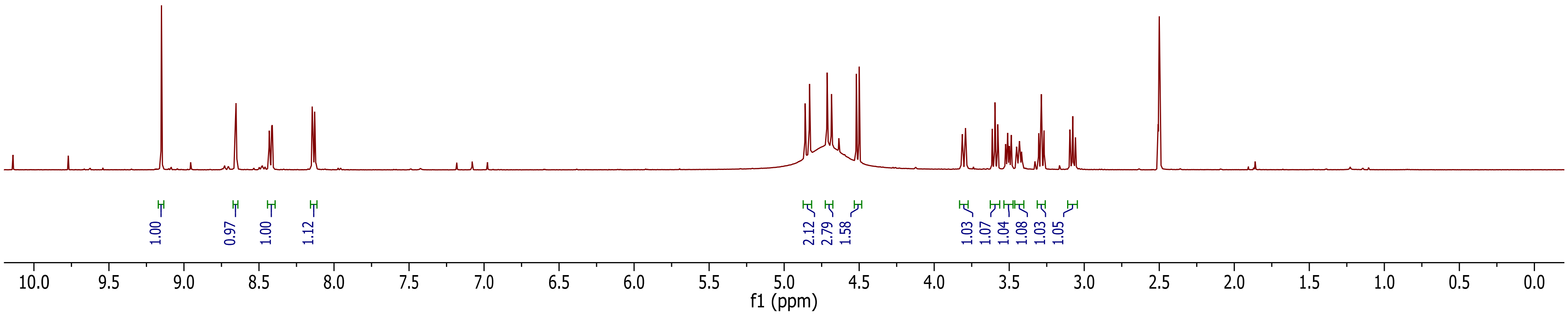

Compound **22** 125 MHz ^{13}C NMR $(CD_3)_2SO$

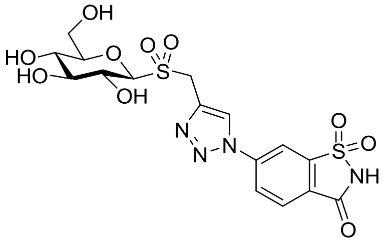

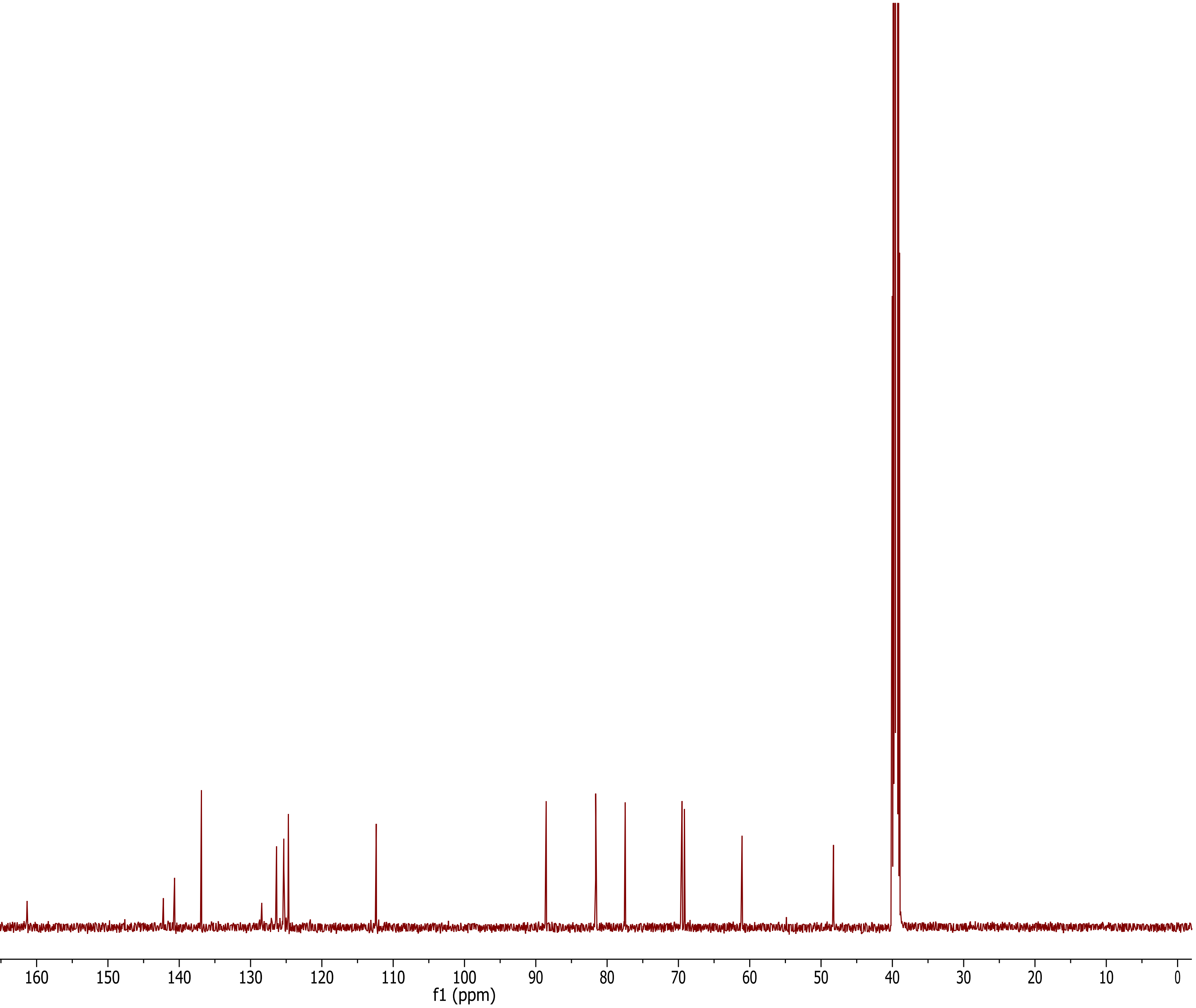

Compound **24** 500 MHz ^{1}H NMR $(CD_3)_2SO$

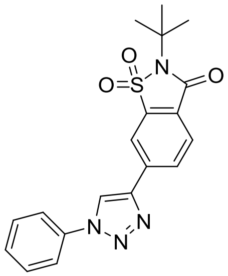

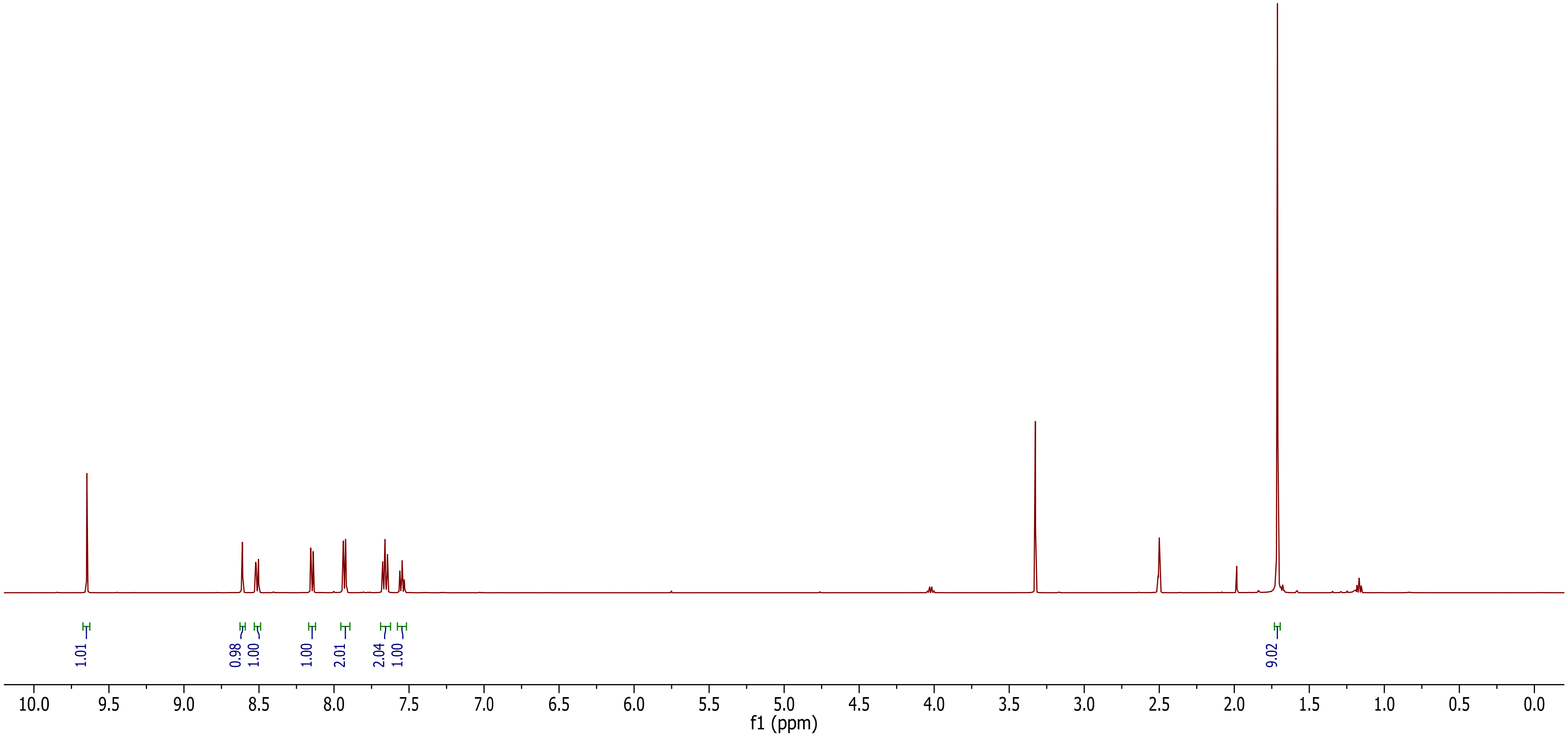

Compound **24** 125 MHz ^{13}C NMR $(CD_3)_2SO$

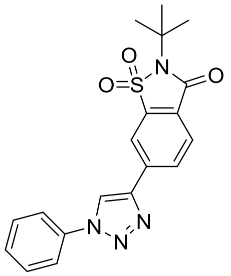

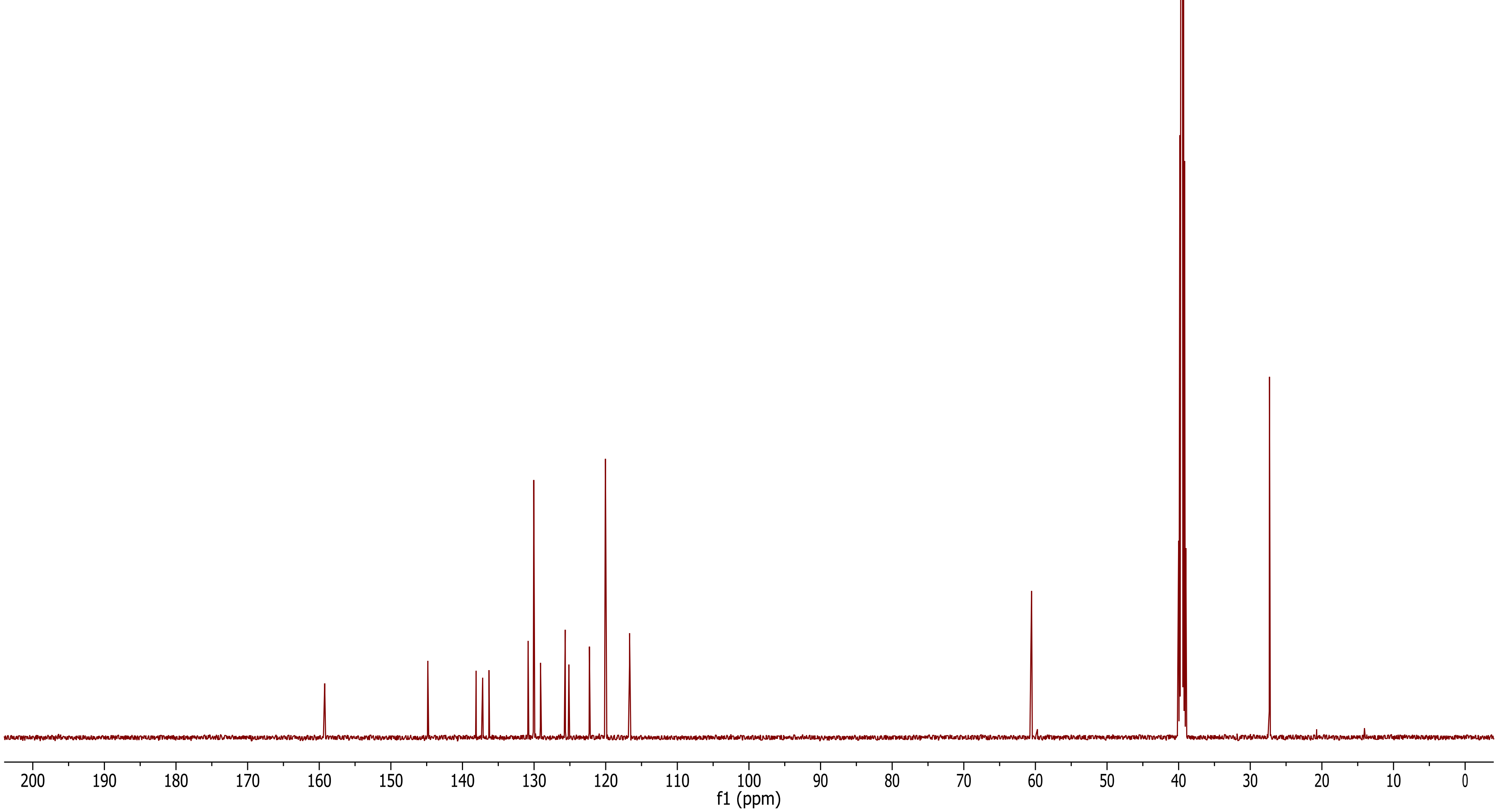

Compound **25** 500 MHz ^{1}H NMR $(CD_3)_2SO$

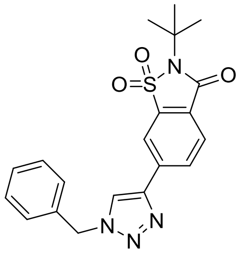

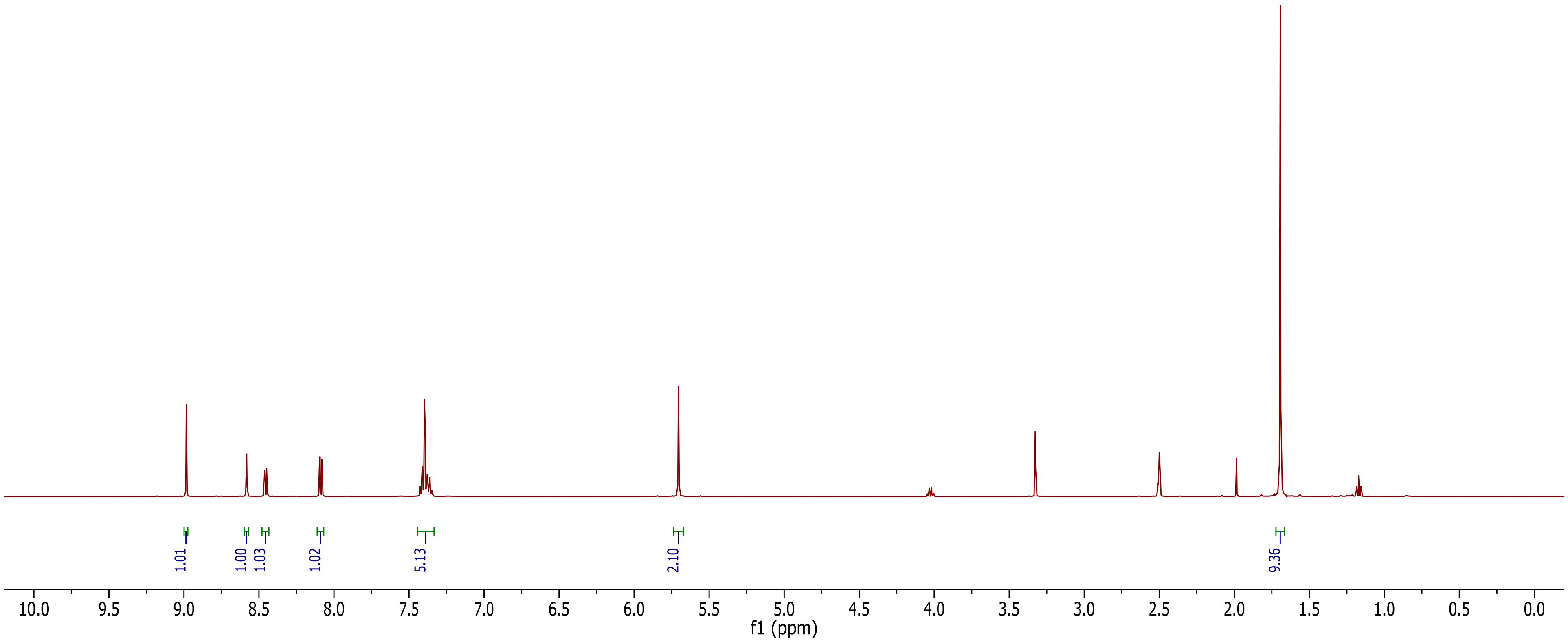

Compound **25** 125 MHz ^{13}C NMR $(CD_3)_2SO$

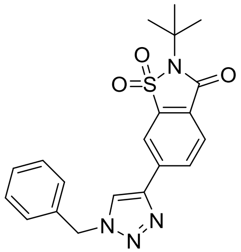

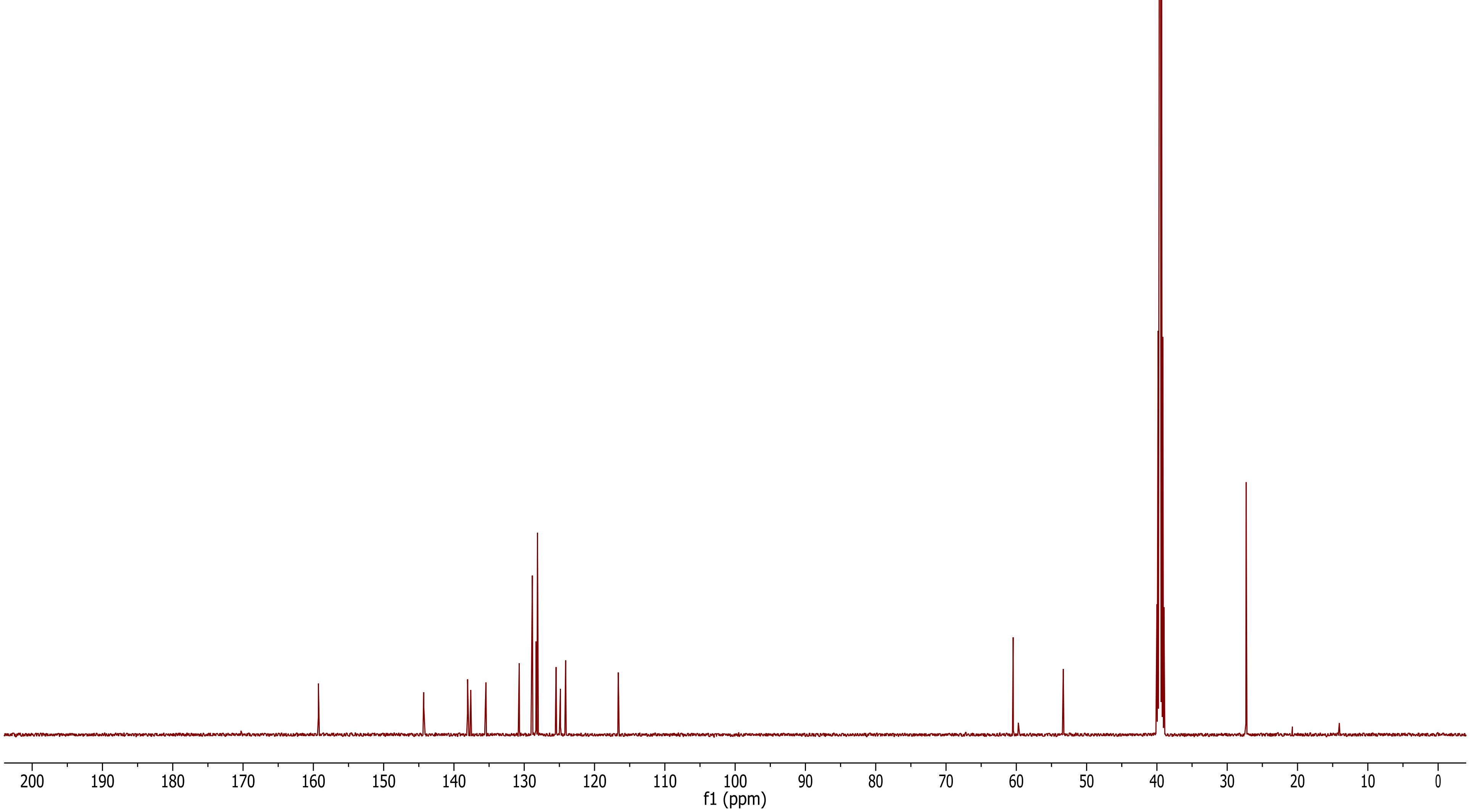

Compound **26** 500 MHz ^{1}H NMR $(CD_3)_2SO$

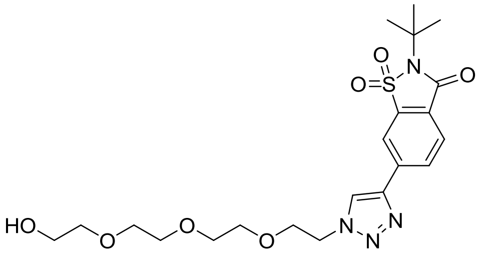

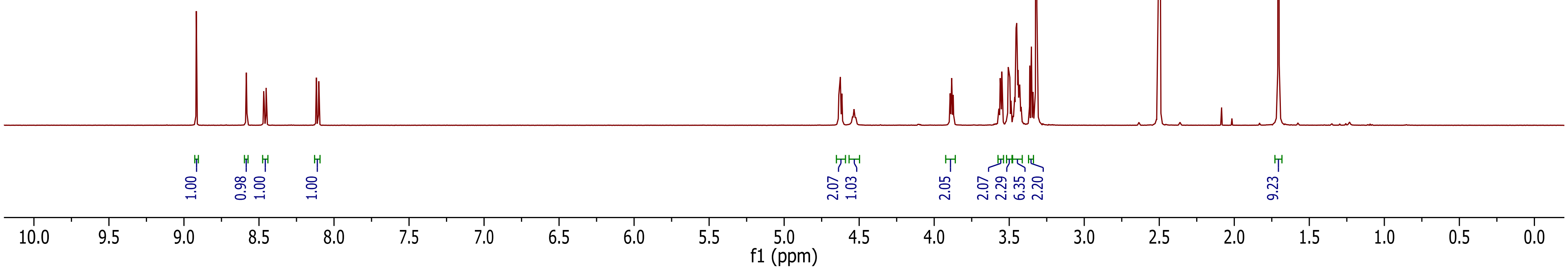

Compound **26** 125 MHz ^{13}C NMR $(CD_3)_2SO$

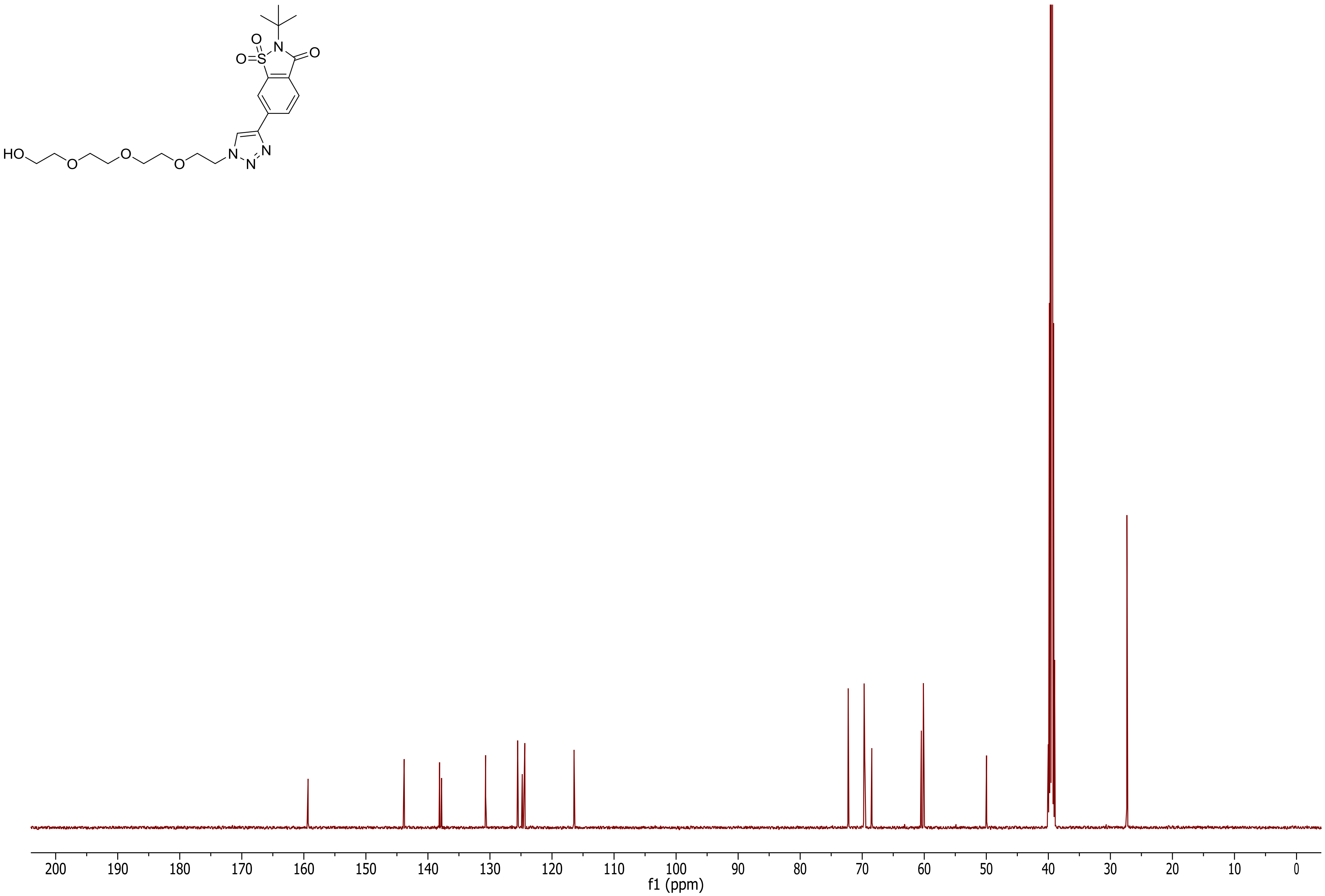

Compound **27** 500 MHz ^{1}H NMR $CDCl_3$

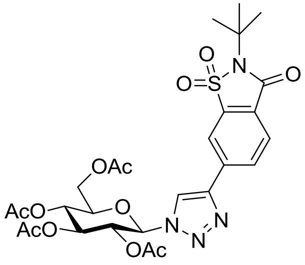

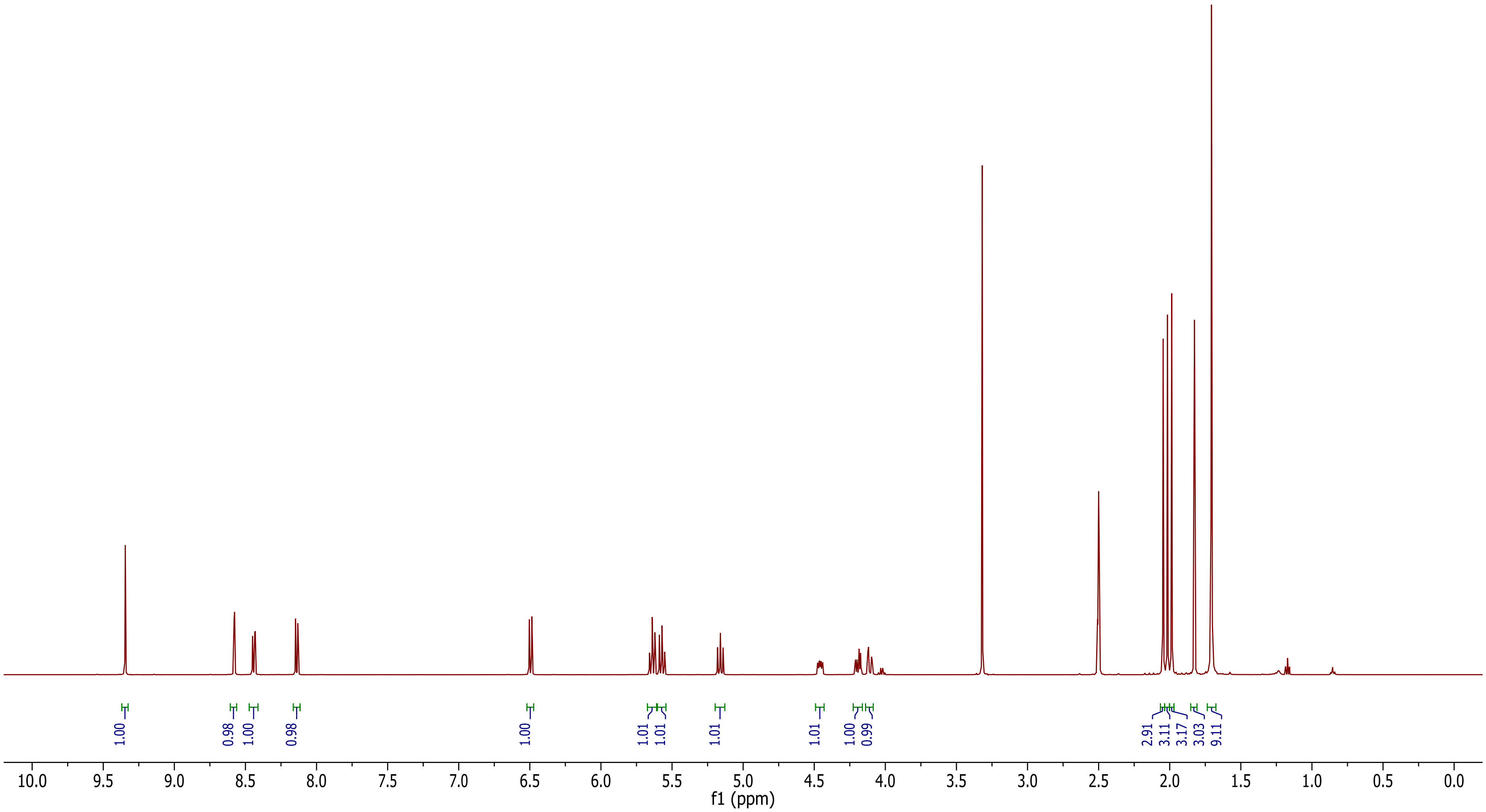

Compound **27** 125 MHz ^{13}C NMR $CDCl_3$

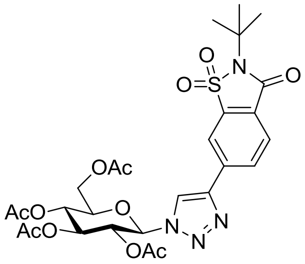

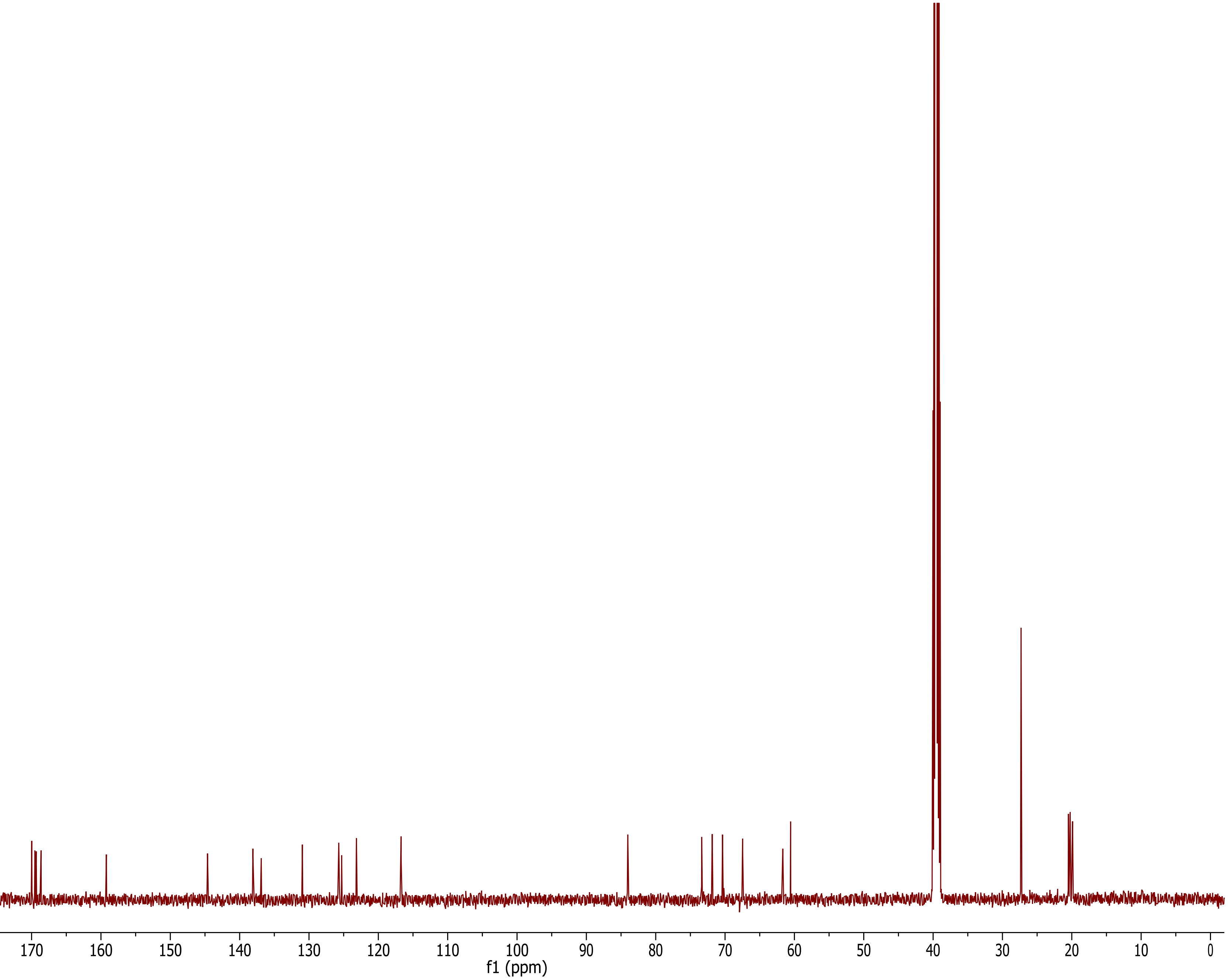

Compound **28** 500 MHz ^{1}H NMR $(CD_3)_2SO$

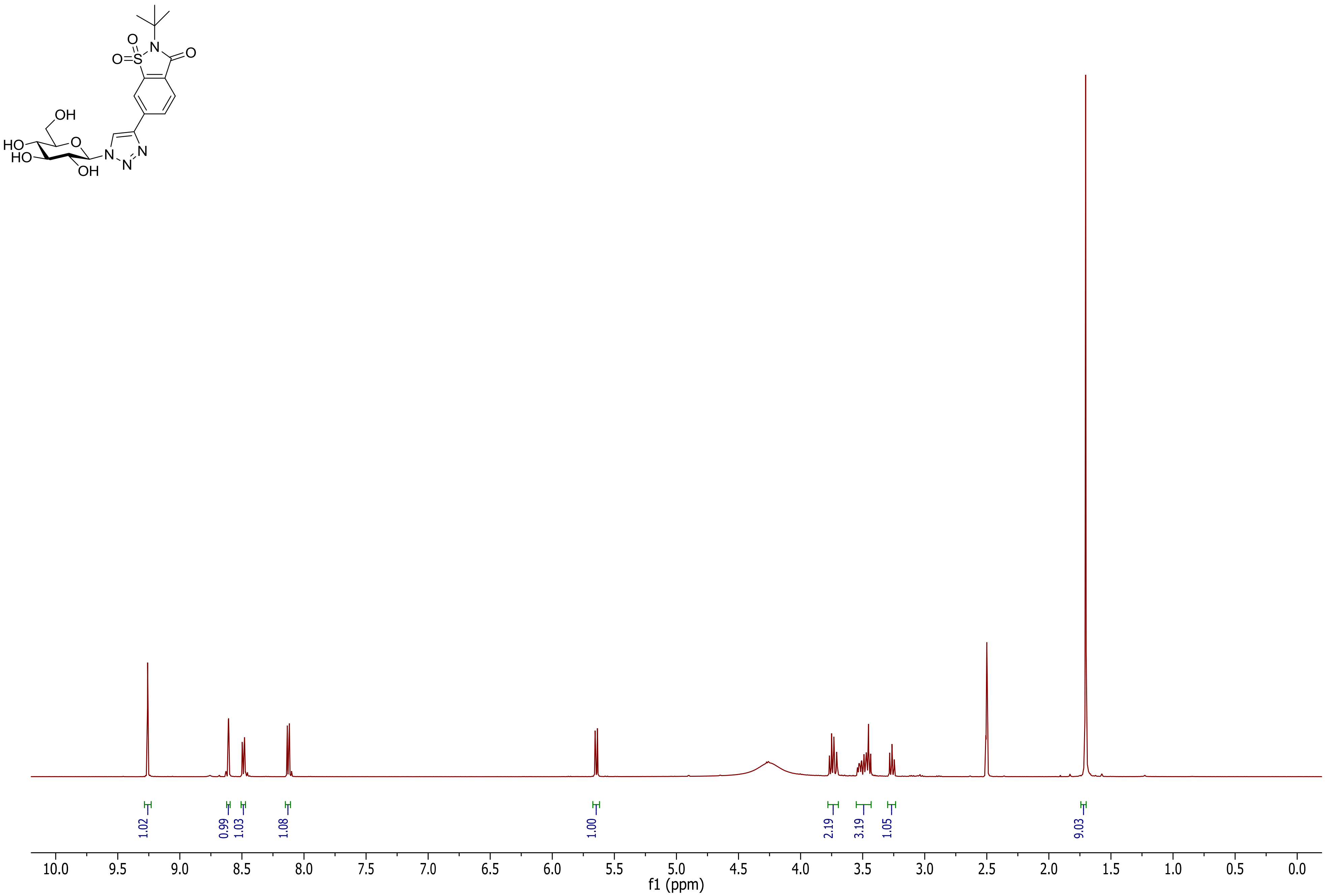

Compound **28** 125 MHz ^{13}C NMR $(CD_3)_2SO$

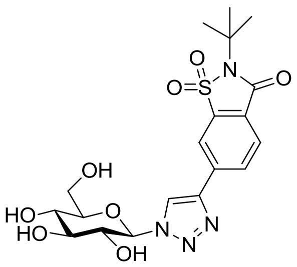

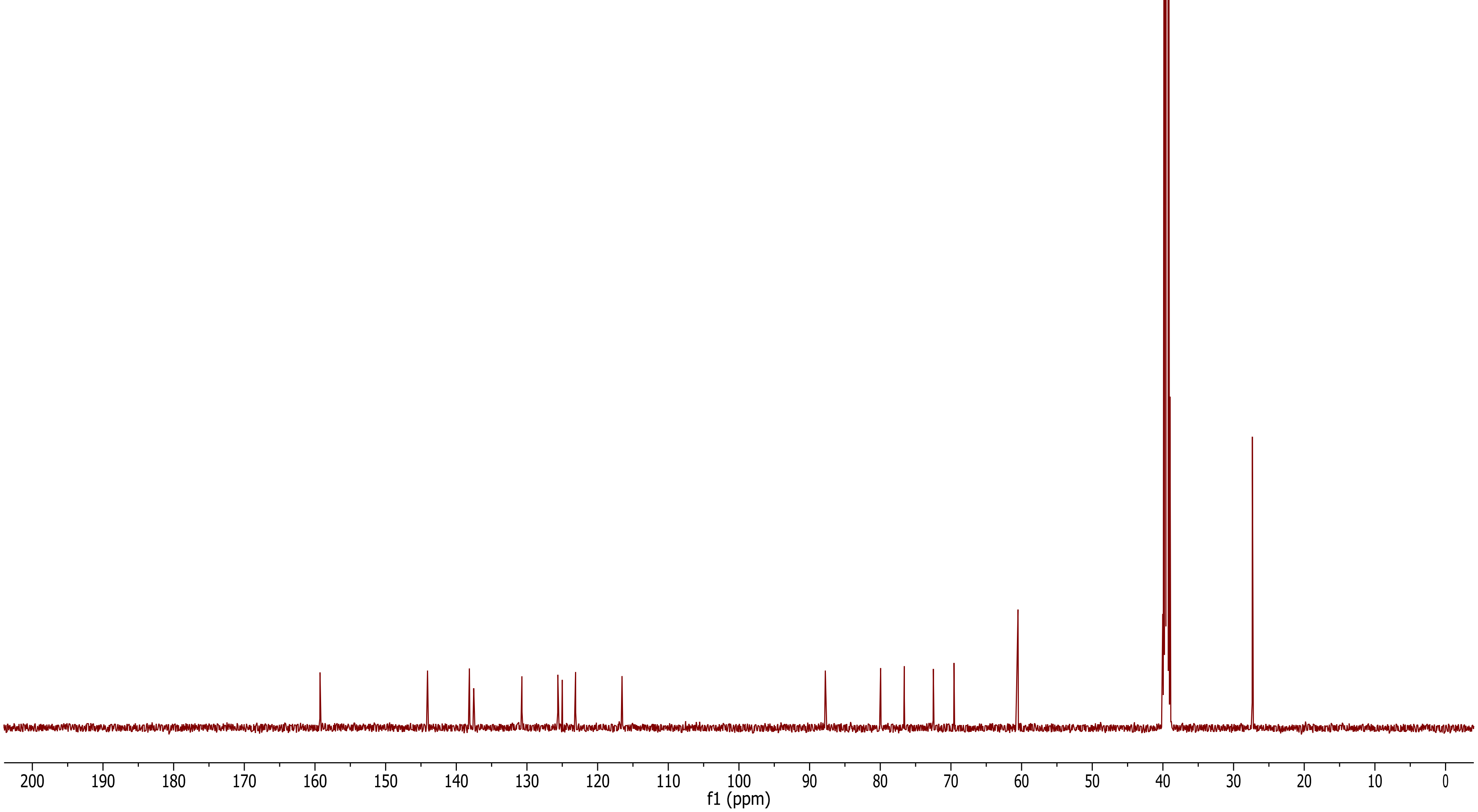

Compound **29** 500 MHz ^{1}H NMR $(CD_3)_2SO$

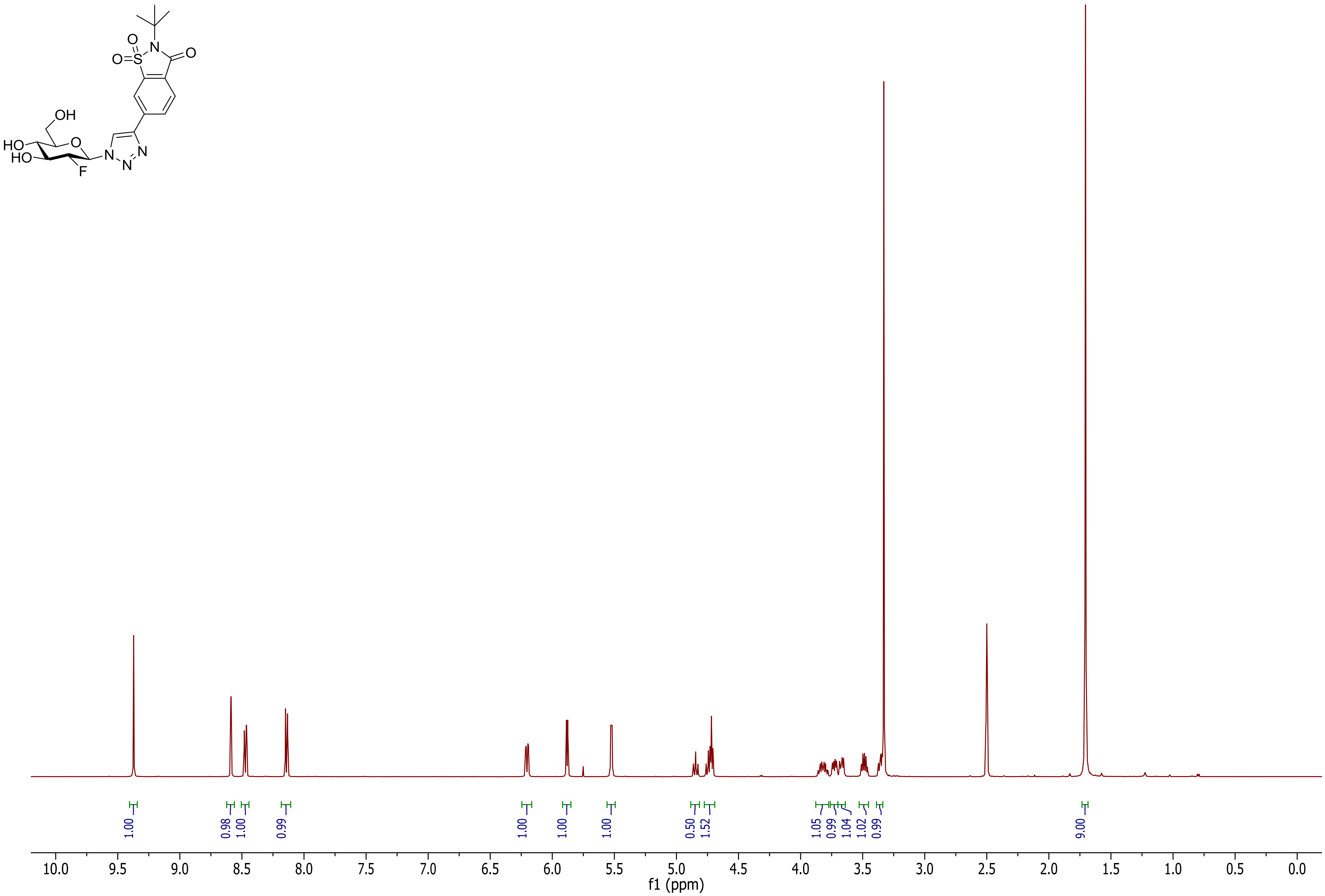

Compound **29** 125 MHz ^{13}C NMR $(CD_3)_2SO$

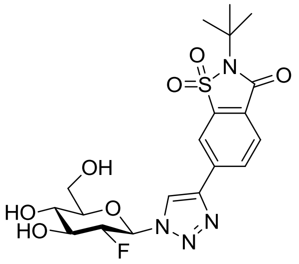

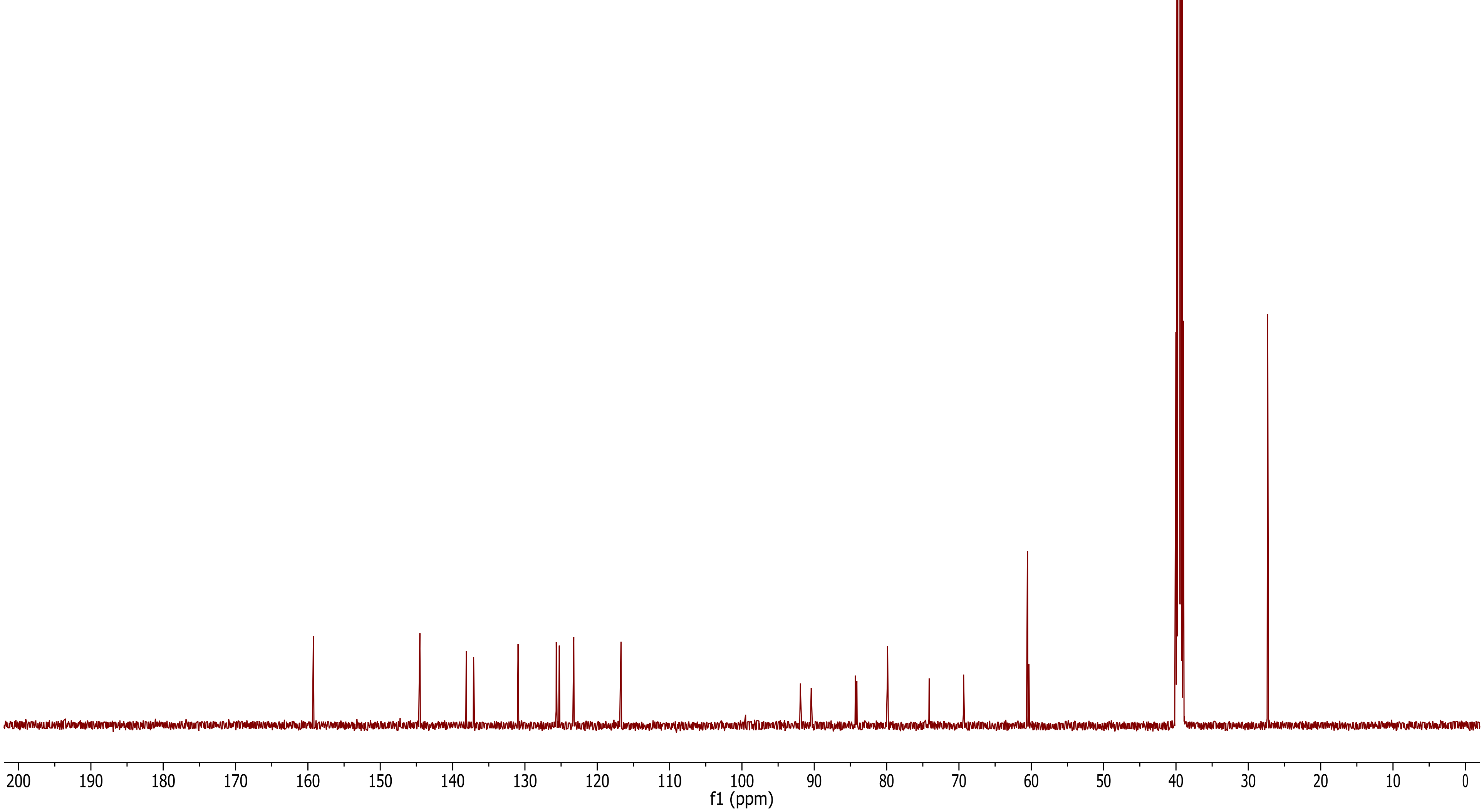

Compound **29** 376 MHz ^{19}F NMR $(CD_3)_2SO$

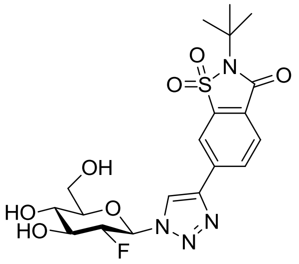

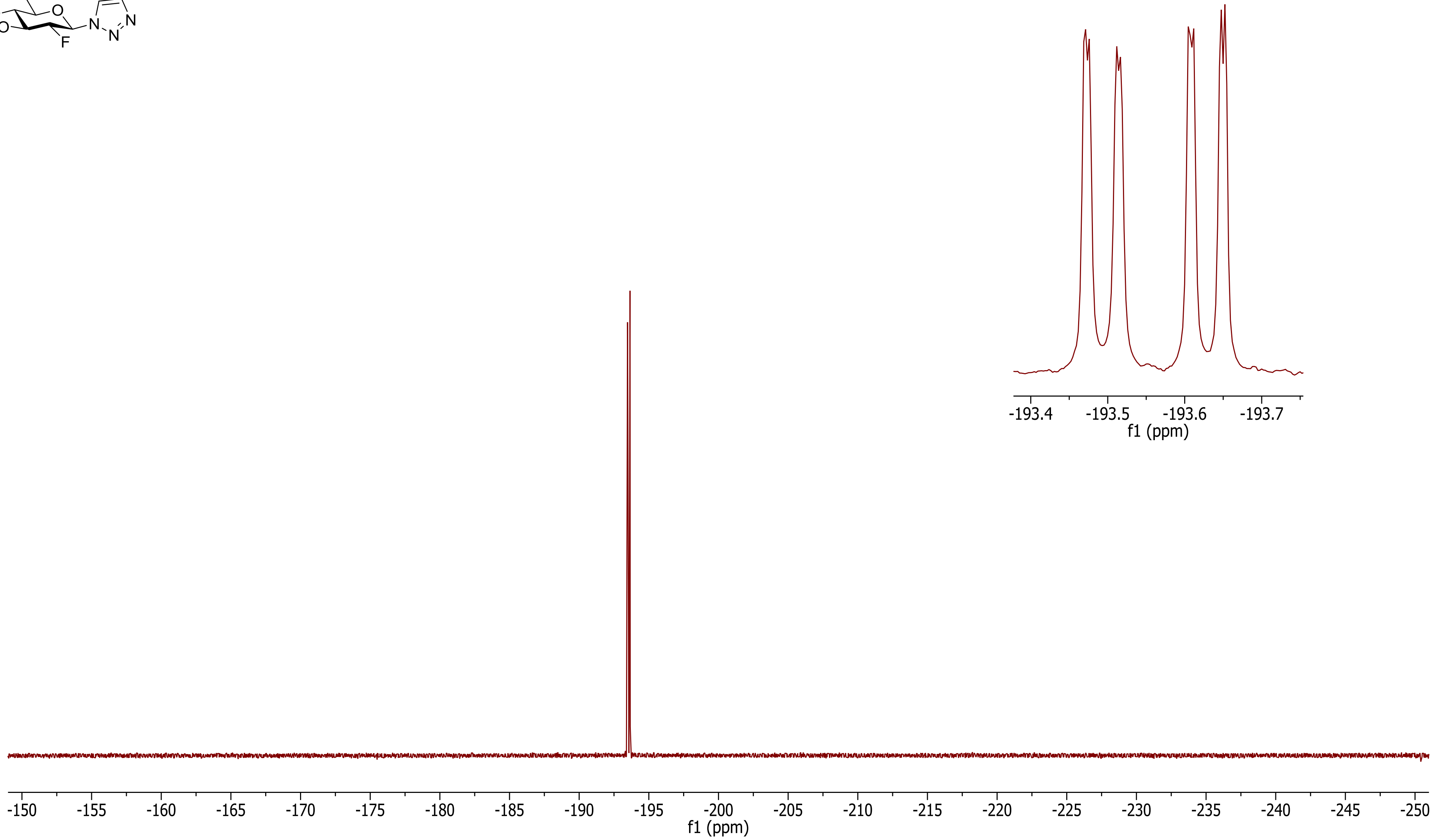

Compound **30** 500 MHz ^{1}H NMR $(CD_3)_2SO$

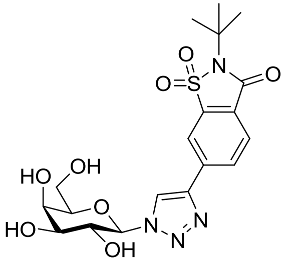

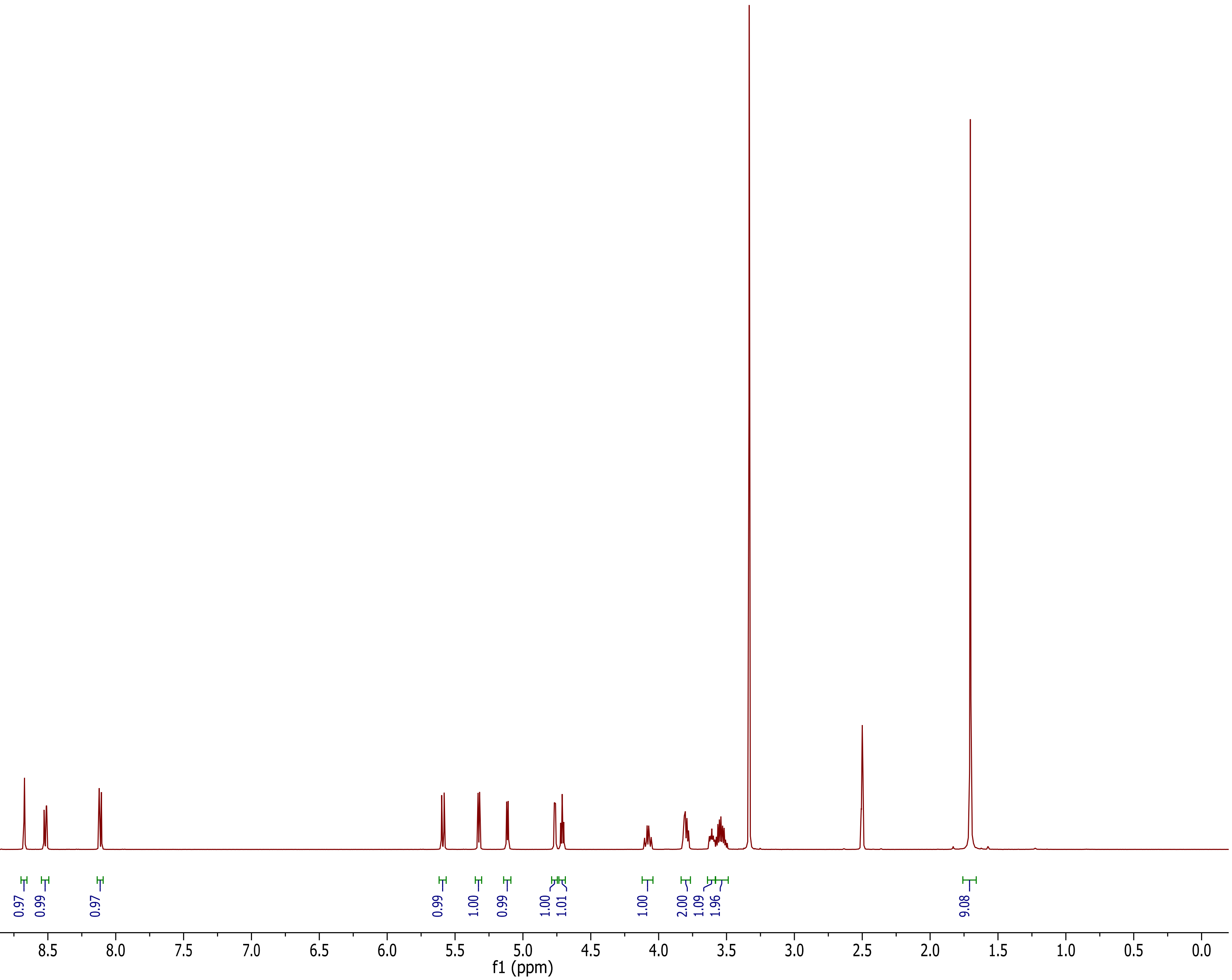

Compound **30** 125 MHz ^{13}C NMR $(CD_3)_2SO$

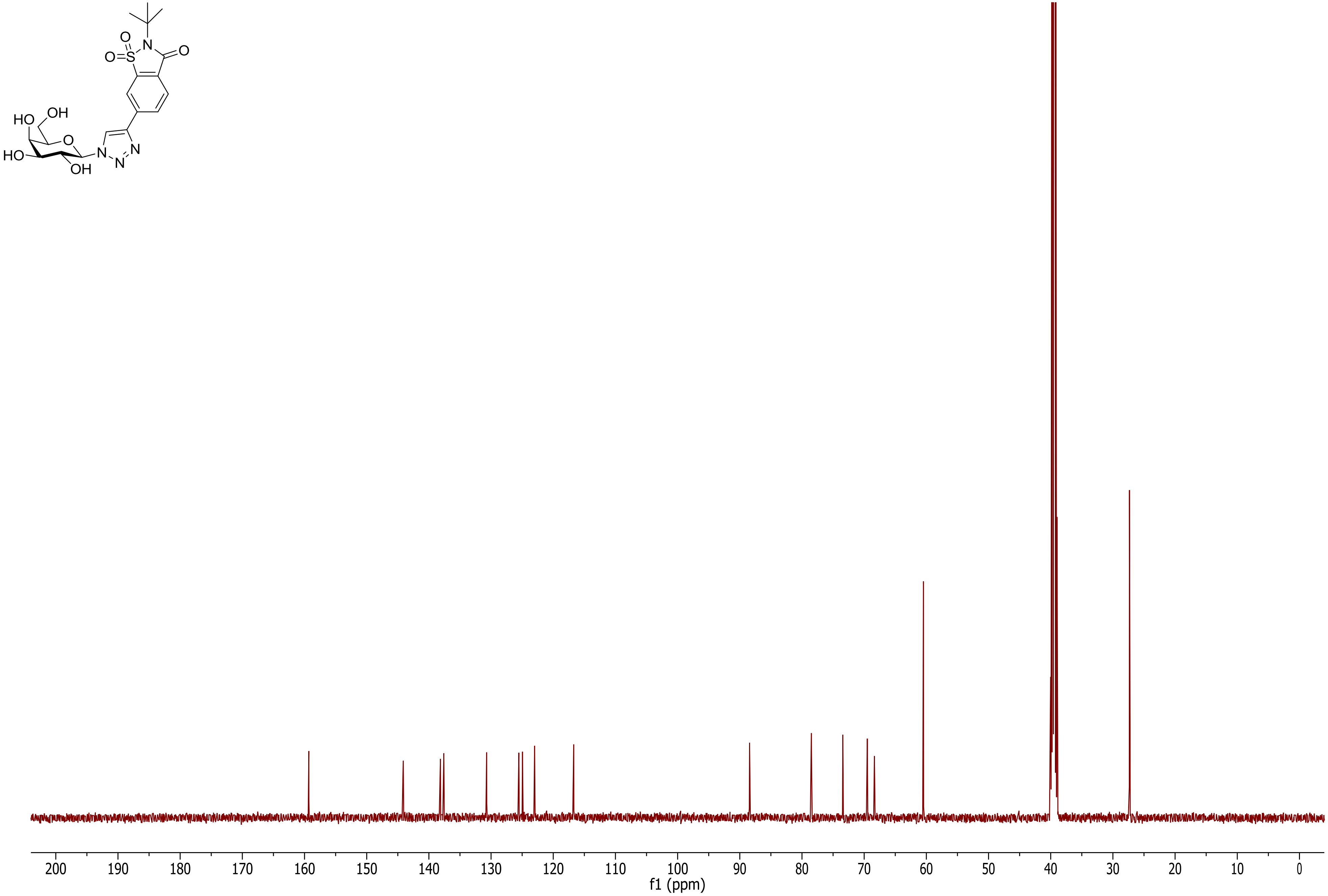

Compound **31** 500 MHz ^{1}H NMR $(CD_3)_2SO$

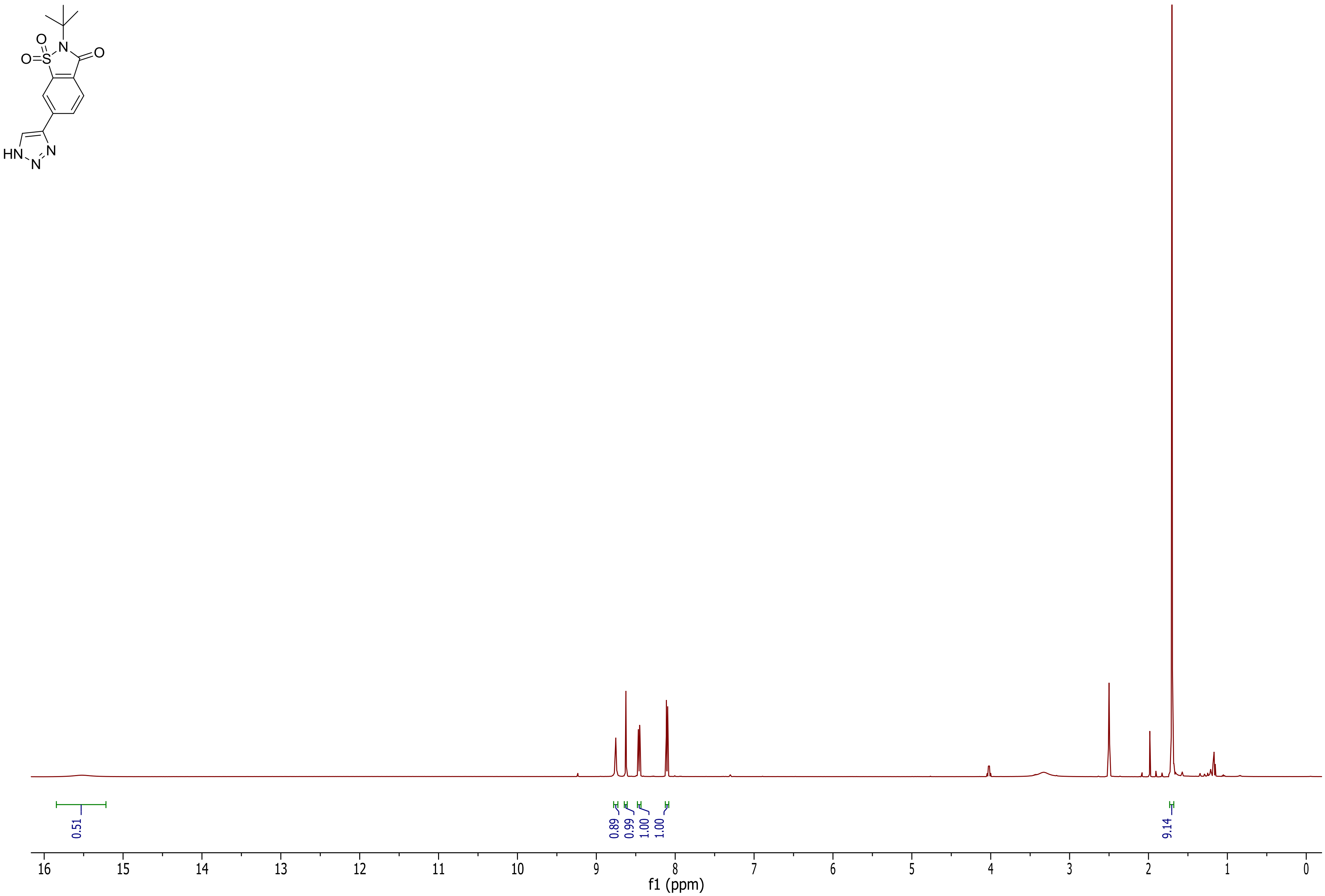

Compound **31** 125 MHz ^{13}C NMR $(CD_3)_2SO$

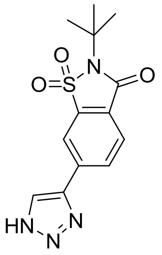

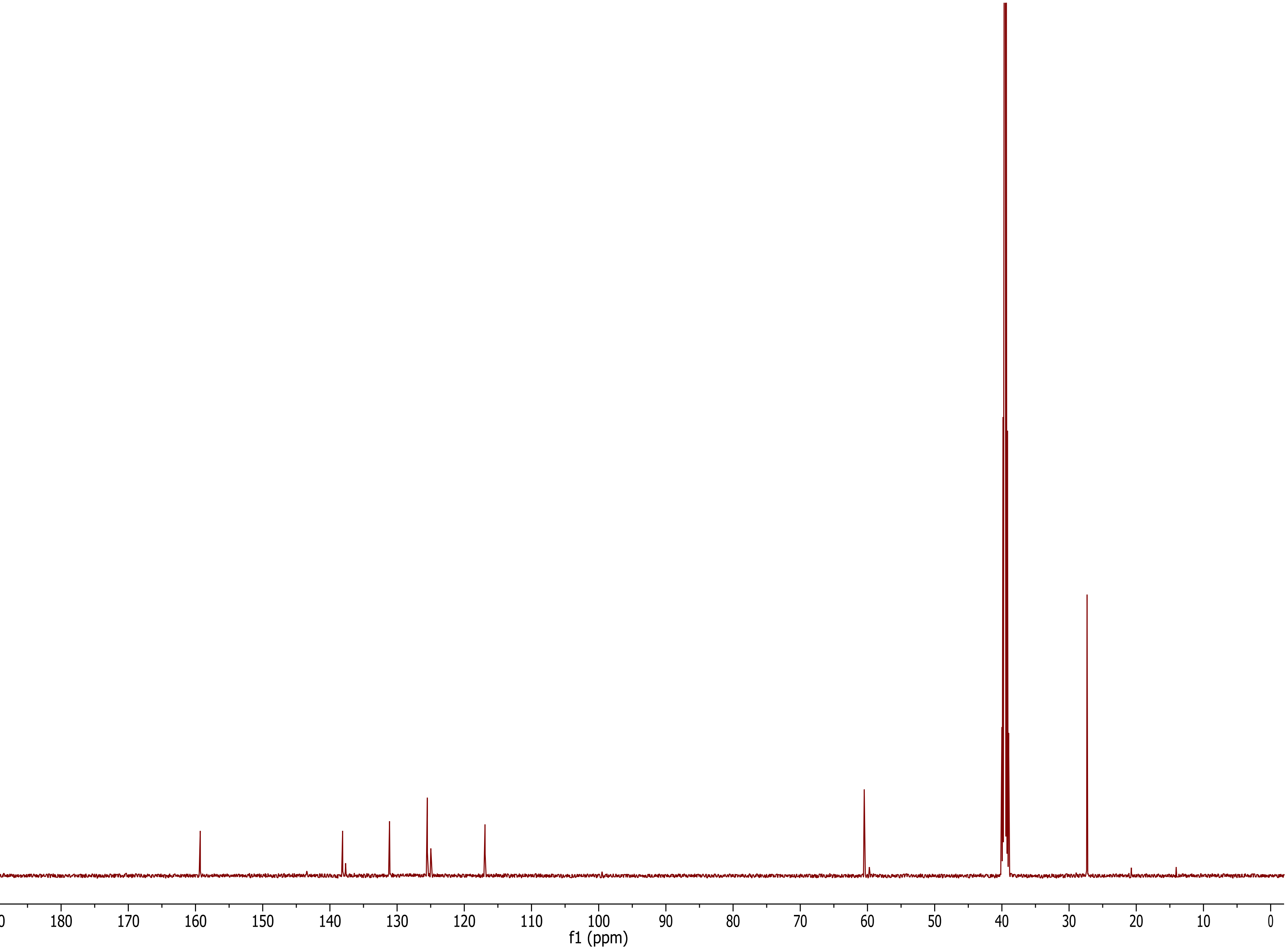

Compound **31** HSQC NMR $(CD_3)_2SO$, 90 °C, coupling constant 160 Hz

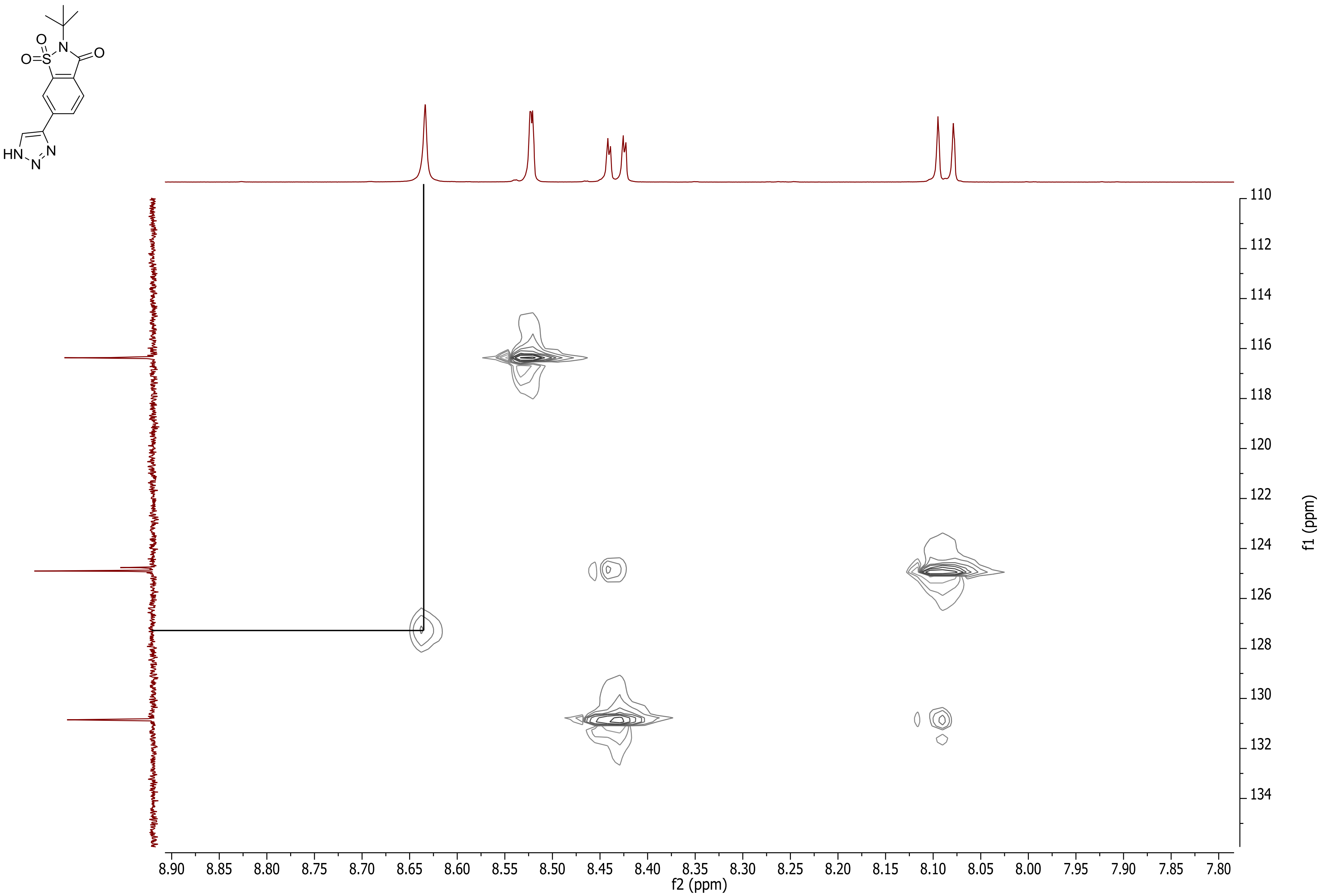

Compound **32a+32b** 500 MHz ^{1}H NMR $(CD_3)_2SO$

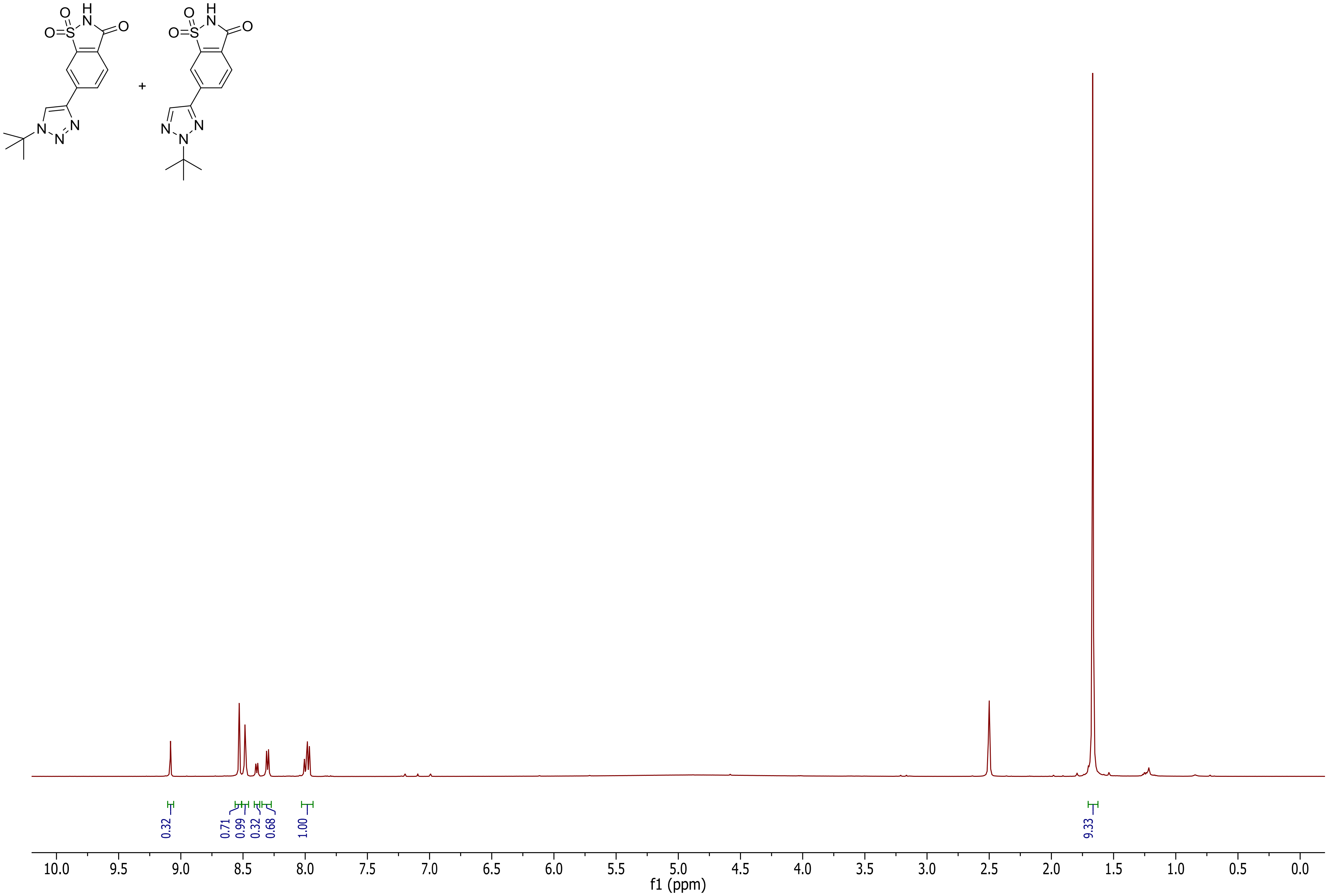

Compound **32a+32b** 125 MHz ^{13}C NMR $(CD_3)_2SO$

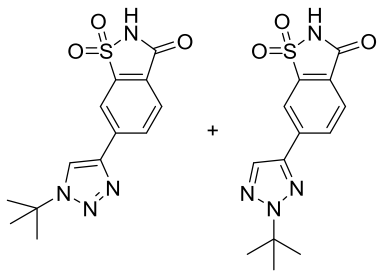

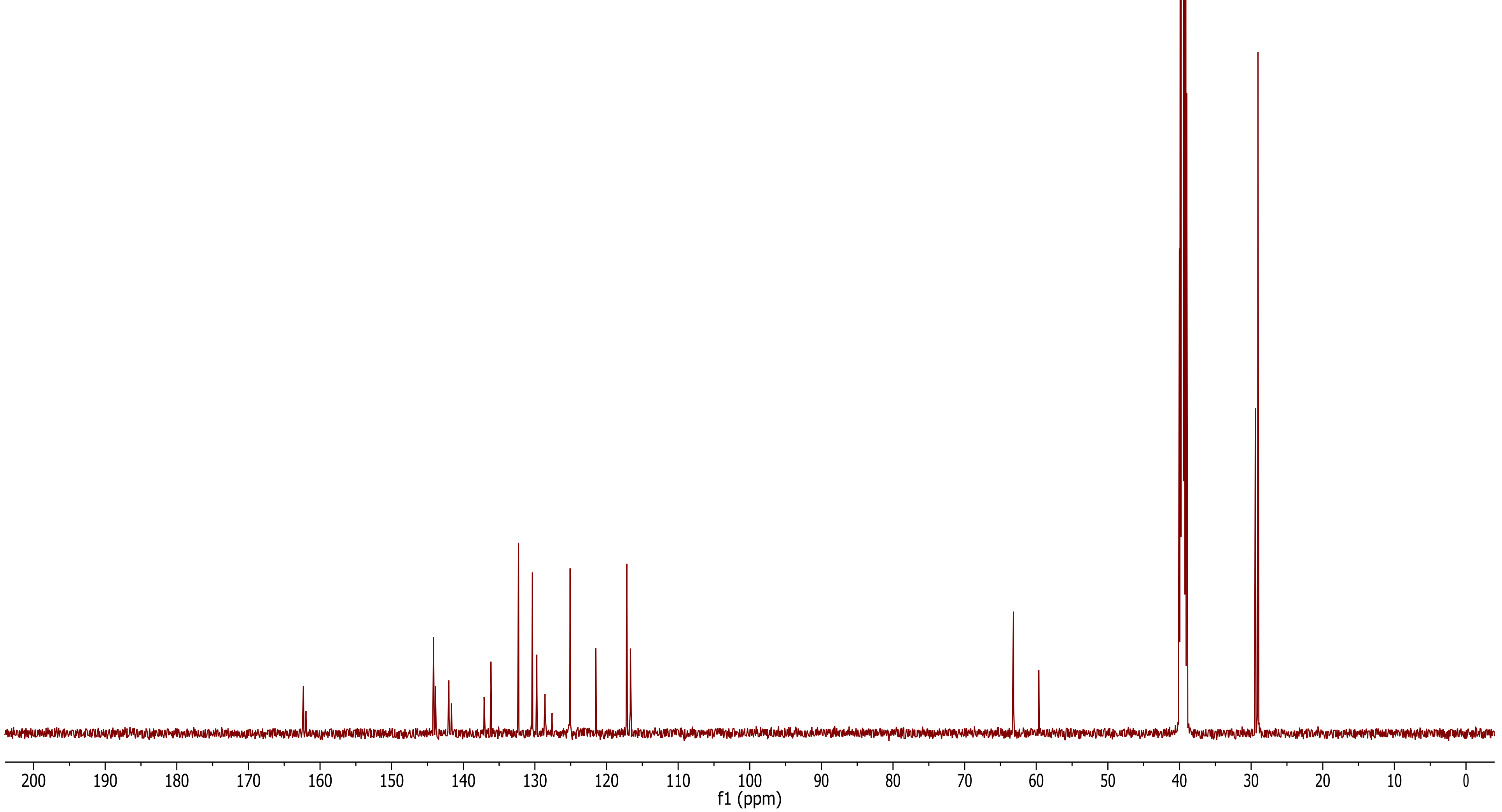

Compound **33** 500 MHz ^{1}H NMR $(CD_3)_2SO$

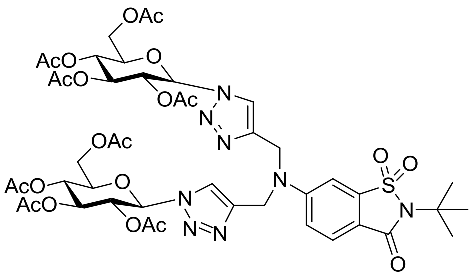

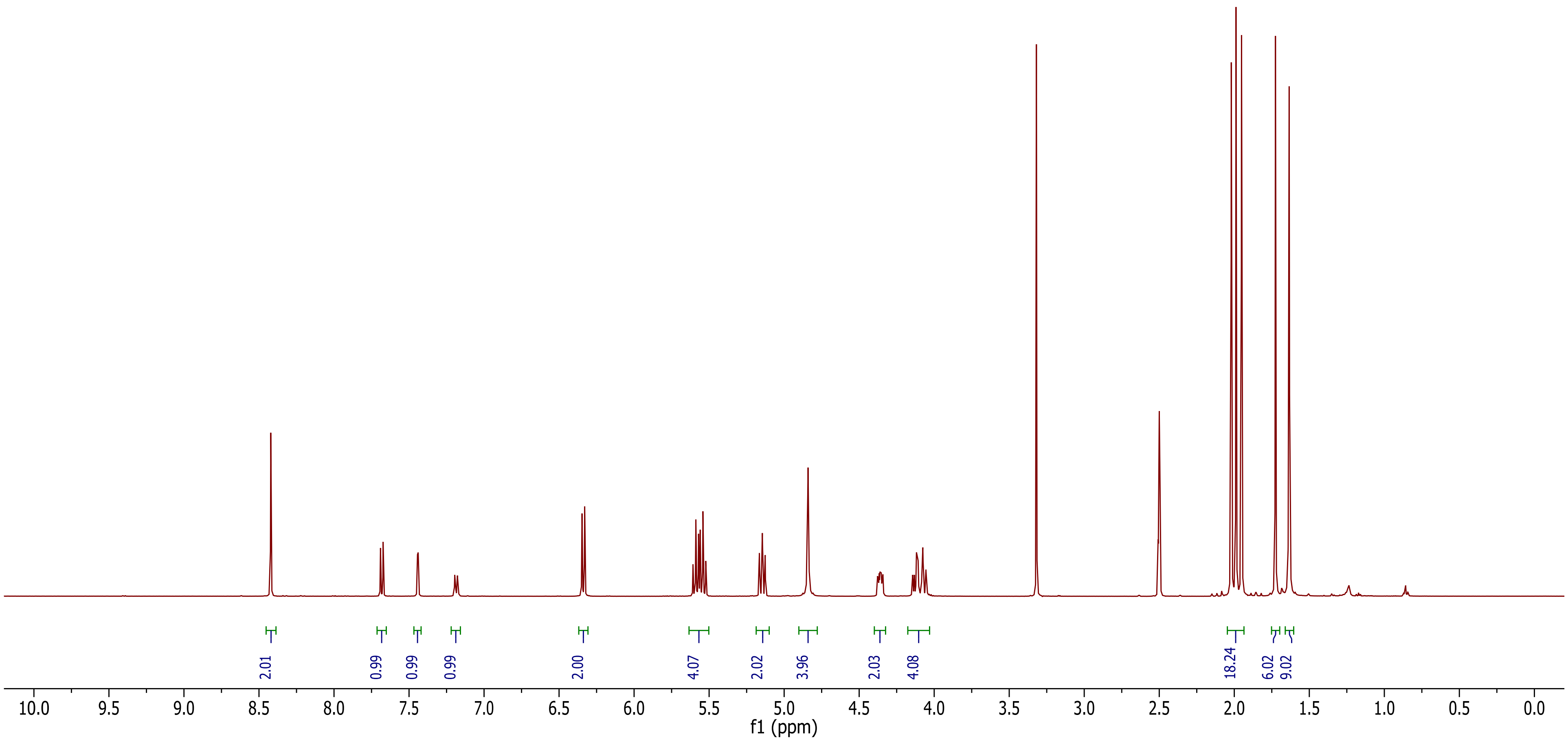

Compound **33** 125 MHz ^{13}C NMR $(CD_3)_2SO$

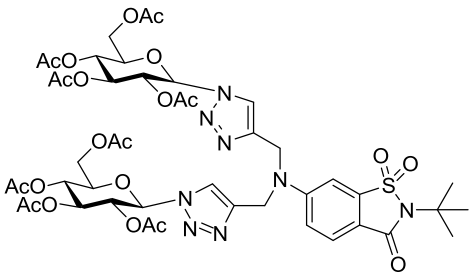

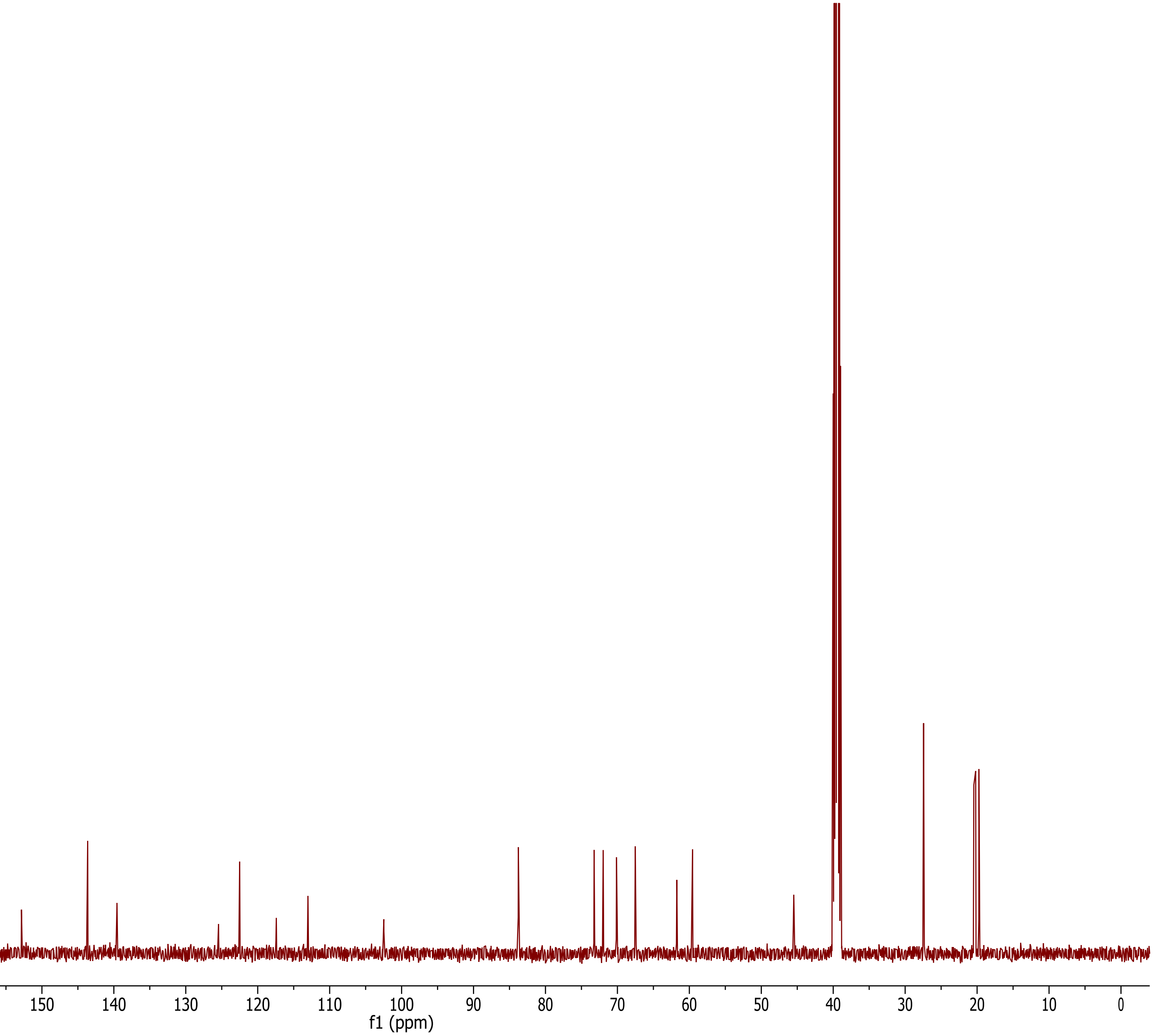

Compound **34** 500 MHz ^{1}H NMR $(CD_3)_2SO$

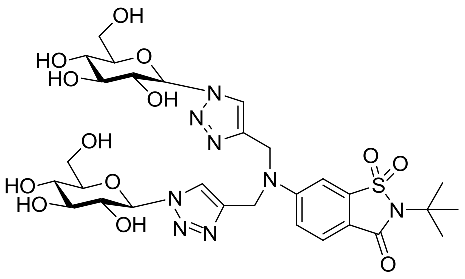

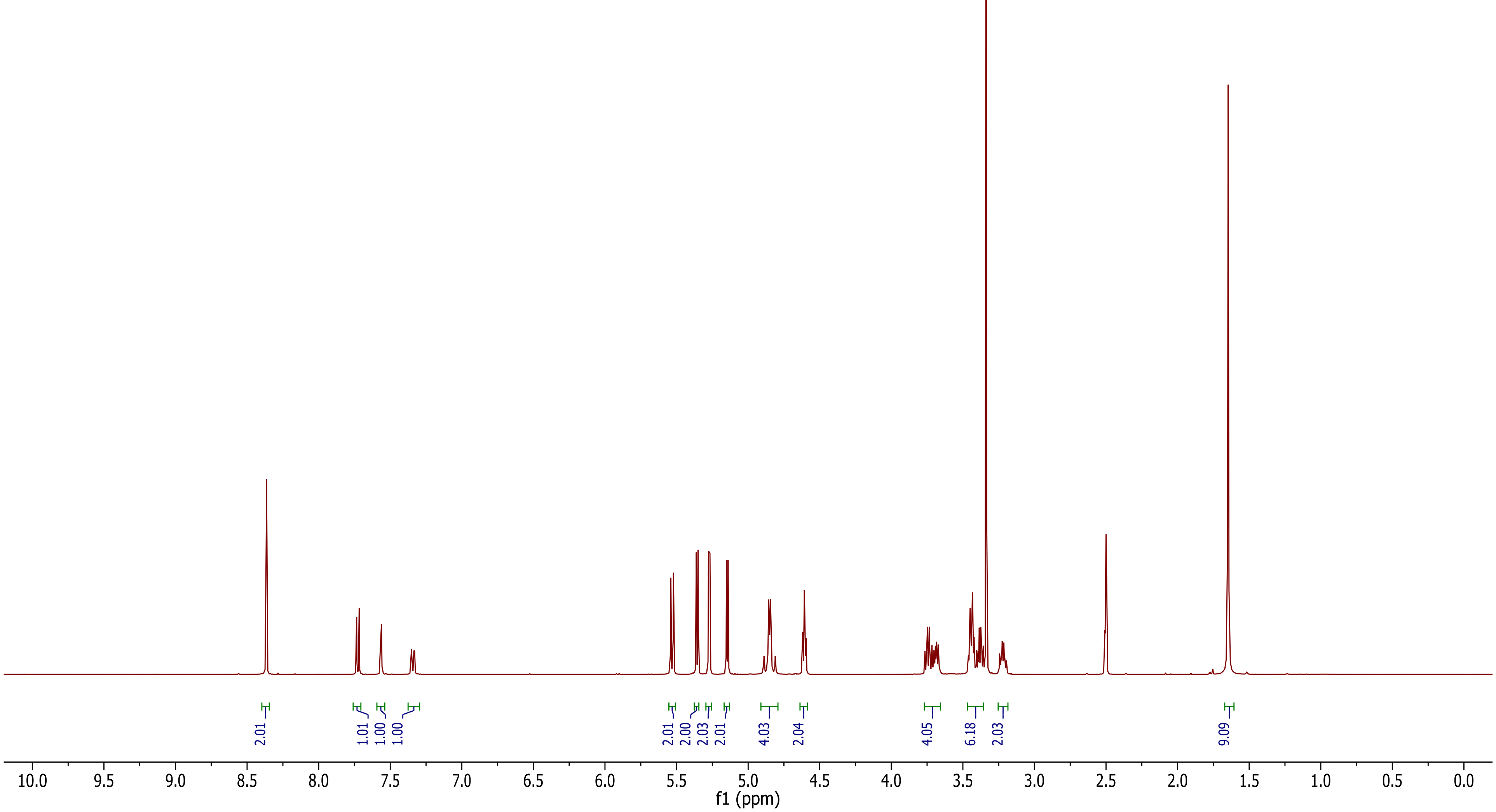

Compound **34** 125 MHz ^{13}C NMR $(CD_3)_2SO$

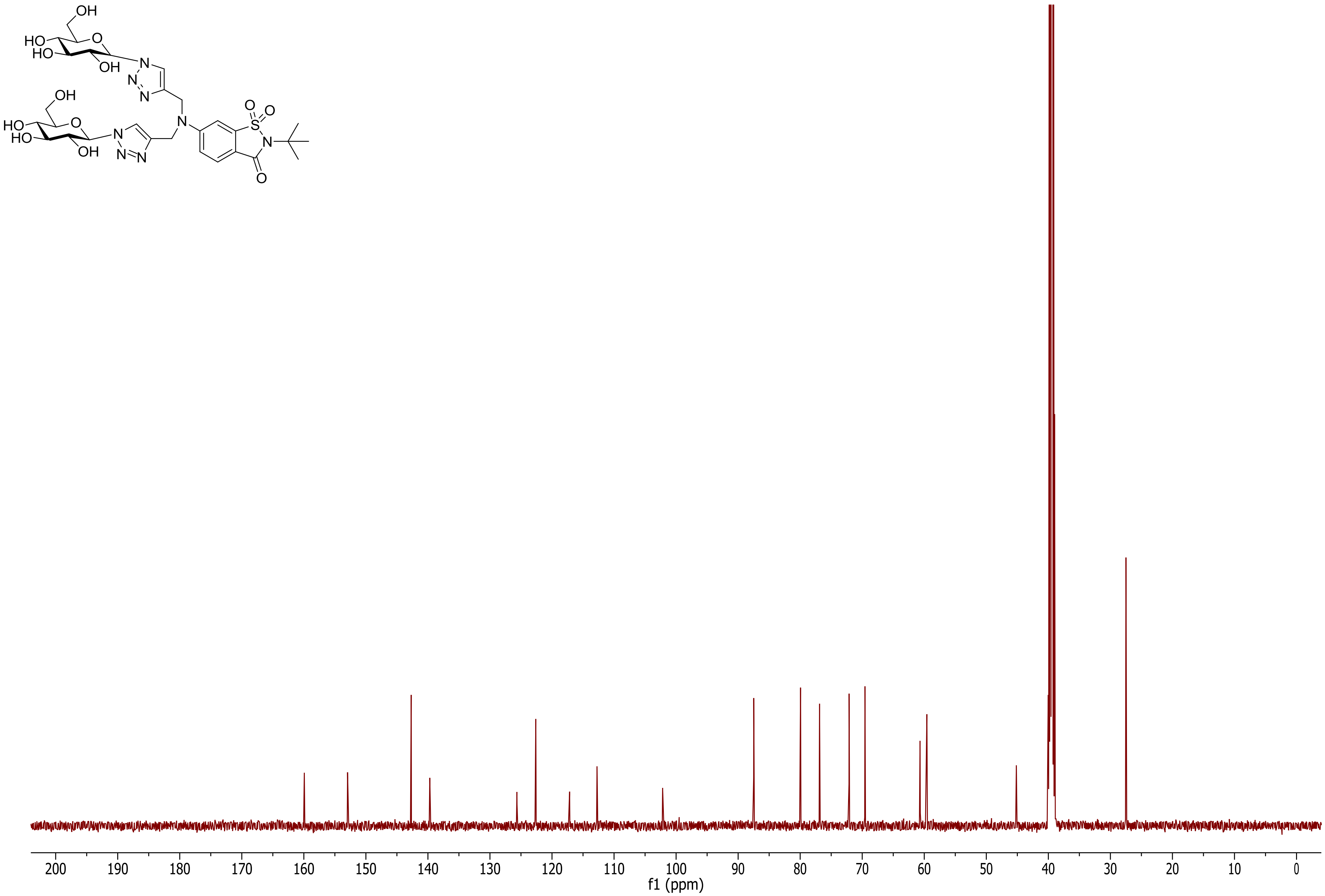

Compound **35** 500 MHz ^{1}H NMR $(CD_3)_2SO$

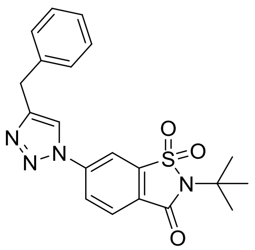

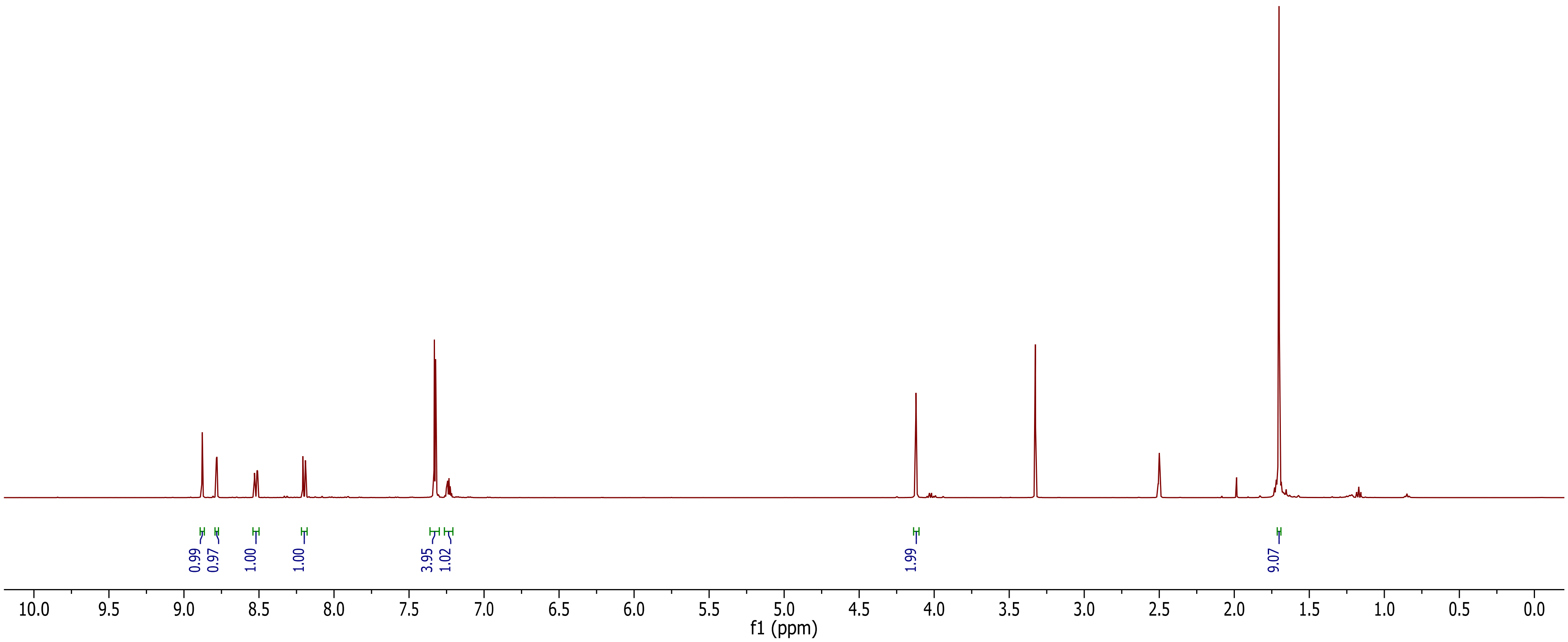

Compound **35** 125 MHz ^{13}C NMR $(CD_3)_2SO$

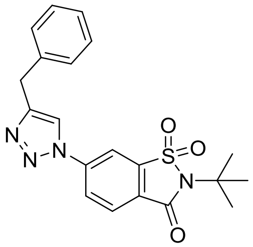

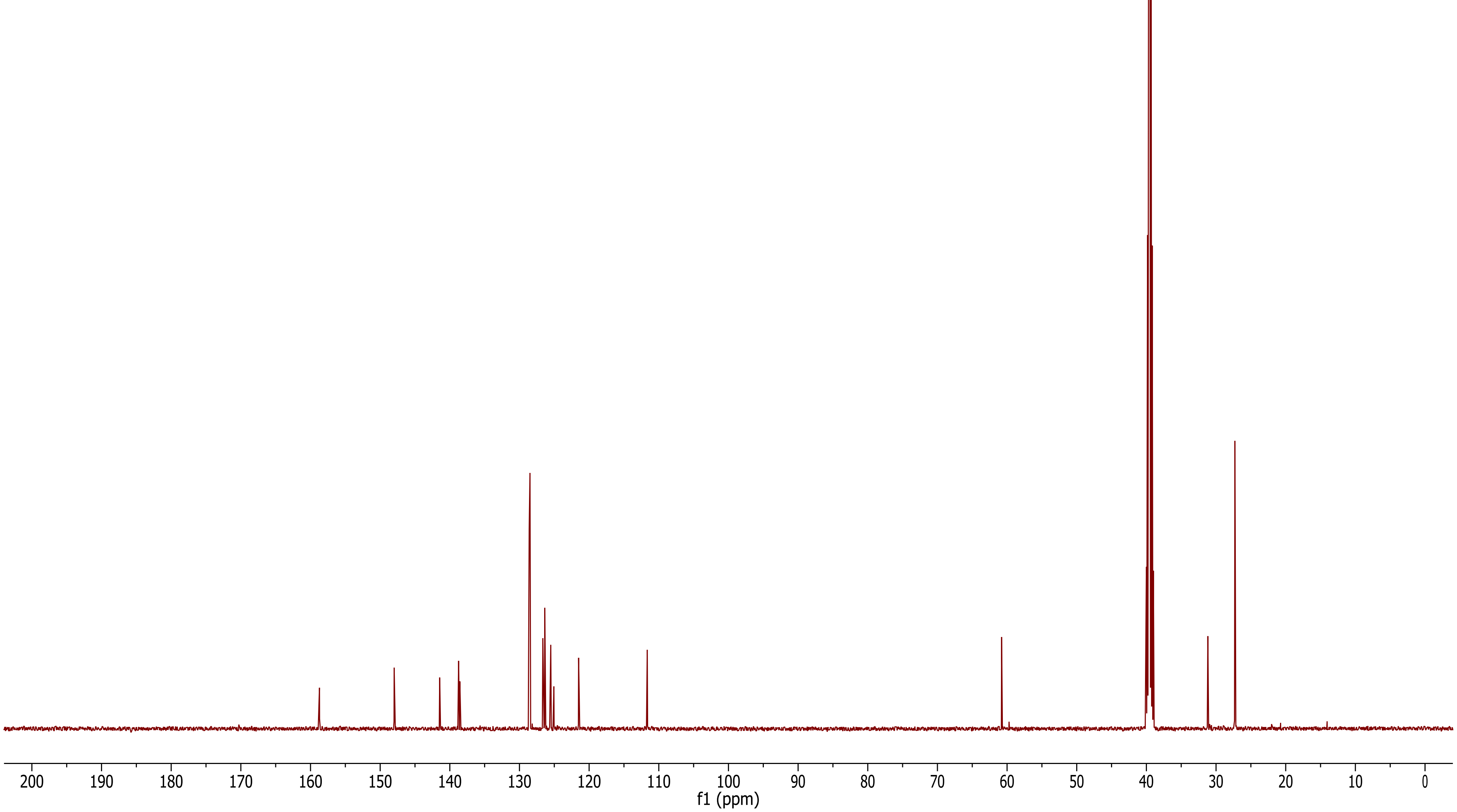

Compound **36** 500 MHz ^{1}H NMR $(CD_3)_2SO$

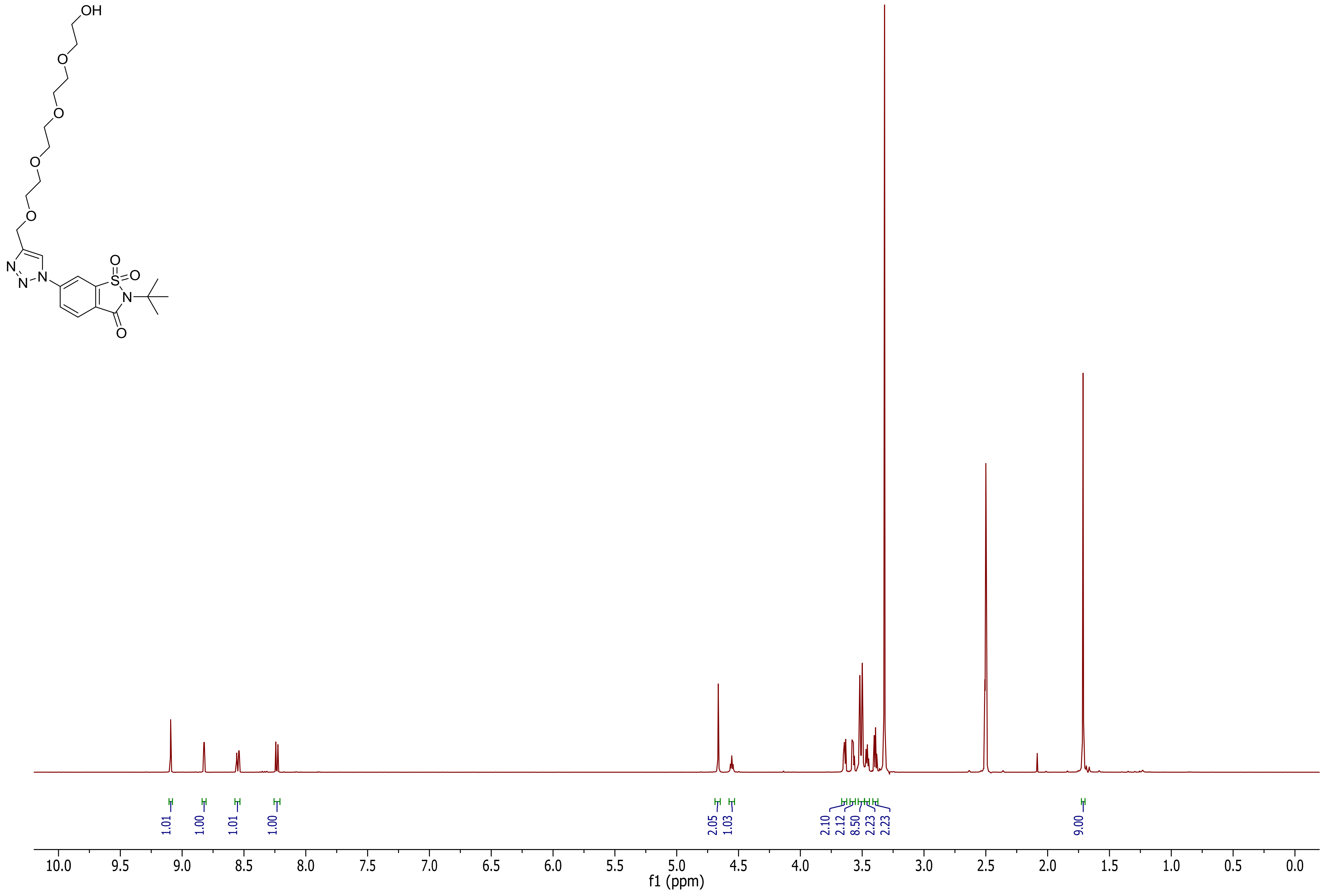

Compound **36** 125 MHz ^{13}C NMR $(CD_3)_2SO$

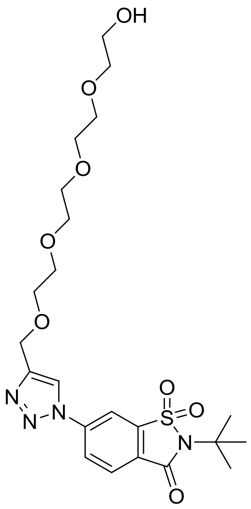

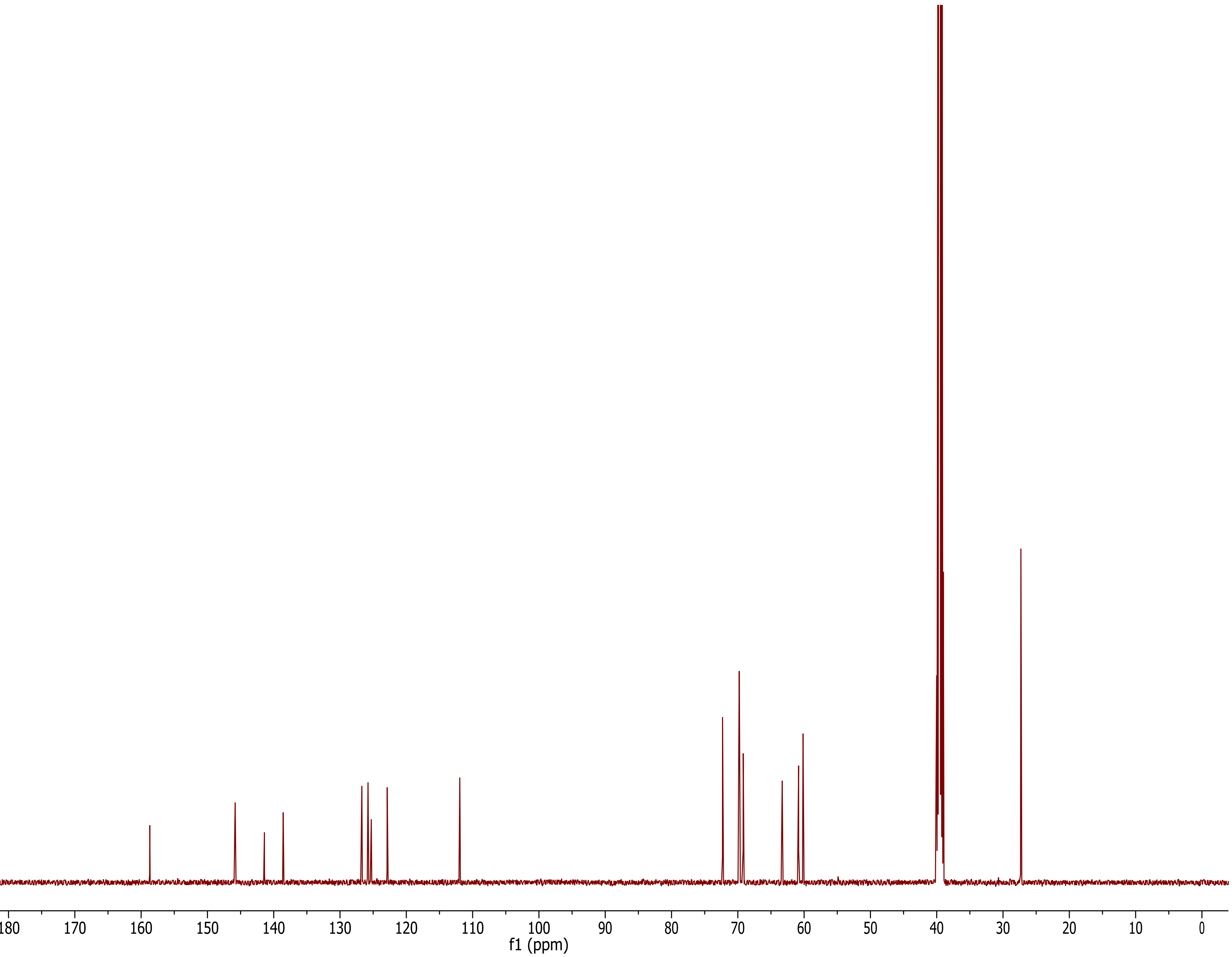

Compound **37** 500 MHz ^{1}H NMR $(CD_3)_2SO$

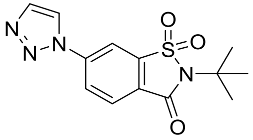

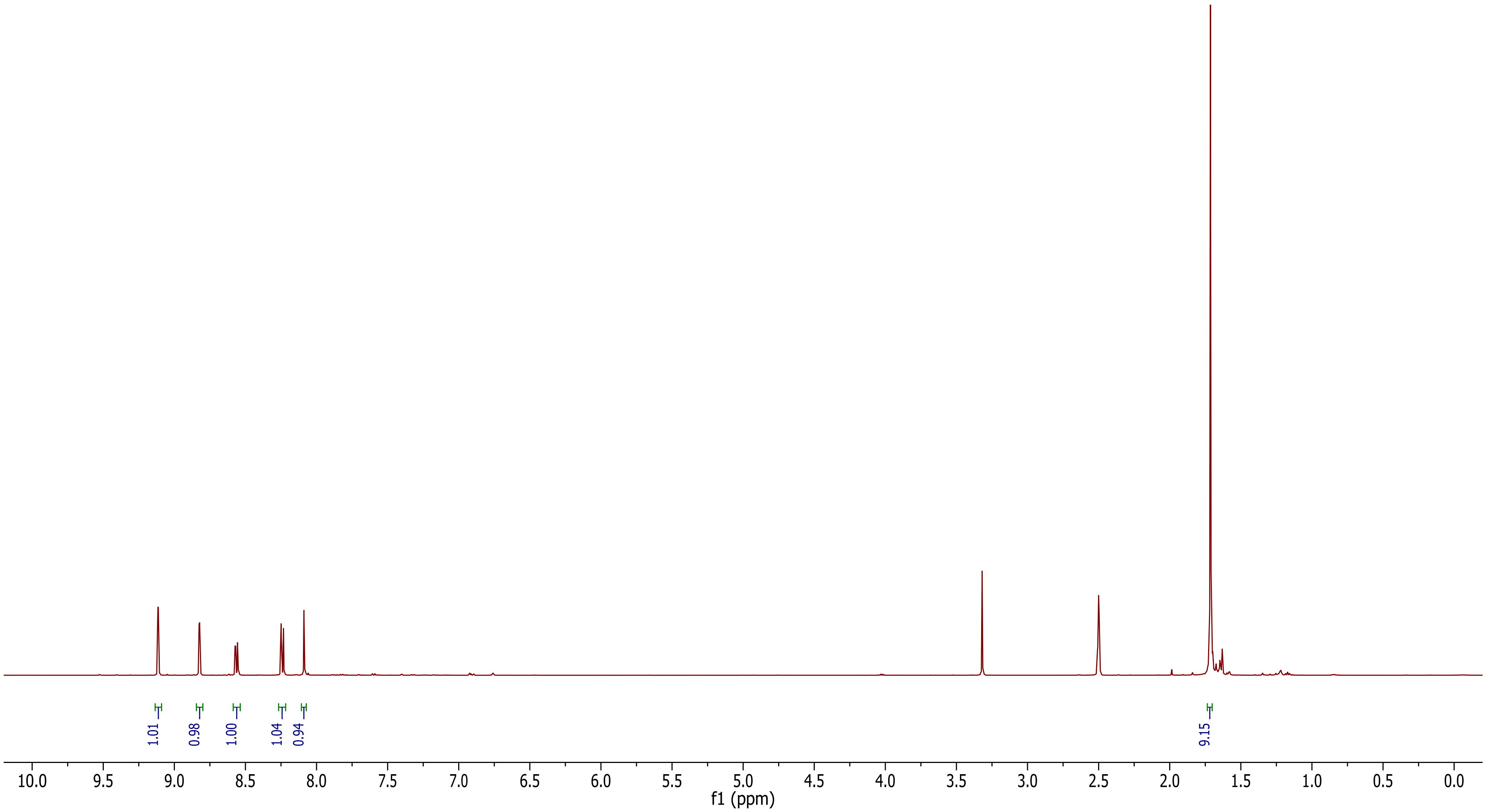

Compound **37** 125 MHz ^{13}C NMR $(CD_3)_2SO$

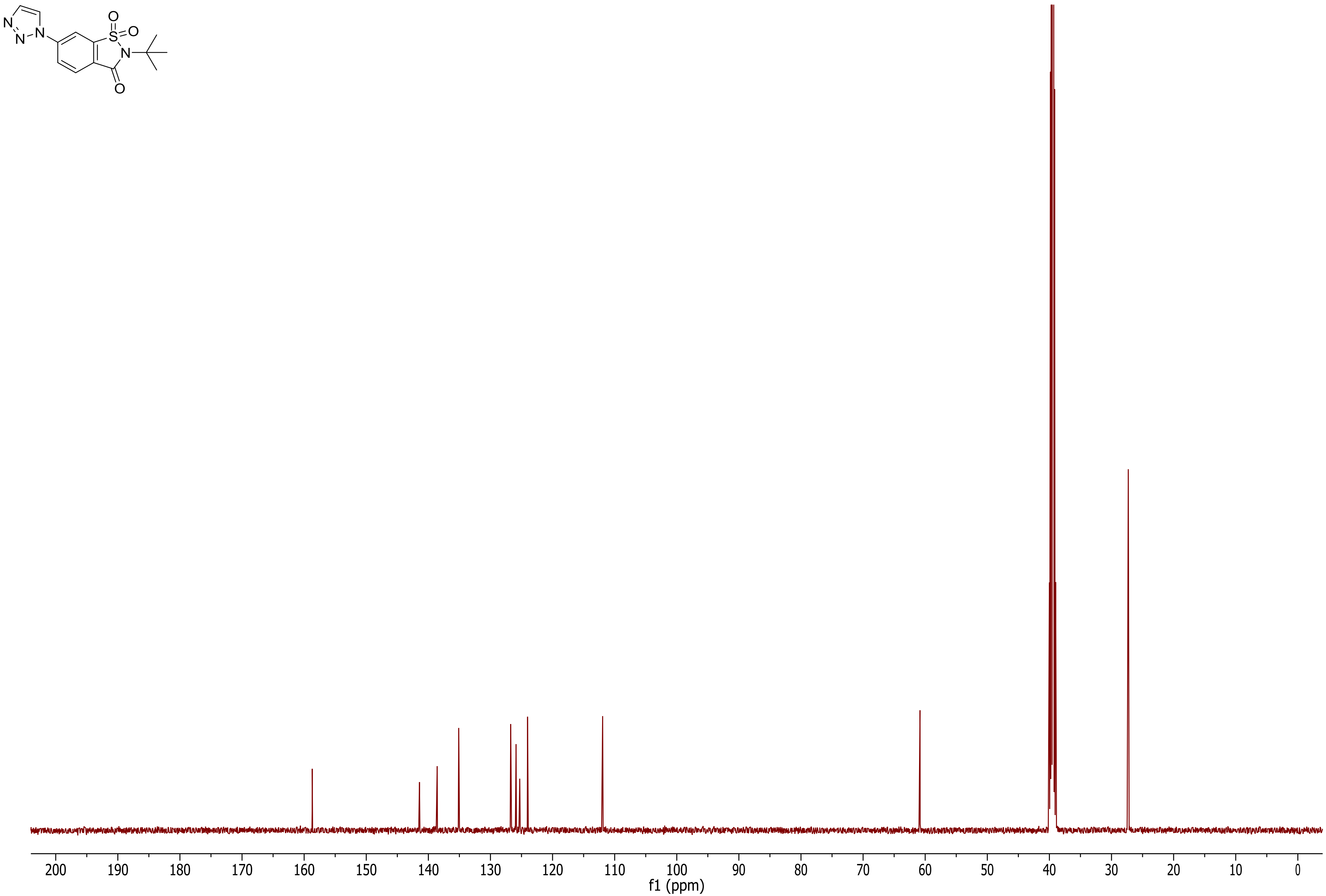

Compound **38** 500 MHz ^{1}H NMR $(CD_3)_2SO$

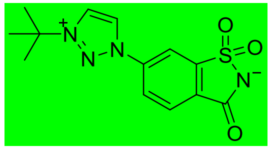

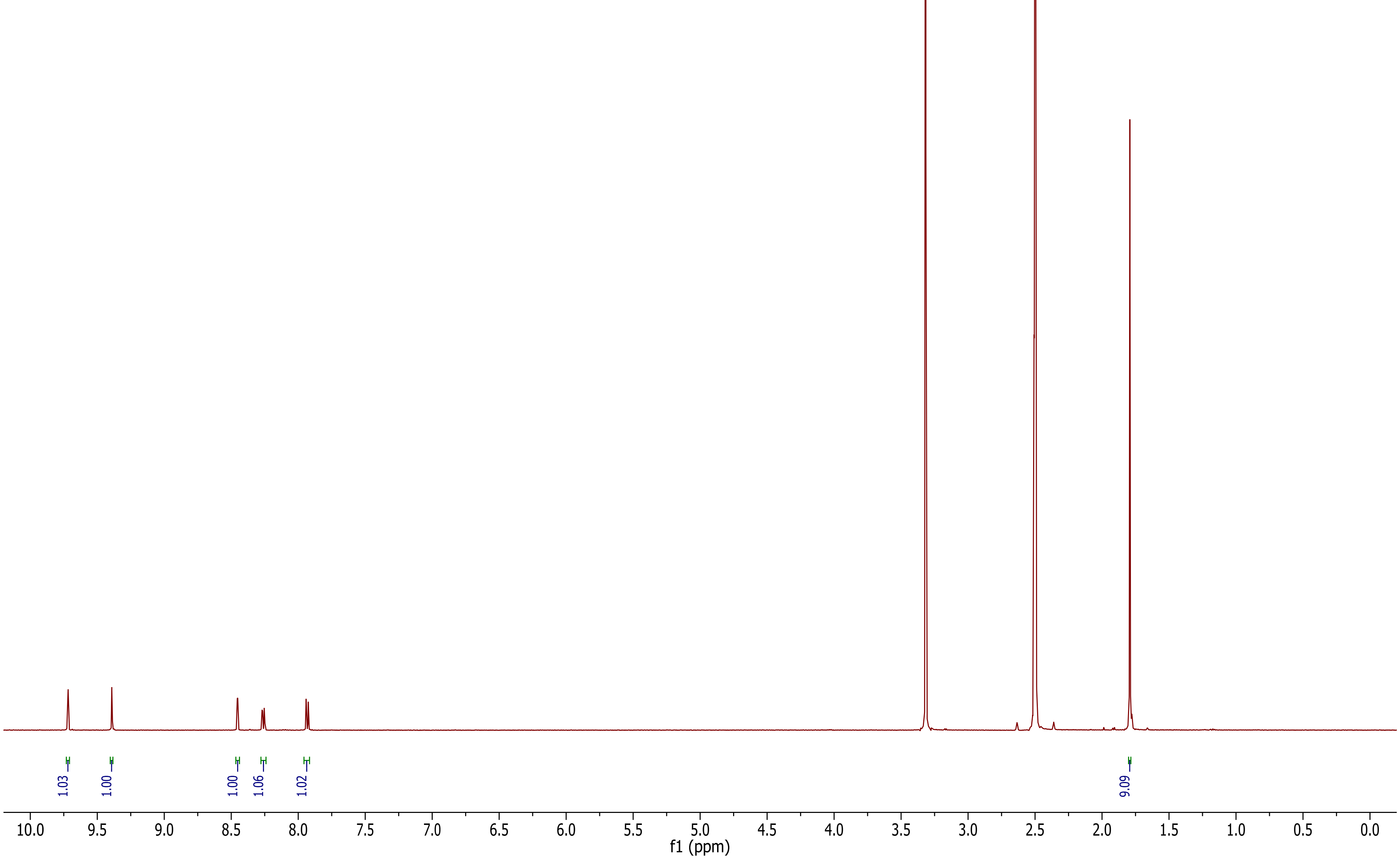

Compound **38** 125 MHz ^{13}C NMR $(CD_3)_2SO$

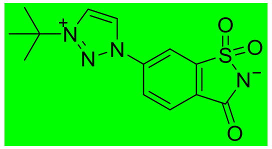

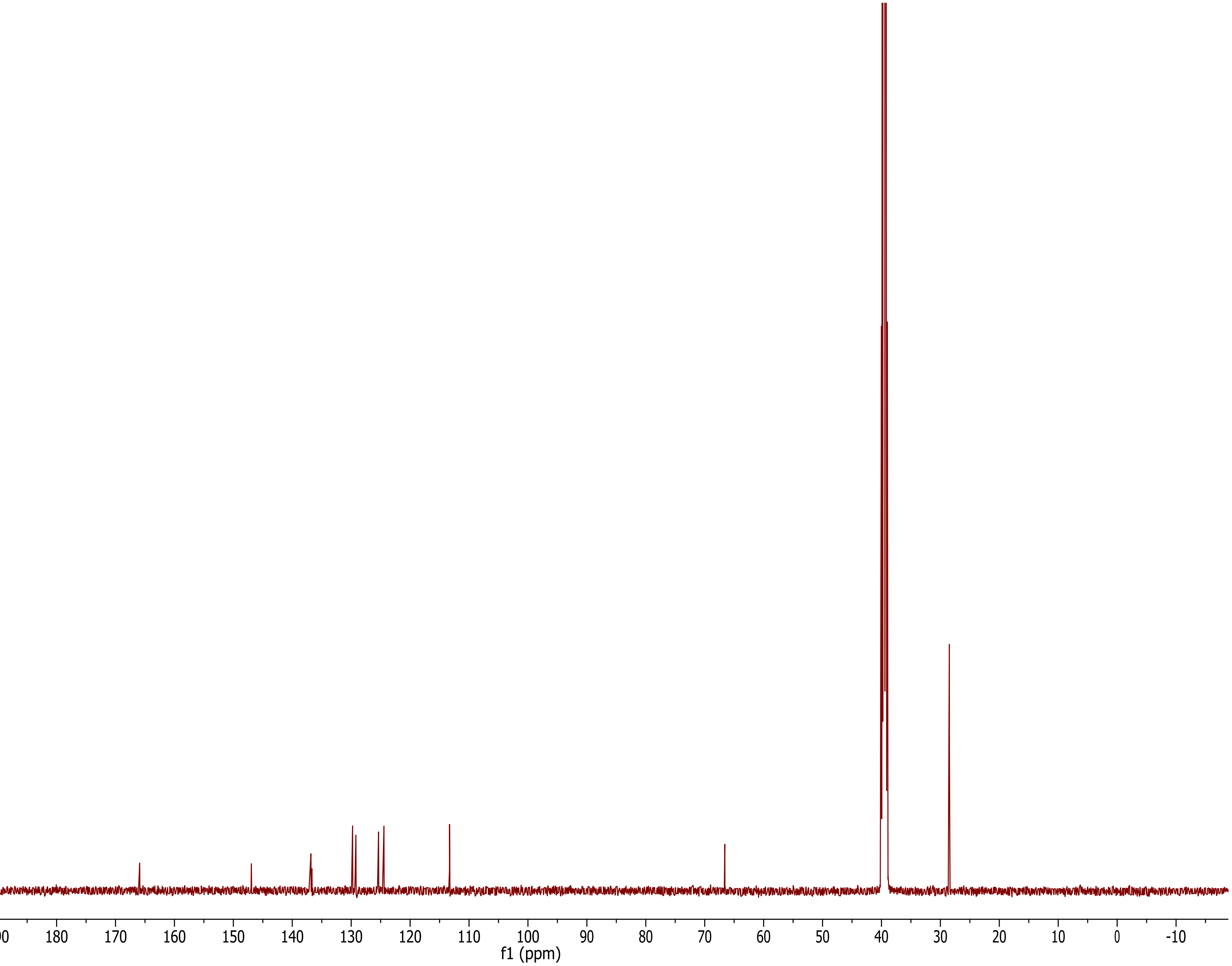

Compound **39** 500 MHz ^{1}H NMR $(CD_3)_2SO$

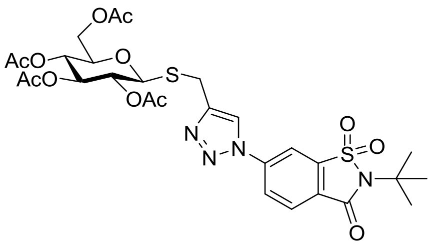

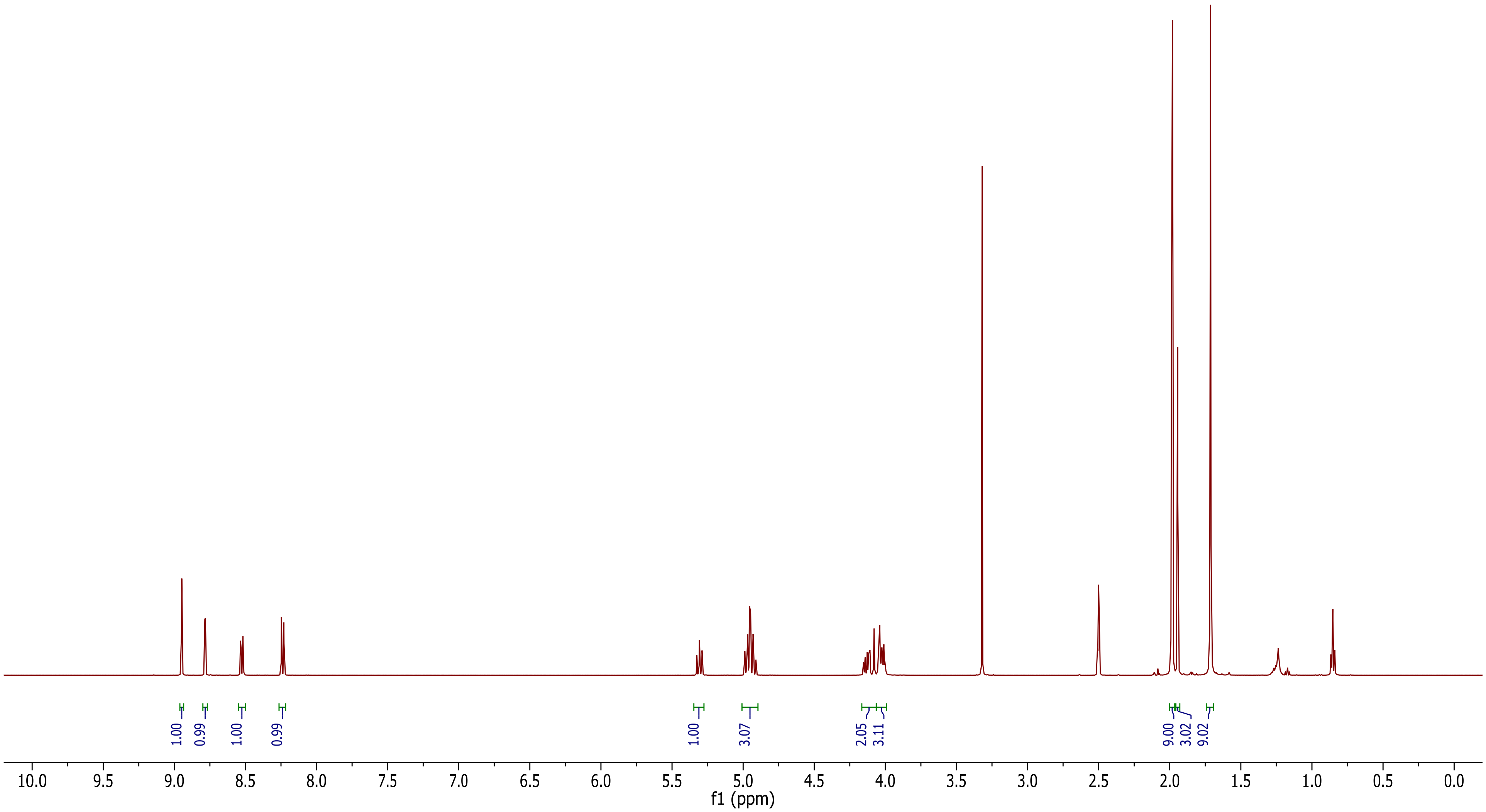

Compound **39** 125 MHz ^{13}C NMR $(CD_3)_2SO$

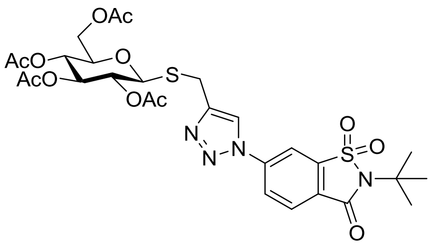

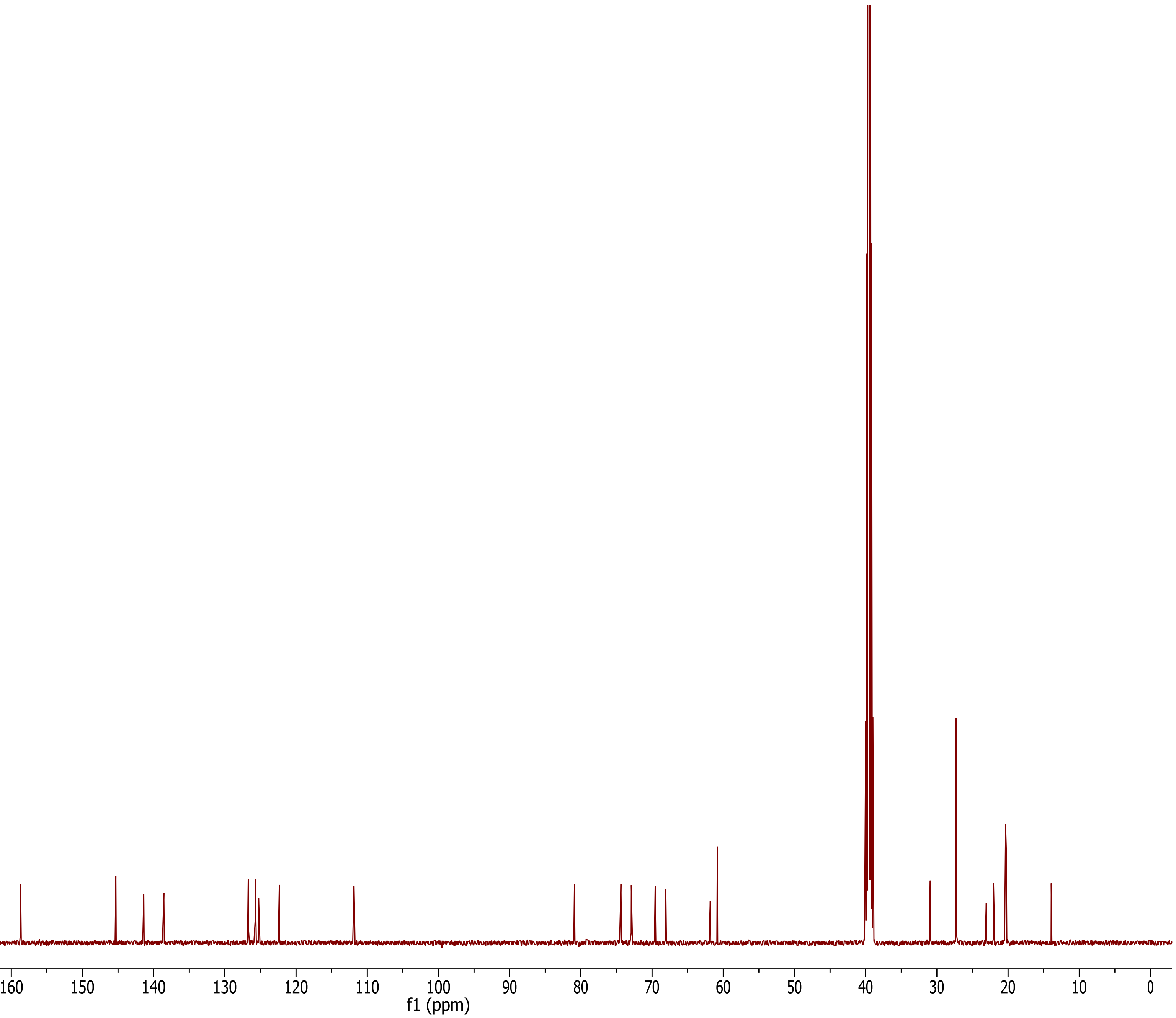

Compound **40** 500 MHz ^{1}H NMR $(CD_3)_2SO$

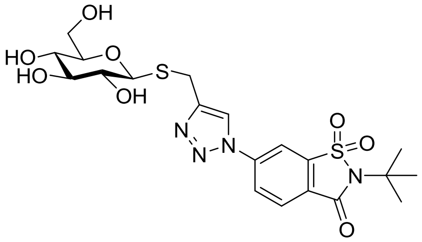

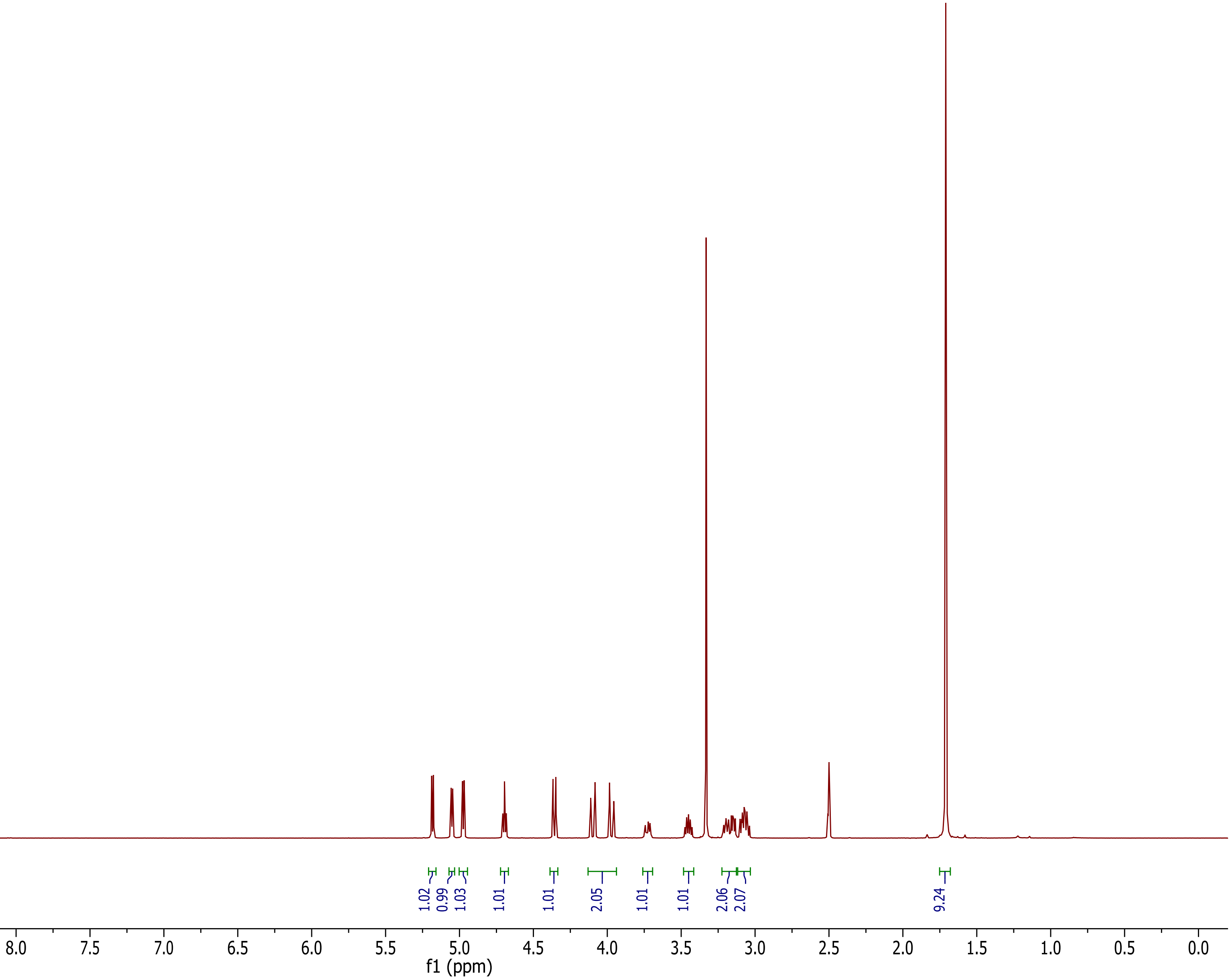

Compound **40** 125 MHz ^{13}C NMR $(CD_3)_2SO$

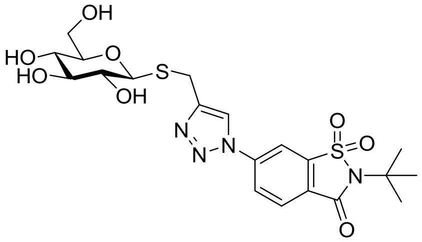

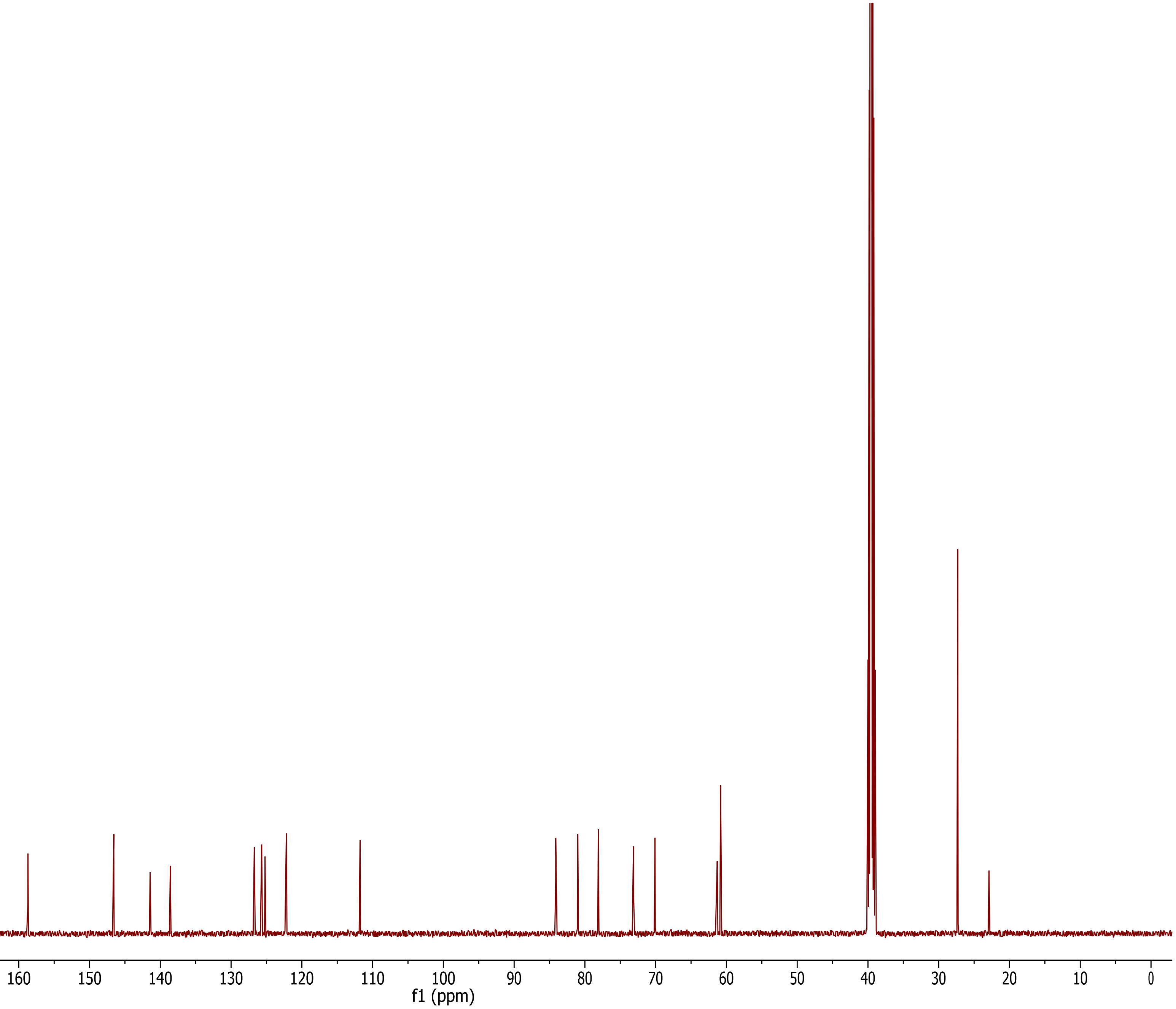

Compound **41** 500 MHz ^{1}H NMR $(CD_3)_2SO$

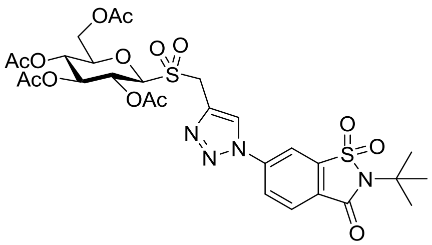

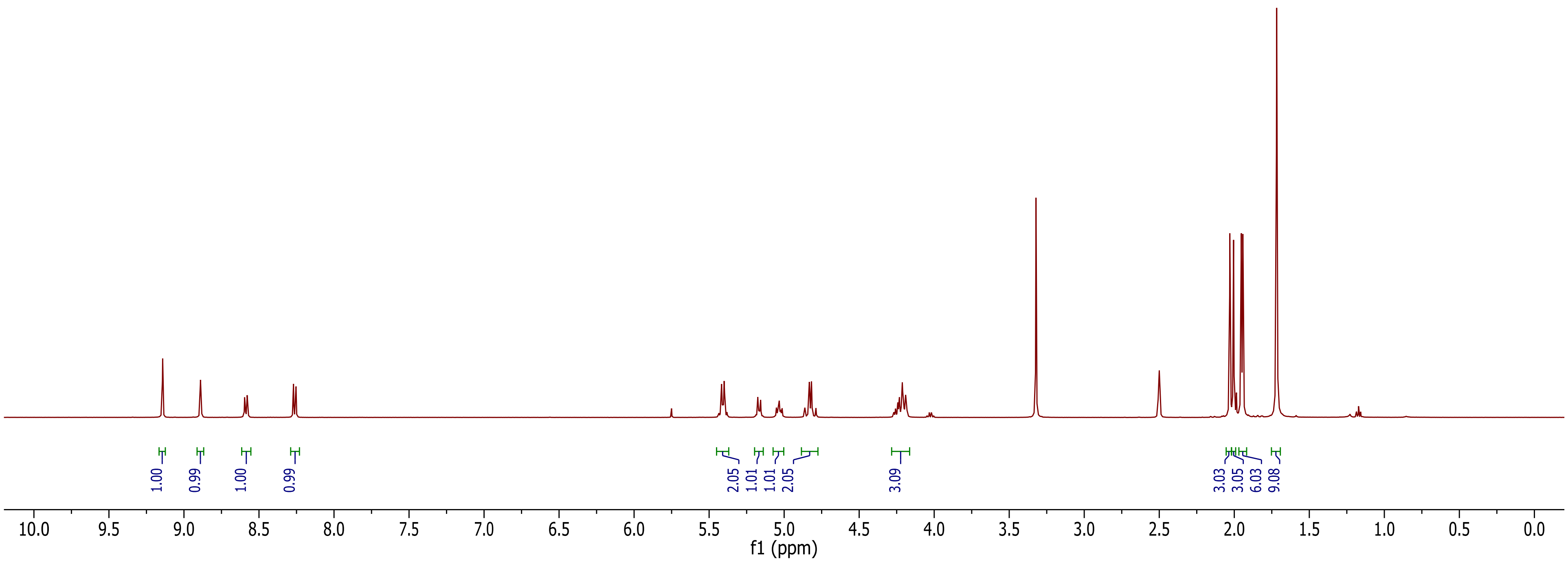

Compound **41** 125 MHz ^{13}C NMR $(CD_3)_2SO$

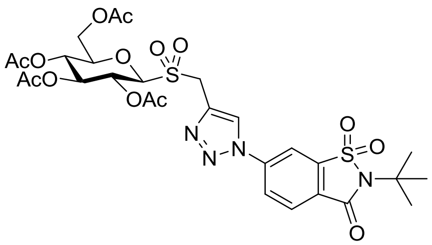

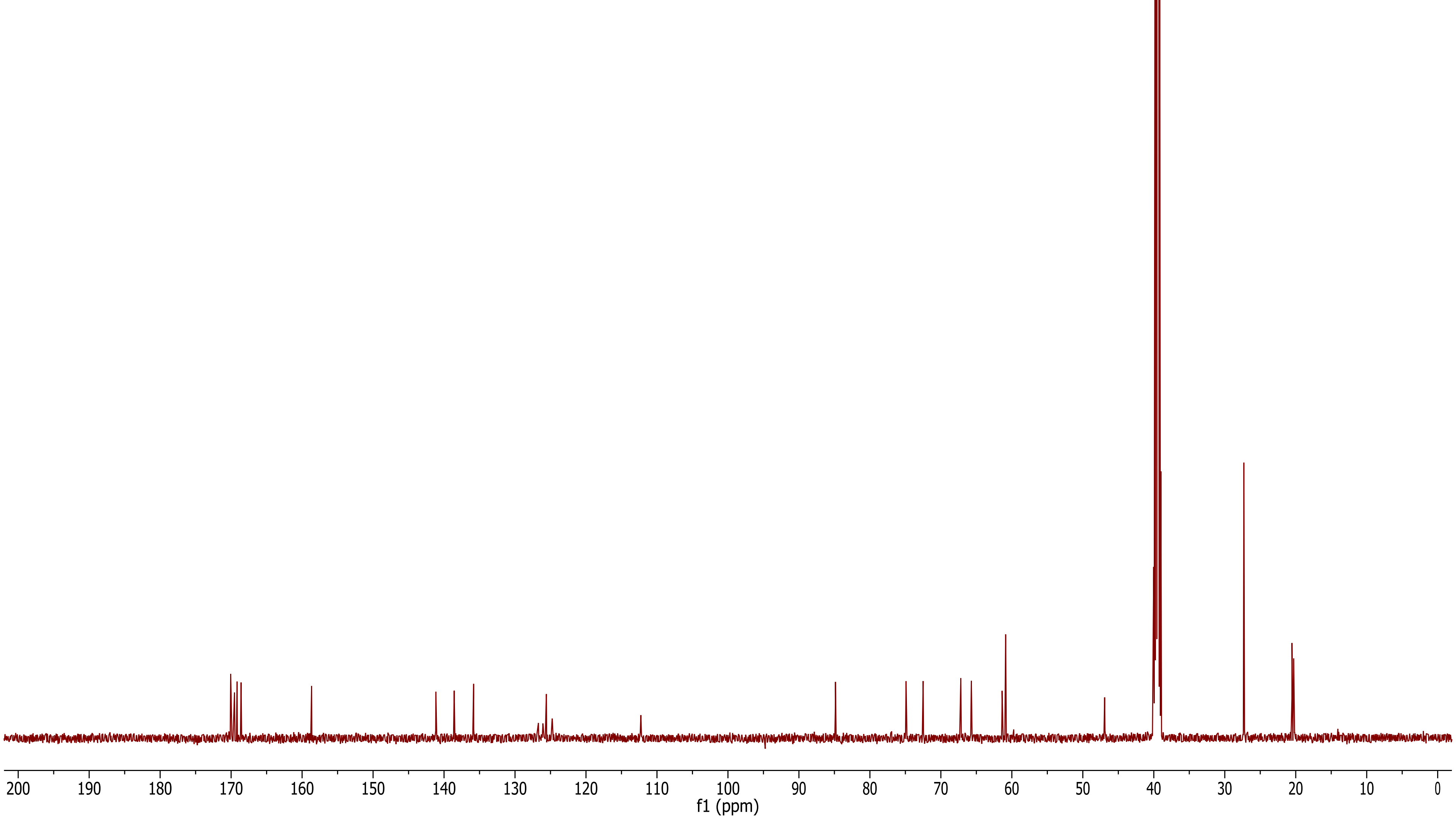

Compound **42** 500 MHz ^{1}H NMR $(CD_3)_2SO$

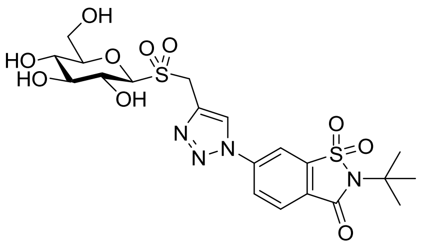

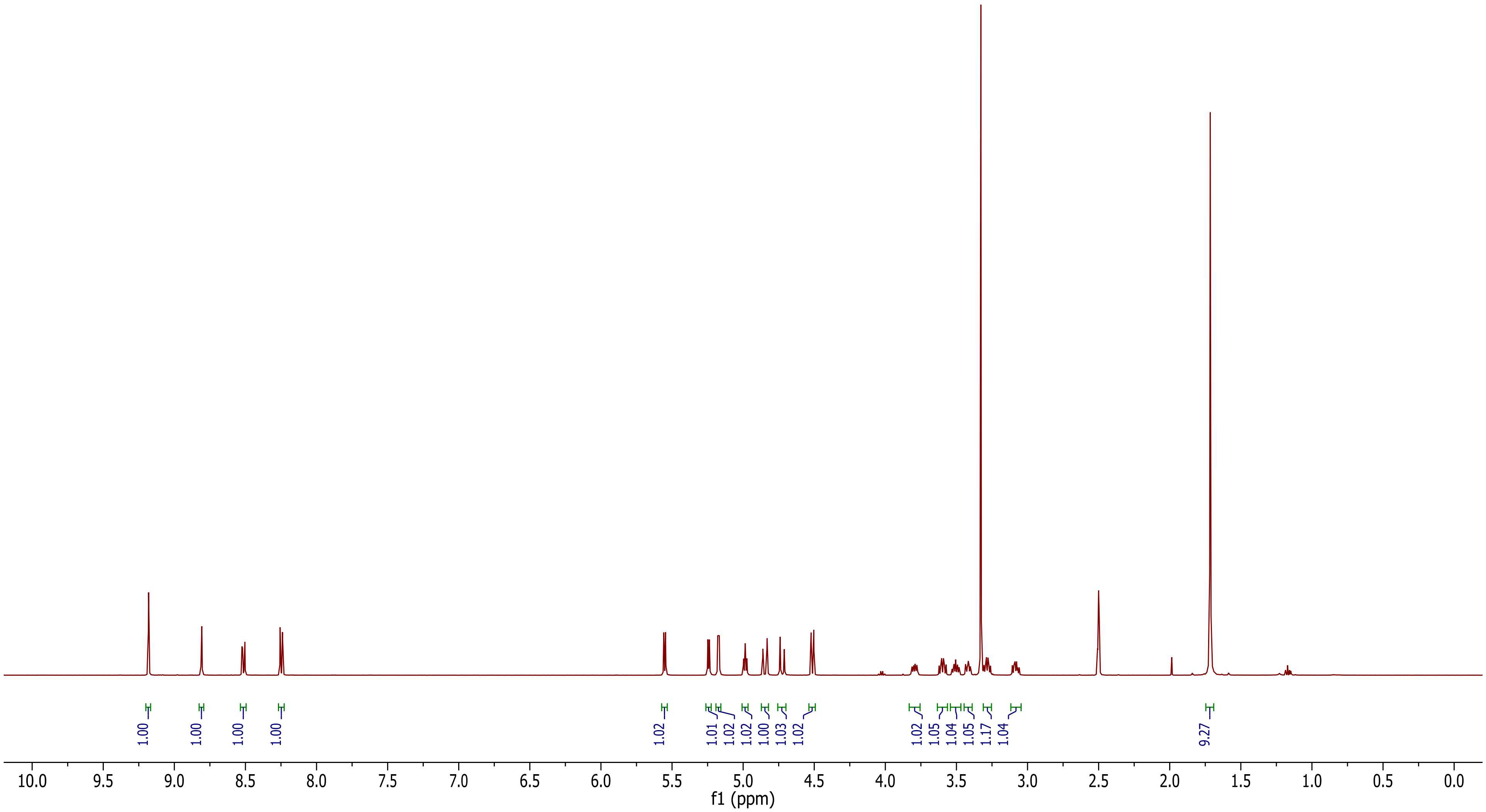

Compound **42** 125 MHz ^{13}C NMR $(CD_3)_2SO$

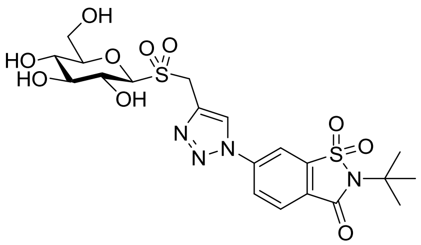

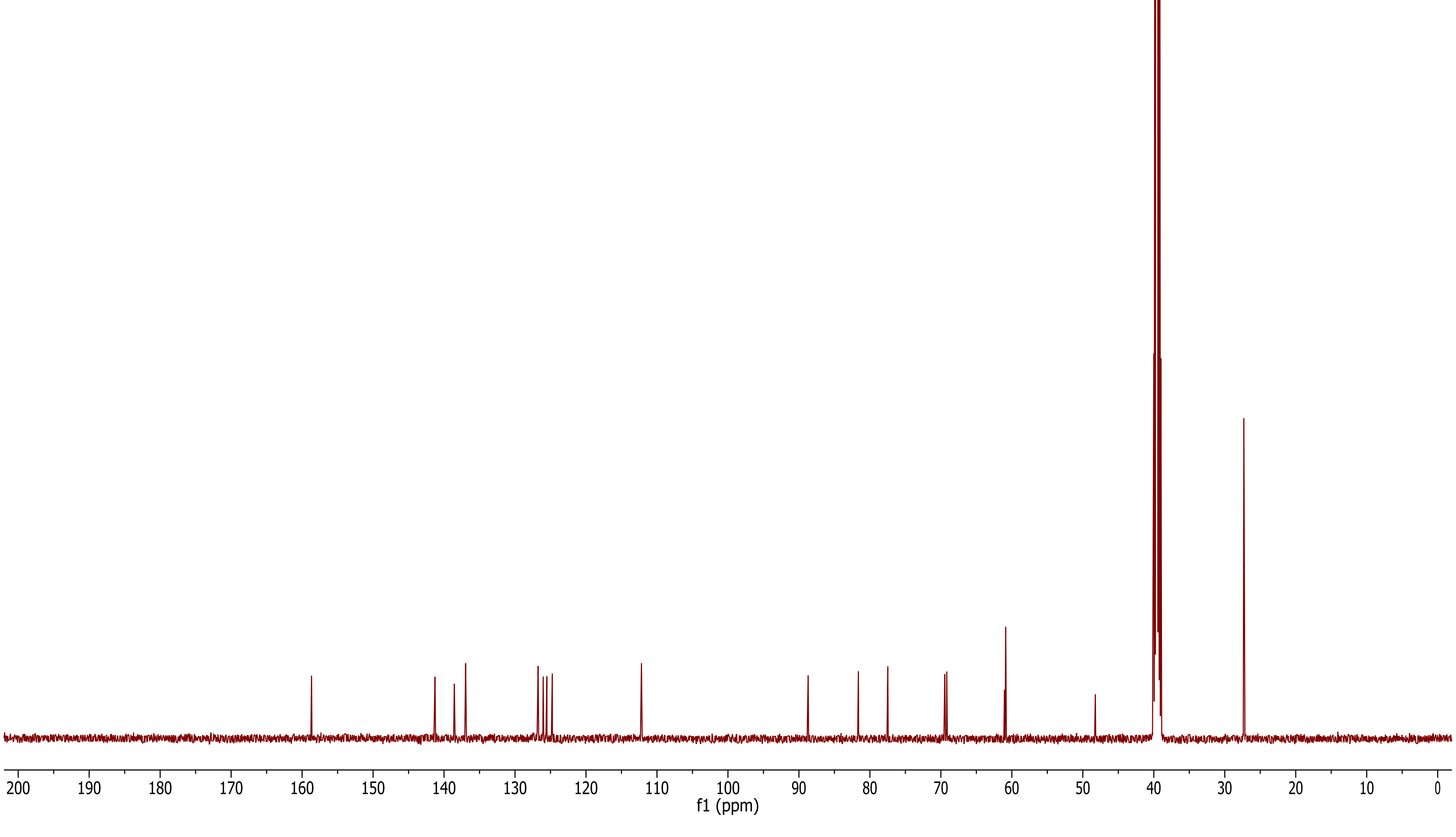

Supplement: Supplementary file 1 [file molecules-22-00516-s001.pdf]
